# Anticancer Action of Xiaoxianxiong Tang in Non-Small Cell Lung Cancer by Pharmacological Analysis and Experimental Validation

**DOI:** 10.1155/2021/9930082

**Published:** 2021-12-13

**Authors:** Rongzhen Ding, Lijing Jiao, Shuliu Sang, Yinan Yin, Yichao Wang, Yabin Gong, Ling Xu, Ling Bi

**Affiliations:** ^1^Department of Oncology, Yueyang Hospital of Integrated Traditional Chinese and Western Medicine, Shanghai University of Traditional Chinese Medicine, Shanghai, China; ^2^Institute of Clinical Immunology, Yueyang Hospital of Integrated Traditional Chinese and Western Medicine, Shanghai University of Traditional Chinese Medicine, Shanghai, China

## Abstract

Xiaoxianxiong Tang (XXXT) is a well-known traditional Chinese medicine formula. Evidence is emerging supporting the benefits of XXXT in ameliorating therapy for non-small cell lung cancer (NSCLC). The purpose of this study aimed to explore the effects and mechanisms of XXXT through network pharmacological analysis and biological validation. TCMSP database was used to identify potentially active compounds in XXXT with absorption, distribution, metabolism, excretion screening, and their potential targets. The disease targets related to NSCLC were predicted by searching for Therapeutic Target database, GeneCards database, DrugBank database, and DisGeNET database. Of the 4385 NSCLC-related targets, 156 targets were also the targets of compounds present in XXXT. Subsequently, GO function and KEGG pathway enrichment and PPI network analyses revealed that, of the 95 targets and 20 pathways influenced by 20 ingredients in XXXT, 20 targets were associated with patient survival, and XXXT could exert an inhibitory action on the PI3K-AKT signaling pathway. Moreover, XXXT restrained the proliferation of A549 and H460 cells in a concentration-dependent manner and suppressed the mRNA and protein levels of key targets CCNA2, FOSL2, and BIRC5 closely linked to the PI3K-AKT pathway. Hence, XXXT has the potential to improve therapy for NSCLC by targeting the PI3K-AKT signaling pathway.

## 1. Introduction

Lung cancer is the major cause of cancer-related deaths all over the world, which lies behind almost one-quarter of all cancer deaths [1, 2]. Non-small cell lung cancer (NSCLC) is the main subtype of lung cancer, and its proportion is approximately 80% in all lung cancer cases. The propensity for recurrence and distant metastasis of NSCLC leads to poor prognosis [3]. It is worth noting that recurrence and metastasis also occur following target therapy and recent immunotherapy [4, 5]. Moreover, some conventional therapies are accompanied by serious side effects, thus reducing the life quality of patients [6]. Therefore, it is necessary to explore novel drugs for the treatment of NSCLC.

TCM has been employed for fighting cancer for thousands of years [7, 8]. Xiaoxianxiong Tang (XXXT) is a prescription that was first recorded in the Treatise on Exogenous Febrile Disease (Shanghan Lun) in the Eastern Han Dynasty 1700 years ago. XXXT is composed of 3 herbs, including *Coptidis Rhizoma* (Huanglain in Chinese), *Arum Ternatum Thunb* (Banxia in Chinese), and *Trichosanthes Kirilowii Maxim* (Gualou in Chinese). Traditionally, XXXT was used to dissipate phlegm and reduce lumps. Recently, it has been reported that XXXT or its active ingredients exert antiproliferative effects on a variety of tumor cells [9–14]. However, the active components of XXXT and their molecular mechanisms on NSCLC are yet to be determined.

**Figure 1:**
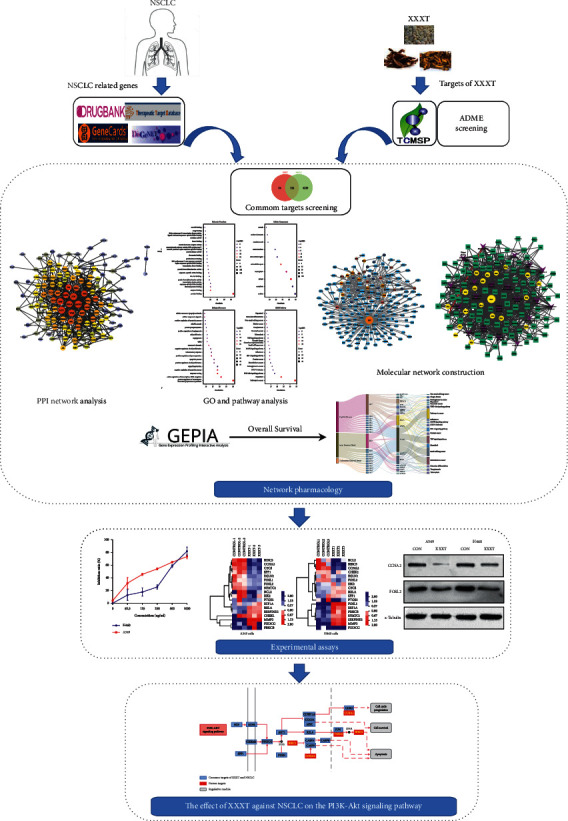
The overall structure flowchart of the study based on pharmacological analysis and experimental validation for deciphering the mechanism of XXXT on NSCLC.

In order to understand the molecular mechanisms of XXXT in the anticancer action in NSCLS, we used network pharmacology to identify the active components present in XXXT and their potential targets. By interrogating the disease targets related to NSCLC, a pool of common targets was revealed and validated experimentally. The overall structure flowchart is shown in Figure 1.

## 2. Materials and Methods

### 2.1. Predication of Potential Targets of XXXT

The bioactive compositions in XXXT were obtained from the Traditional Chinese Medicine System Pharmacology Database (TCMSP, https://tcmspw.com/index.php) [15], which captures the associations between herbs, targets, and diseases. Pharmacokinetic properties were known as the key parameters affecting biological activity, including absorption, distribution, metabolism, and excretion. Drug-likeness (DL ≥ 0.18) and oral availability (OB ≥ 30%) were applied to filter active compounds in XXXT, which were used to establish a database of target genes. The gene names were standardized and annotated through UniProtKB database (https://www.uniprot.org/) [16].

### 2.2. Screening of the Putative Targets of NSCLC and PPI Network Construction

The disease targets related to NSCLC were predicted by integrating multisource databases, containing Therapeutic Target Database (TTD, https://db.idrblab.org/ttd/) [17], GeneCards database (https://www.genecards.org/) [18], DrugBank database (https://go.drugbank.com/) [19], and DisGeNET database (http://www.disgenet.org/) [20]. The bioinformatics server (http://www.bioinformatics.com.cn/) was used to generate Venn diagrams of drug and disease targets, and the targets were identified as potential therapeutic targets of XXXT against NSCLC. The protein-protein interaction (PPI) network was established by searching the STRING Database (https://string-db.org/) [21]. All PPI data were “*Homo sapiens*” with confidence score ≥0.9, and separate nodes were hidden in the network. The obtained PPI information was imported into the Cytoscape software (https://cytoscape.org/).

### 2.3. Gene Ontology and KEGG Pathway Enrichment Analyses

DAVID (https://david.ncifcrf.gov/) [22], an online bioinformatics database, was widely applied to obtain the biological processes, cellular components, molecular functions, and KEGG pathways of drug and disease targets. GO and pathway terms were screened with a false discovery rate (FDR) <0.05 and ranked according to their count, and the top 20 GO/KEGG enrichment was further analyzed.

### 2.4. Network Construction and Topology Analysis

The Cytoscape version 3.7.2 was applied to build the drug-disease-target network. In detail, the XXXT chemical ingredients and putative therapeutic targets against NSCLC were imported into the Cytoscape software, and the size of nodes was arranged according to the ascending order of the number of degree values. The Cytoscape was employed to map the network relationship of bioactive compounds, potential targets, and KEGG pathways for XXXT treatment of NSCLC. Topological properties of the network, including closeness, degree, and betweenness were calculated using the network analyzer tool within Cytoscape 3.7.2 and also were used to seek the main active ingredients and related targets of XXXT against NSCLC.

### 2.5. Screening of Core Targets of XXXT against NSCLC

The Gene Expression Profiling Interactive Analysis (GEPIA, http://gepia.cancer-pku.cn/detail.php) [23] is an interactive RNA-Seq database for gene analysis and cancer type analysis. The targets in compound-target-pathway network were selected in the GEPIA server by “overall survival,” and the cutoff values were chosen by “median” group. *P* < 0.05 and P (HR) < 0.05 were set as the significance threshold in “LUAD” datasets to filter core targets of XXXT against NSCLC.

### 2.6. Cell Lines and Drugs

The lung cancer cell lines A549 and H460 were obtained from the National Collection of Authenticated Cell Cultures (Shanghai, China) and grown in a humidified incubator with 5% CO_2_ at 37°C. All cells were cultured in RPMI 1640 medium supplemented with 10% v/v fetal bovine serum (AusGeneX) and penicillin-streptomycin antibiotic (100 ug/mL). XXXT is composed of Huanglain (Coptidis Rhizoma), Banxia (Arum Ternatum Thunb.), and Gualou (*Trichosanthes* Kirilowii Maxim). The above herbs were purchased from the Huayu Pharmacy Company (Shanghai, China) and XXXT stock was performed as described previously [24]. The vacuum-dried XXXT powder was dissolved in DMSO as stock with a concentration of 500 mg/mL and kept at 4 °C.

### 2.7. Cell Counting Kit-8 Assay Assessed Cell Proliferation

The Cell Counting Kit-8 (CCK8) assay (Sangon Biotech, Shanghai, China) was applied to assess cell viability followed by the manufacturer's instructions. A549 and H460 cells were cultured in a 96-well plate at a density of 2 × 10^3^ cells/well with RPMI-1640 medium with various concentrations (0.0625 mg/ml, 0.125 mg/ml, 0.25 mg/ml, 0.5 mg/ml, 1 mg/ml) of XXXT for 72 h. CCK-8 solution were added directly to the 96-well plate (10 ul/well) and kept in the incubator for 2 h. Optical density (OD) 450 values were measured using a spectrophotometer (Thermo Fisher Scientific, Vantaa, Finland). Inhibition ratio (%) = (1−OD_sample_/OD_control_) × 100%. The IC_50_ value was calculated by using the sigmoidal dose-response function in GraphPad Prism 8.0 software.

### 2.8. Real-Time qPCR Array

RT-qPCR array was performed to analyze gene expression profiles in the light of the manufacturer's instructions (Wcgene Biotech, Shanghai, China). Total RNA was obtained from A549 and H460 cells using trizol reagent (Sangon, Shanghai, China). Reverse transcription reaction into cDNA was performed using miRNA First-Strand cDNA Synthesis kit (Invitrogen; Thermo Fisher Scientific Inc.). SYBR Green I Master Mix kit (Invitrogen; Thermo Fisher Scientific Inc.) was used to perform RT-qPCR on the thermocycler (Applied Biosystems 7300; Thermo Fisher Scientific Inc.). The mRNA expression levels were measured by the 2-ΔΔCq calculation method, and *β*-actin was utilized as an internal control. All primer sets are shown in the supplementary file (Supplementary Table 1).

### 2.9. Western Blot

A549 and H460 cells were treated with XXXT at IC_50_ for 72 h. Total protein was extracted from the cells using ice-cold RIPA lysis buffer, and the bicinchoninic acid protein (BCA) assay kit (ThermoFisher, IL, USA) was used to quantitate protein concentration. Primary antibodies against FOSL2 (cat. #: 15832-1-AP), *α*-tubulin (cat. #: 11224-1-AP), and GAPDH (cat. #: 10494-1-AP) were from Proteintech, and CCNA2 (cat. #: 4656) was from Cell Signaling Technology.

### 2.10. Statistical Analysis

GraphPad Prism 8.0 software was used for statistical analyses and graphs. The data in the *in vitro* were shown as means ± standard deviation (SD). One-way ANOVA followed by Bonferroni test was performed to investigate the differences between groups. In all comparisons, differences were considered statistically significant when *P* < 0.05.

## 3. Results

### 3.1. Active Compounds and Target Screening and PPI Networks Construction

**Figure 2:**
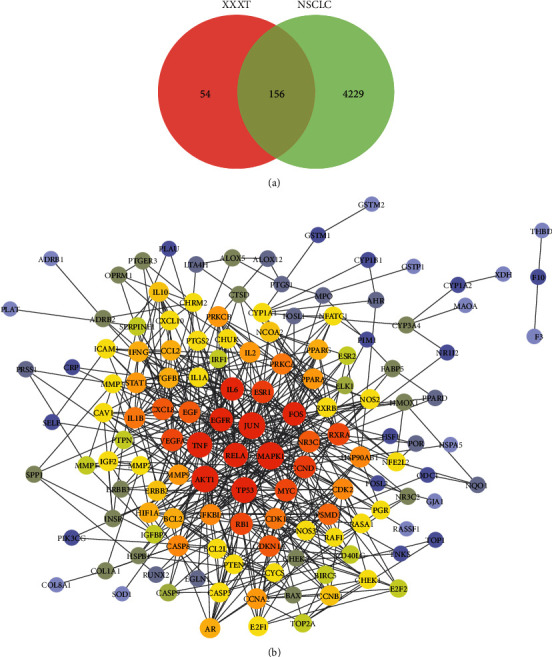
Potential therapeutic targets of XXXT against NSCLC and PPI network construction. (a) The Venn diagram of XXXT and NSCLC intersection targets. (b) PPI network of common targets between XXXT and NSCLC. The greater the degree, the bigger the node.

Through retrieving the TCMSP database and literature, a total of 72 effective components of XXXT were obtained, including 48 species of *Coptidis Rhizoma*, 13 species of *Arum Ternatum Thunb*, and 11 species of *Trichosanthes Kirilowii Maxim*. After ADME screening with the filtering criteria of OB ≥30% and DL ≥0.18, 33 candidate ingredients were considered as active components of XXXT (Table 1) and 210 putative targets were predicted (Supplementary Table 2). The disease targets related to NSCLC were predicted by searching for GeneCards, DrugBank, TTD, and DisGeNET databases, and 4385 targets were obtained after removing duplicated targets (Supplementary Table 3). The 210 targets of XXXT were mapped to 4385 disease targets related to NSCLC. The 156 common targets related to NSCLC and XXXT were obtained and treated as potential targets of XXXT against NSCLC (Figure 2(a), Supplementary Table 4). The protein-protein interaction was constructed by importing 156 common targets related to NSCLC and identified as XXXT into the STRING database. The Cytoscape was used to carry out the visual composition. The network includes 137 round nodes and 573 edges, which represented the interaction between potential protein targets and function (Figure 2(b)).

### 3.2. GO and KEGG Pathway Enrichment Analyses

**Figure 3:**
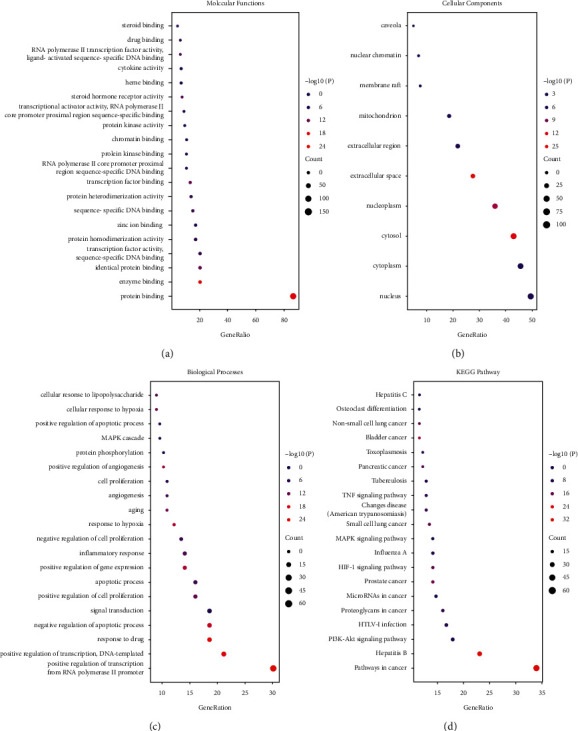
GO and KEGG enrichment analysis of common target for XXXT against NSCLC. Molecular function (a), cellular components (b), and biological process (c) are shown in bubble plots. (d) Top 20 pathway enrichment of XXXT associate with NSCLC. The color scales indicate different thresholds of −log10 *p* values, and the sizes of the dots represent the gene count of each term.

The above 156 targets were imported into the DAVID online server for GO and KEGG pathway analysis. In molecular function, enrichment information includes protein binding, enzyme binding, and chromatin binding (Figure 3(a)). Only 10 terms were selected with FDR <0.05 in cellular components and the GO terms ranked according to their counts, including the nucleus, cytoplasm, cytosol, and nucleoplasm (Figure 3(b)). In biological processes, the targets were enriched in the inflammatory response, apoptotic process, response to hypoxia, angiogenesis, and cell proliferation (Figure 3(c)). KEGG pathway enrichment information indicated that the 156 common targets contributed to 60 pathways (FDR <0.05). Specifically, these pathways were mainly correlated with cancer, such as pathways in cancer, PI3K-AKT, MAPK, HIF-1, and TNF signaling pathway. As shown in Figure 3(d), the top 20 pathways ranked by count were closely related to the etiopathogenesis of NSCLC.

### 3.3. Topological Network Construction of XXXT against NSCLC

**Figure 4:**
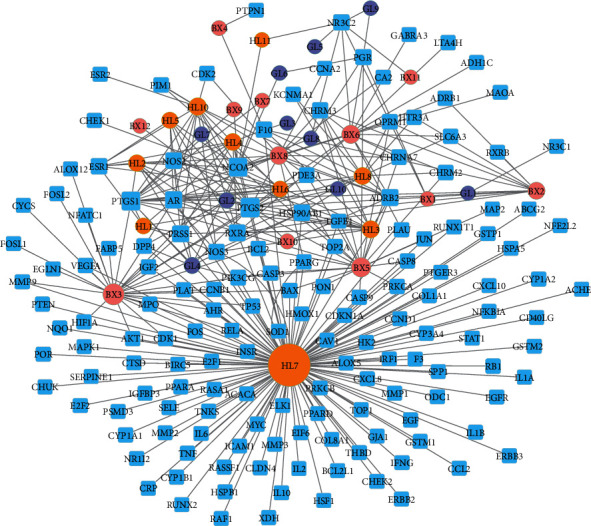
Drug-disease-target network of XXXT against NSCLC. Rectangles represent potential therapeutic targets, while circles represent compounds. Orange represents the active component of Coptidis Rhizoma, pink represents the active component of Arum Ternatum Thunb, and purple represents the active component of *Trichosanthes* Kirilowii Maxim).

**Figure 5:**
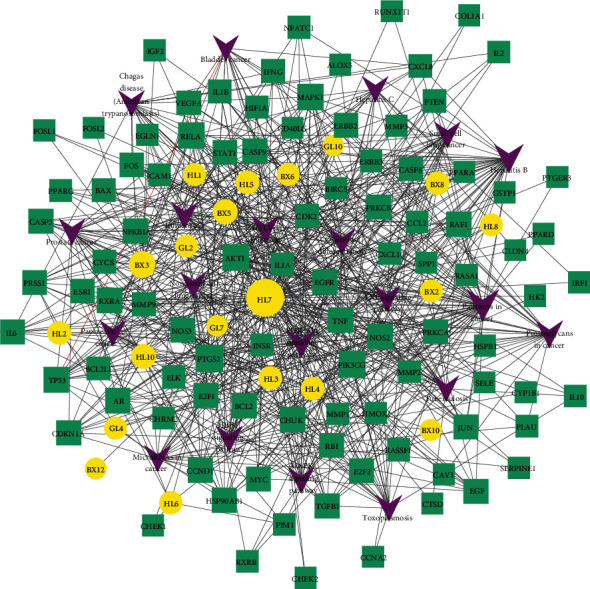
Compound-target-pathway network of XXXT. The yellow ellipse nodes are the active compounds, the green rectangle nodes are the related target genes, and the purple arrow nodes are the pathway.

The drug-disease-target network was constructed which contained 344 edges and 188 nodes (Figure 4). The network revealed the relationship between 32 active ingredients and related 156 common targets (HL10, one of 33 active ingredients, was eliminated due to lack of a common target with NSCLC). An active compound could target different gene targets, and a gene target could be relevant to different active compounds, reflecting the multicomponent and multitarget characteristics of XXXT. The Cytoscape version 3.7.2 was used to establish a drug-target-pathway network of XXXT against NSCLC based on the KEGG pathway enrichment analyses. The network diagram revealed that the 20 active ingredients in XXXT acted on 95 targets and regulated 20 pathways to treat NSCLC. And, the interaction network comprised 135 nodes and 668 edges (Figure 5).

### 3.4. Core Target Screening

**Figure 6:**
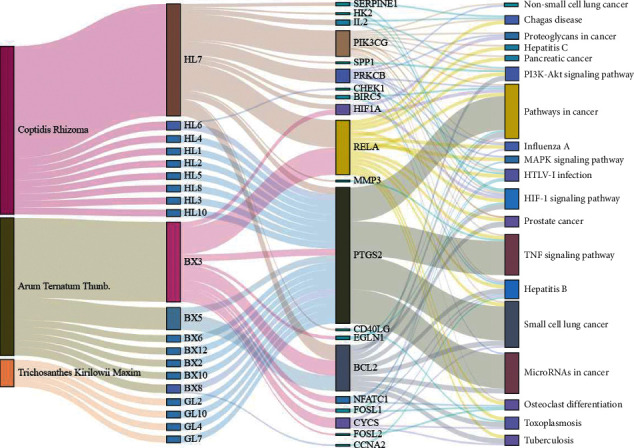
The herb-compound-target-pathway relationship diagram to depict the mechanism of XXXT against NSCLC. The rectangles from left to right represent the herbs, active compounds, core targets, and KEGG pathways of XXXT against NSCLC.

The core targets of XXXT against NSCLC were acquired through retrieving the GEPIA database. Specifically, 95 targets were searched in the GEPIA database, and 20 core targets were filtered through “overall survival” (Table 2). We drew the herb-compound-target-pathway relationship diagram to picture the mechanism of XXXT against NSCLC (Figure 6). The 20 compounds in XXXT regulate 19 pathways through 20 core targets, including PTGS2, PIK3CG, RELA, BCL2, PRKCB, CYCS, HIF1A, NFATC1, IL2, BIRC5, SERPINE1, FOSL1, EGLN1, CHEK1, MMP3, SPP1, CD40LG, HK2, FOSL2, and CCNA2.

### 3.5. The Cytotoxic Effects of XXXT on Lung Cancer Cells

**Figure 7:**
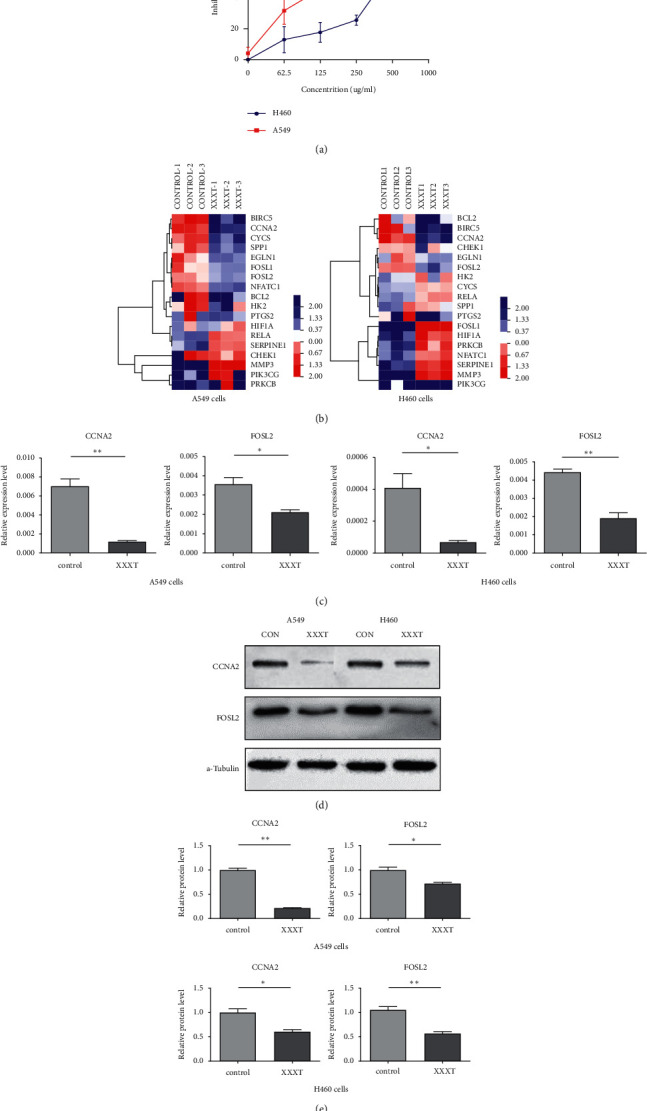
The cytotoxic effects of XXXT on lung cancer cells, RT-qPCR array, and western blot for core targets. (a) The inhibition rates of H460 and A549 cells treated with XXXT (0–1000 *μ*g/mL) for 72 h were determined using CCK8 assay. A549 or H460 treated with XXXT at IC_50_ for 72 h were harvested for analysis (b–e). (b) Heatmap of 20 core targets mRNA expression in the control group and XXXT treatment group. The significant difference of core targets in A549 and H460 cells (c) was observed, including expression of CCNA2 and FOSL2 mRNA. The protein levels of CCNA2 and FOSL2 were determined by western blot (d). Statistical analysis of CCNA2 and FOSL2 proteins expression intensity (e). The above data are presented as mean ± SD for three independent experiments. NS, not significant,  ^*∗*^*P* < 0.05 and  ^*∗∗*^*P* < 0.01 showed significant difference vs. the control group.

To verify the findings from the network pharmacological analysis, we investigated the cytotoxicity of XXXT to decrease the survival of the lung cancer cell lines. Various concentrations of XXXT (0-1 mg/ml) were used to treat two NSCLC cancer cell lines for 72 h, and cell viability was detected by CCK8 assay. As shown in Figure 7(a), XXXT treatment significantly reduced the cell viability of A549 and H460 cells compared with control, and the decrease in cell viability was dependent on the concentrations of XXXT. The IC_50_ values were about 408 ug/ml in H460 cells, and 188 ug/ml in A549 cells.

### 3.6. The Verification of XXXT on mRNA and Protein Levels of Core Targets

To evaluate the effects of XXXT on the mRNA levels of 20 core targets identified from network pharmacology, RT-qPCR array was used after 72 h incubation with IC_50_ dose. XXXT significantly inhibited mRNA expression of CCNA2 and FOSL2 in both H460 and A549 cells. Higher mRNA expression levels of HIF1A, NFATC1, PRKCB, RELA, CYCS, SERPINE1, and FOSL1, and lower mRNA expression levels of CCNA2 and FOSL2 were observed in H460 cells. XXXT treatment tended to decrease the levels of BIRC5 mRNA in H460 cells, though there was no statistically significant difference (Supplementary Table 5). In A549 cells, the mRNA levels of MMP3, RELA, and SERPINE1were upregulated, and the mRNA level of BIRC5, CCNA2, CYCS, FOSL2, NFATC1, and SPP1 was downregulated (Supplementary Table 6). We further detected the protein expressions of CCNA2 and FOSL2, and XXXT also significantly downregulated the protein levels of CCNA2 and FOSL2 in H460 and A549 cells (Figures 7(b)–7(e)).

## 4. Discussion

Due to the complexity and difficulty, the mechanism of the researches on Traditional Chinese Medicine formula has been limited. The network pharmacology is a useful biological information tool for method to analyze the underlying active compounds and give clues for potential mechanism of Traditional Chinese Medicine formula. Firstly, through ADME screening, 33 active compounds were identified from the three herbs in the XXXT, 11 in *Coptidis Rhizoma*, 12 in *Arum Ternatum Thunb*, and 10 in *Trichosanthes Kirilowii Maxim*. Previous studies have confirmed that the most of the 33 compounds of XXXT could induce apoptosis and block the cell cycle in several cancer cells. Berberine (HL4, MOL001454, OB = 36.86%, DL = 0.78) is a small molecule derived from *Coptidis rhizome*, and Berberine could induce mitochondrial apoptosis and G0/*G*1 cell cycle arrest mediated by the PI3K-AKT signaling pathway in the thyroid carcinoma cell lines [25]. Berberine increased the expression of caspase-3 and impaired mitochondrial membrane potential to induce cell apoptosis in human gastric cancer cells [26]. Another study has revealed that Berberine induced cell cycle arrest and inhibited migration and invasion of lung cancer cells [27]. Baicalein (BX3, MOL002714, OB = 33.52%, DL = 0.21), a flavone present in another ingredient from *Arum Ternatum Thunb* induced mitochondrial-dependent apoptosis, and blocked S-phase cell cycle in human cisplatin-resistant pancreatic carcinoma cell line CAPAN-2 [28]. Baicalin was known as the important component in several herbs, unleashing significant antiproliferative action in a range of cancer cell lines by modulating apoptosis and cell cycle [29]. The natural flavonoid diosmetin (GL2, MOL002881, OB = 31.14%, DL = 0.27) present in *Trichosanthes Kirilowii Maxim* exerts anticancer effects through apoptosis induction and G2/M cell cycle arrest in HepG2 cells [30]. Diosmetin also has proapoptotic activities against breast cancer cells via activating the mitochondria-mediated apoptotic pathway and inducing cell cycle arrest [31]. As mentioned above, 33 potential bioactive compounds in XXXT against NSCLC were screened out by TCMSP-based systems pharmacology.

Based on the above active compounds in XXXT, the further analysis of related core targets was conducted and confirmed. As XXXT has been reported to be effective on lung cancer, the potential targets of XXXT based on the above active compounds were overlapped with NSCLC-related targets. Then, combined with the KEGG and survival analysis results, 20 of candidate targets were selected and proceed to be proved. It was confirmed that XXXT could inhibit the mRNA and protein levels of CCNA2 and FOSL2 *in vitro*. CCNA2 is a regulator of the G1/S and G2/M transition, which targeted by coniferin (BX8). CCNA2 is highly expressed in several cancer cells and contributes to epithelial-mesenchymal transition through dual activation of WNT and PLC pathways [32, 33]. FOSL2 targeted by baicalein, is a regulator of cell proliferation, differentiation, and transformation. Several studies have reported that the phosphorylation and upregulation of FOSL2 enhance tumor growth and invasion in A549 cells through miR-638 [34–37]. In A549 cells, the mRNA level of BIRC5 was downregulated, and XXXT treatment tended to decrease the levels of BIRC5 mRNA in H460 cells, though there was no statistically significant difference. BIRC5, an inhibitor of the apoptotic gene family, was targeted by quercetin (HL7). The recent study noted that the high-expression BIRC5 was correlated with low overall survival in lung adenocarcinoma patients, and the overexpression of BIRC5 is a risk factor for a worse prognosis [38]. Apoptosis repeat protein domain was inhabited by BIRC5 to militate against apoptosis inhibition [39], and siBIRC5 could overcome the antiapoptosis protection of cisplatin-resistant cells [40]. As a necessary protein of CPC, BIRC5 is also required for the mitotic exit and plays a major role in the cell cycle [41].

**Figure 8:**
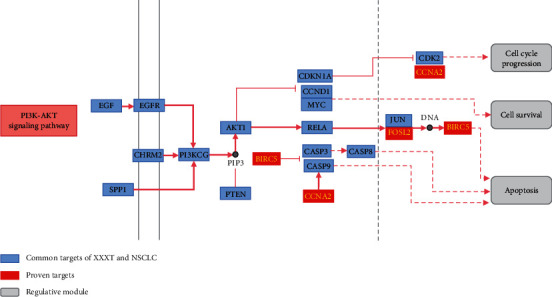
Distribution of targets of XXXT in the PI3K-Akt signaling pathway. Arrow shows activation effect; T-shaped arrow shows inhibition effect, and dotted arrow represents indirect activation effect or inhibition effect.

Finally, the potential mechanisms of XXXT on NSCLC treatment were mapped by the herb-compound-target-pathway relationship diagram. Network pharmacological analysis uncovered that the 20 compounds XXXT ameliorate NSCLC therapy by regulating 19 pathways, such as PI3K-AKT, TNF, and MAPK signaling pathways. Further experiments *in vitro* revealed that the potential targets of XXXT for NSCLC treatment are closely related to the PI3K-AKT pathway. As a master regulator, PI3K-AKT signaling pathway influences cancer cell proliferation, metastasis, and metabolism [42–44]. The suppression of PI3K-AKT signaling pathway is an attractive strategy for NSCLC management via promoting apoptosis and inhibiting cell growth and invasion [45]. Apoptosis and cell cycle are two vital biological processes, by which the PI3K signaling pathway regulates tumor cell proliferation and viability [46–48]. Apoptosis and cell cycle arrest can be induced by several physiological regulations and chemical stimulation, the induction of which is recognized as effective steps for the treatment of cancer [49–51]. Therefore, we suggest that XXXT can inhibit the PI3K-AKT signaling pathway in NSCLC by targeting CCNA2, FOSL2, and BIRC5 (Figure 8).

## 5. Conclusion

For the study, network pharmacology was employed to investigate the drug-component-target-disease interaction of XXXT in the treatment of NSCLC. XXXT may induce lung cancer cell apoptosis and cell cycle arrest by regulating the PI3K-AKT signaling pathway. The results from this study provide insight into anticancer action and potential mechanism of XXXT against NSCLC and lay the foundation for further development of XXXT or its active ingredients as the complementary therapy for NSCLC treatment.

## Figures and Tables

**Table 1:** Compounds information sheet.

Herb	Symbol	MolID	Compounds	Structure	OB	DL
*Coptidis rhizoma*	HL1	MOL001458	Coptisine	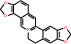	30.67	0.86
	HL2	MOL002894	Berberrubine	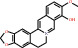	35.74	0.73
	HL3	MOL002904	Berlambine	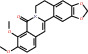	36.68	0.82
	HL4	MOL001454	Berberine	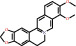	36.86	0.78
	HL5	MOL002897	Epiberberine	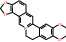	43.09	0.78
	HL6	MOL002668	Worenine	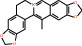	45.83	0.87
	HL7	MOL000098	Quercetin	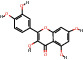	46.43	0.28
	HL8	MOL002903	(R)-canadine	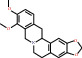	55.37	0.77
	HL9	MOL000622	Magnograndiolide	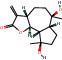	63.71	0.19
	HL10	MOL000785	Palmatine	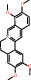	64.60	0.65
	HL11	MOL002907	Corchoroside- A_qt	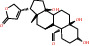	104.95	0.78
*Arum ternatum thunb*	BX1	MOL001755	24-Ethylcholest-4-en-3-one	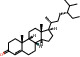	36.08	0.76
	BX2	MOL002670	Cavidine	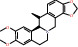	35.64	0.81
	BX3	MOL002714	Baicalein	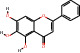	33.52	0.21
	BX4	MOL002776	Baicalin	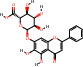	40.12	0.75
	BX5	MOL000358	Beta-sitosterol	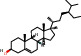	36.91	0.75
	BX6	MOL000449	Stigmasterol	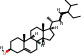	43.83	0.76
	BX7	MOL005030	Gondoic acid	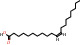	30.70	0.20
	BX8	MOL000519	Coniferin	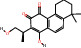	31.11	0.32
	BX9	MOL006936	10,13-Eicosadienoic	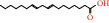	39.99	0.20
	BX10	MOL006957	(3S,6S)-3-(benzyl)-6-(4-hydroxybenzyl)piperazine-2,5-quinone	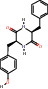	46.89	0.27
	BX11	MOL003578	Cycloartenol	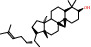	38.69	0.78
	BX12	MOL006967	Beta-D-Ribofuranoside, xanthine-9	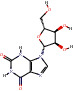	44.72	0.21

*Trichosanthes kirilowii maxim*	GL1	MOL007171	5-Dehydrokarounidiol	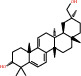	30.23	0.77
	GL2	MOL002881	Diosmetin	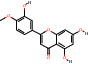	31.14	0.27
	GL3	MOL007180	Vitamin-e	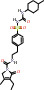	32.29	0.70
	GL4	MOL005530	Hydroxygenkwanin	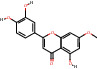	36.47	0.27
	GL5	MOL007172	7-Oxo-dihydrokaro-unidiol	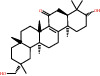	36.85	0.75
	GL6	MOL006756	Schottenol	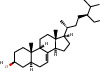	37.42	0.75
	GL7	MOL001494	Mandenol	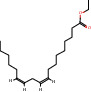	42.00	0.19
	GL8	MOL004355	Spinasterol	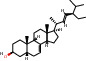	42.98	0.76
	GL9	MOL007165	10*α*-Cucurbita-5,24-diene-3*β*-ol	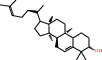	44.02	0.74
	GL10	MOL007179	Linolenic acid ethyl ester	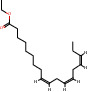	46.10	0.20

**Table 2:** Information on 20 core targets.

Target name	Betweenness centrality	Closeness centrality	Degree	Overall survival
PTGS2	0.11098661	0.51538462	24	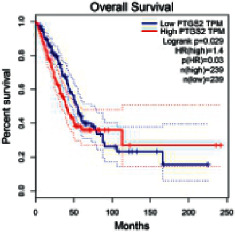
PIK3CG	0.02201518	0.49264706	19	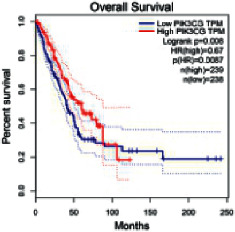
RELA	0.01545686	0.48550725	18	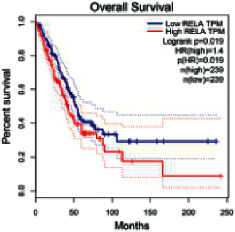
BCL2	0.00792956	0.46527778	12	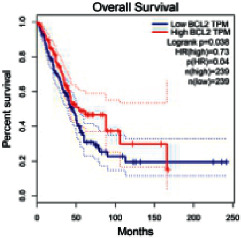
PRKCB	0.00414970	0.44966443	9	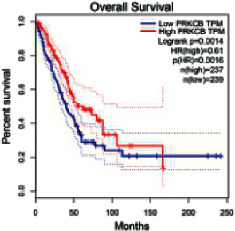
CYCS	0.00182322	0.39181287	7	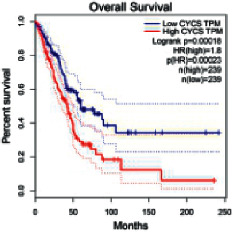
HIF1A	0.00161744	0.4379085	5	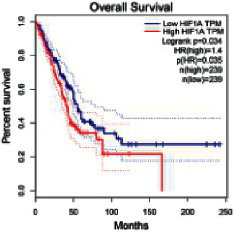
NFATC1	0.00092885	0.36216216	5	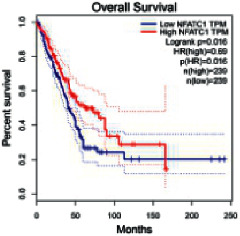
IL2	0.00106660	0.42675159	4	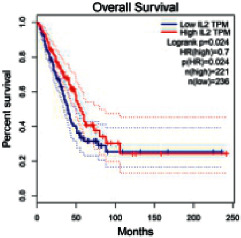
BIRC5	0.00043119	0.42675159	3	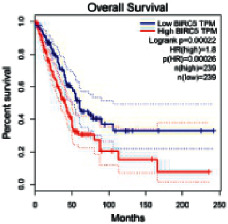
SERPINE1	0.00046992	0.41358025	3	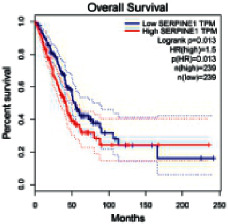
FOSL1	0.00031530	0.33333333	3	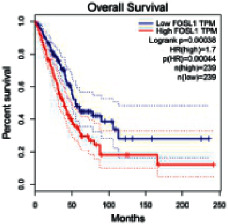
EGLN1	0.00036757	0.36813187	3	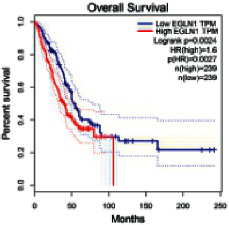
CHEK1	0.00063767	0.31018519	2	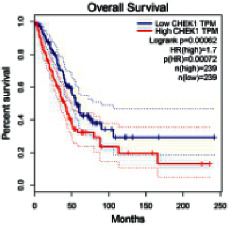
MMP3	0.00016505	0.40606061	2	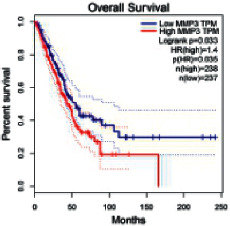
SPP1	0.00019974	0.41104294	2	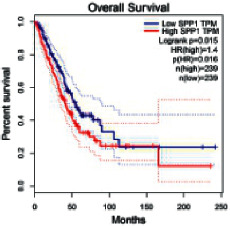
CD40LG	0.00022360	0.41104294	2	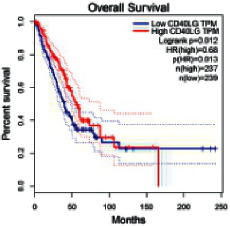
HK2	0.00019953	0.41104294	2	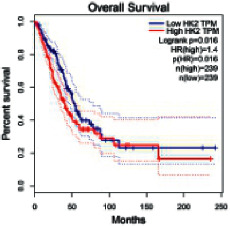
FOSL2	0.00009376	0.31162791	2	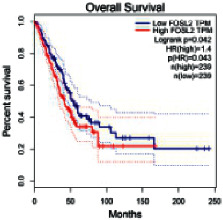
CCNA2	0.00048920	0.32524272	2	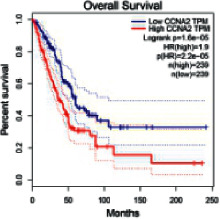

## Data Availability

The datasets used and analyzed during the current study are available from the corresponding author on reasonable request.

## Abbreviations

ADME: Absorption, distribution, metabolism, excretion
AP-1: Activator protein-1
BCA: Bicinchoninic acid protein
BCL2: B-cell lymphoma-2
BIRC5: Baculoviral IAP repeat containing 5
BP: Biological processes
CC: Cellular components
CCNA2: Cyclin A2
CD40LG: CD40 ligand
CHEK1: Checkpoint kinase 1
CPC: Chromosomal passenger complex
CYCS: Cytochrome C: somatic
DL: Drug-likeness
DMSO: Dimethyl sulfoxide
EGLN1: Egl-9 family hypoxia inducible factor
FDR: False discovery rate
FOSL1: FOS-like 1
FOSL2: FOS-like 2
HIF-1: Hypoxia inducible factor-1
HIF1A: Hypoxia inducible factor 1 subunit alpha
HK2: Hexokinase 2
IL2: Interleukin 2
KEGG: Kyoto Encyclopedia of Genes and Genomes
LUAD: Lung adenocarcinoma
MF: Molecular functions
MMP3: Matrix metallopeptidase 3
NFATC1: Nuclear factor of activated T cells 1
NSCLC: Non-small cell lung cancer
OB: Oral availability
OD: Optical density
PIK3CG: Phosphatidylinositol-4,5-bisphosphate 3-kinase catalytic subunit gamma
PI3K: Phosphatidylinositide 3-threonine-protein kinase
PRKCB: Protein kinase *c* beta
PTGS2: Prostaglandin-endoperoxide synthase 2
RELA: v-rel reticuloendotheliosis viral oncogene homolog A
SERPINE1: Serpin family *e* member 1
SPP1: Secreted phosphoprotein 1
TCM: Traditional Chinese Medicine
TNF: Tumor necrosis factor
XXXT: Xiaoxianxiong Tang.

## Supplementary Materials

Supplementary Materials All primer sets in the RT-qPCR array are shown in the Table 1. Table 2 indicates the targets in XXXT. Table 3 indicates the targets related to NSCLC. Table 4 indicates common targets of NSCLC and XXXT. Table 5 indicates the result of the RT-qPCR array in H460 cells. Table 5 indicates the result of the RT-qPCR array in A549 cells.
