# Supplementary material for: Anticancer Action of Xiaoxianxiong Tang in Non-Small Cell Lung Cancer by Pharmacological Analysis and Experimental Validation
Source: Evid Based Complement Alternat Med. 2021 Dec 13;2021:9930082. doi: 10.1155/2021/9930082 (PMC8687818; doi:10.1155/2021/9930082)
Supplement: Supplementary Materials — All primer sets in the RT-qPCR array are shown in the Table 1. Table 2 indicates the targets in XXXT. Table 3 indicates the targets related to NSCLC. Table 4 indicates common targets of NSCLC and XXXT. Table 5 indicates the result of the RT-qPCR array in H460 cells. Table 5 indicates the result of the RT-qPCR array in A549 cells. [file 9930082.f1.zip › 9930082.f1/Supplementary table 1-primers.pdf]

gene	F	R	product
BCL2	ATTGATGGGATCGTTGCCTTAT	TCCAATTCCTTTCGGATCTTTA	170
BIRC5	TGGGAAGGGTTGTGAATGAG	CAGTTTGGCTTGCTGGTCTC	137
CCNA2	TGGAAAGCAAACAGTAAACAGCC	GGGCATCTTCACGCTCTATTT	109
CD40LG	ACATACAACCAAACTTCTCCCCG	GCAAAAAGTGCTGACCCAATCA	119
CHEK1	ATATGAAGCGTGCCGTAGACT	TGCCTATGTCTGGCTCTATTCTG	183
CYCS	TGTGCCAGCGACTAAAAAGA	CCTCCCTTTTCAACGGTGT	103
EGLN1	CTCGTCCAAGGACATCCGAG	TCATGAGCAGCCCAATGGTT	83
FOSL1	CAGGCGGAGACTGACAAACTG	TCCTTCCGGGATTTTGCAGAT	132
FOSL2	GCCAGCAGAAATTCCGGGTA	ATGGGTTGGACATGGAGGTG	132
HIF1A	GAACGTCGAAAAGAAAAGTCTCG	CCTTATCAAGATGCGAACTCACA	124
HK2	TGCCACCAGACTAAACTAGACG	CCCGTGCCCACAATGAGAC	227
IL2	CCCAAGAAGGCCACAGAACT	GCACTTCCTCCAGAGGTTTGA	70
MMP3	TGAAATTGGCCACTCCCTGG	GGAACCGAGTCAGGTCTGTG	95
NFATC1	CACCGCATCACAGGGAAGAC	GCACAGTCAATGACGGCTC	119
PIK3CG	AACACCGACCTCACAGTTTT	CTCAAGCCACACATTCCACA	120
PRKCB	GGATTGGGATTTGACCAGCAG	TGGCACAGGCACATTGAAGT	139
PTGS2	TAAGTGCGATTGTACCCGGAC	TTTGTAGCCATAGTCAGCATTGT	250
RELA	CCCACGAGCTTGTAGGAAAGG	GGATTCCCAGGTTCTGGAAAC	96
SERPINE1	ACCGCAACGTGGTTTTCTCA	TTGAATCCCATAGCTGCTTGAAT	109
SPP1	GAAGTTTCGCAGACCTGACAT	GTATGCACCATTCAACTCCTCG	91
GAPDH	CAATGACCCCTTCATTGACC	GACAAGCTTCCCGTTCTCAG	106
