# Supplementary material for: Anticancer Action of Xiaoxianxiong Tang in Non-Small Cell Lung Cancer by Pharmacological Analysis and Experimental Validation
Source: Evid Based Complement Alternat Med. 2021 Dec 13;2021:9930082. doi: 10.1155/2021/9930082 (PMC8687818; doi:10.1155/2021/9930082)
Supplement: Supplementary Materials — All primer sets in the RT-qPCR array are shown in the Table 1. Table 2 indicates the targets in XXXT. Table 3 indicates the targets related to NSCLC. Table 4 indicates common targets of NSCLC and XXXT. Table 5 indicates the result of the RT-qPCR array in H460 cells. Table 5 indicates the result of the RT-qPCR array in A549 cells. [file 9930082.f1.zip › 9930082.f1/Supplementary table 2-Targets in XXXT.pdf]

Herb Symbol	Gene_Full_Name	Gene Symbol
HL1	"Nitric oxide synthase, inducible"	NOS2
HL1	Prostaglandin G/H synthase 1	PTGS1
HL1	Potassium voltage-gated channel subfamily H member 2	KCNH2
HL1	Estrogen receptor	ESR1
HL1	Androgen receptor	AR
HL1	Sodium channel protein type 5 subunit alpha	SCN5A
HL1	Prostaglandin G/H synthase 2	PTGS2
HL1	"Nitric-oxide synthase, endothelial"	NOS3
HL1	Trypsin-1	PRSS1
HL2	"Nitric oxide synthase, inducible"	NOS2
HL2	Prostaglandin G/H synthase 1	PTGS1
HL2	Potassium voltage-gated channel subfamily H member 2	KCNH2
HL2	Estrogen receptor	ESR1
HL2	Androgen receptor	AR
HL2	Sodium channel protein type 5 subunit alpha	SCN5A
HL2	Prostaglandin G/H synthase 2	PTGS2
HL2	"Nitric-oxide synthase, endothelial"	NOS3
HL2	Retinoic acid receptor RXR-alpha	RXRA
HL2	Trypsin-1	PRSS1
HL2	Nuclear receptor coactivator 2	NCOA2
HL3	"Nitric oxide synthase, inducible"	NOS2

HL3 Prostaglandin G/H synthase 1 PTGS1

HL3 Muscarinic acetylcholine receptor M3 CHRM3

HL3 Potassium voltage-gated channel subfamily H member 2 KCNH2

HL3 Androgen receptor AR

HL3 Sodium channel protein type 5 subunit alpha SCN5A

HL3 Coagulation factor Xa F10

HL3 Prostaglandin G/H synthase 2 PTGS2

HL3 "Nitric-oxide synthase, endothelial" NOS3

HL3 Coagulation factor VII F7

HL3 Retinoic acid receptor RXR-alphaRXRA

HL3 Alpha-1B adrenergic receptor ADRA1B

HL3 Beta-2 adrenergic receptor ADRB2

HL3 Alpha-1D adrenergic receptor ADRA1D

HL3 Heat shock protein HSP 90 HSP90AB1

HL3 Trypsin-1 PRSS1

HL3 Nuclear receptor coactivator 2 NCOA2

HL3 Calcium-activated potassium channel subunit alpha 1 KCNMA1

HL4 "Nitric oxide synthase, inducible" NOS2

HL4 Prostaglandin G/H synthase 1 PTGS1

HL4 Potassium voltage-gated channel subfamily H member 2 KCNH2

HL4 Estrogen receptor ESR1

HL4 Androgen receptor AR

HL4 Sodium channel protein type 5 subunit alpha SCN5A

HL4 Coagulation factor Xa F10

HL4 Prostaglandin G/H synthase 2 PTGS2

HL4 Retinoic acid receptor RXR-alphaRXRA

HL4 Beta-2 adrenergic receptor ADRB2

HL4 Trypsin-1 PRSS1

HL4 Nuclear receptor coactivator 2 NCOA2

HL4 "cAMP and cAMP-inhibited cGMP 3',5'-cyclic phosphodiesterase 10A" PDE10A

HL5 "Nitric oxide synthase, inducible" NOS2

HL5 Potassium voltage-gated channel subfamily H member 2 KCNH2

HL5 Estrogen receptor ESR1

HL5 Androgen receptor AR

HL5 Prostaglandin G/H synthase 2 PTGS2

HL5 "Nitric-oxide synthase, endothelial" NOS3

HL5 Retinoic acid receptor RXR-alphaRXRA

HL5 Trypsin-1 PRSS1

HL5 Nuclear receptor coactivator 2 NCOA2

HL5 "cAMP and cAMP-inhibited cGMP 3',5'-cyclic phosphodiesterase 10A" PDE10A

HL6 "Nitric oxide synthase, inducible" NOS2

HL6 Prostaglandin G/H synthase 1 PTGS1

HL6 Estrogen receptor ESR1

HL6 Androgen receptor AR

HL6 Prostaglandin G/H synthase 2 PTGS2

HL6 Serine/threonine-protein kinase Chk1 CHEK1

HL6 Proto-oncogene serine/threonine-protein kinase Pim-1 PIM1

HL7 Prostaglandin G/H synthase 1 PTGS1

HL7 Androgen receptor AR

HL7 Peroxisome proliferator activated receptor gamma PPARG

HL7 Prostaglandin G/H synthase 2 PTGS2

HL7 Heat shock protein HSP 90 HSP90AB1

HL7 "Phosphatidylinositol-4,5-bisphosphate 3-kinase catalytic subunit, gamma isoform" PIK3CG

HL7 Nuclear receptor coactivator 2 NCOA2

HL7 Dipeptidyl peptidase IV DPP4

HL7 Trypsin-1 PRSS1

HL7 DNA topoisomerase II TOP2

HL7 Potassium voltage-gated channel subfamily H member 2 KCNH2

HL7 Sodium channel protein type 5 subunit alpha SCN5A

HL7 Coagulation factor Xa F10

HL7 Beta-2 adrenergic receptor ADRB2

HL7 Stromelysin-1 MMP3

HL7 Coagulation factor VII F7

HL7 "Nitric-oxide synthase, endothelial" NOS3

HL7 Retinoic acid receptor RXR-alphaRXRA

HL7 Acetylcholinesterase ACHE

HL7 Gamma-aminobutyric acid receptor subunit alpha-1 GABRA1

HL7 Amine oxidase [flavin-containing] B MAOB

HL7 Transcription factor p65 RELA

HL7 Epidermal growth factor receptorEGFR

HL7 RAC-alpha serine/threonine-protein kinase AKT1

HL7 Vascular endothelial growth factor A VEGFA

HL7 G1/S-specific cyclin-D1 CCND1

HL7 Apoptosis regulator Bcl-2 BCL2

HL7 Bcl-2-like protein 1 BCL2L1

HL7 Proto-oncogene c-Fos FOS

HL7 Cyclin-dependent kinase inhibitor 1 CDKN1A

HL7 Eukaryotic translation initiation factor 6 EIF6

HL7 Apoptosis regulator BAX BAX

HL7 Caspase-9 CASP9

HL7 Urokinase-type plasminogen activator PLAU

HL7 72 kDa type IV collagenase MMP2

HL7 Matrix metalloproteinase-9 MMP9

HL7 Mitogen-activated protein kinase 1 MAPK1

HL7 Interleukin-10 IL10

HL7 Pro-epidermal growth factor EGF

HL7 Retinoblastoma-associated protein RB1

HL7 Tumor necrosis factor TNF

HL7 Transcription factor AP-1 JUN

HL7 Interleukin-6 IL6

HL7 Activator of 90 kDa heat shock protein ATPase homolog 1 AHSA1

HL7 Caspase-3 CASP3

HL7 Cellular tumor antigen p53 TP53

HL7 ETS domain-containing protein Elk-1 ELK1

HL7 NF-kappa-B inhibitor alpha NFKBIA

HL7 NADPH--cytochrome P450 reductasePOR

HL7 Ornithine decarboxylase ODC1

HL7 Xanthine dehydrogenase/oxidase XDH

HL7 Caspase-8 CASP8

HL7 DNA topoisomerase 1 TOP1

HL7 RAF proto-oncogene serine/threonine-protein kinase RAF1

HL7 Superoxide dismutase [Cu-Zn] SOD1

HL7 Protein kinase C alpha type PRKCA

HL7 Interstitial collagenase MMP1

HL7 Hypoxia-inducible factor 1-alphaHIF1A

HL7 Signal transducer and activator of transcription 1-alpha/beta STAT1

HL7 Protein CBFA2T1 RUNX1T1

HL7 Cell division control protein 2 homolog CDK1

HL7 78 kDa glucose-regulated proteinHSPA5

HL7 Receptor tyrosine-protein kinase erbB-2 ERBB2

HL7 Peroxisome proliferator activated receptor gamma PPARG

HL7 Acetyl-CoA carboxylase 1 ACACA

HL7 Heme oxygenase 1 HMOX1

HL7 Cytochrome P450 3A4 CYP3A4

HL7 Cytochrome P450 1A2 CYP1A2

HL7 Caveolin-1 CAV1

HL7 Myc proto-oncogene protein MYC

HL7 Tissue factor F3

HL7 Gap junction alpha-1 protein GJA1

HL7 Cytochrome P450 1A1 CYP1A1

HL7 Intercellular adhesion molecule 1 ICAM1

HL7 Interleukin-1 beta IL1B

HL7 C-C motif chemokine 2 CCL2

HL7 E-selectin SELE

HL7 Vascular cell adhesion protein 1VCAM1

HL7 Prostaglandin E2 receptor EP3 subtype PTGER3

HL7 Interleukin-8 CXCL8

HL7 Protein kinase C beta type PRKCB

HL7 Baculoviral IAP repeat-containing protein 5 BIRC5

HL7 Dual oxidase 2 DUOX2

HL7 "Nitric-oxide synthase, endothelial" NOS3

HL7 Heat shock protein beta-1 HSPB1

HL7 Transforming growth factor beta-1 TGFB1

HL7 Estrogen sulfotransferase SULT1E1

HL7 "Maltase-glucoamylase, intestinal" MGAM

HL7 Interleukin-2 IL2

HL7 Nuclear receptor subfamily 1 group I member 2 NR1I2

HL7 Cytochrome P450 1B1 CYP1B1

HL7 G2/mitotic-specific cyclin-B1 CCNB1

HL7 Tissue-type plasminogen activator PLAT

HL7 Thrombomodulin THBD

HL7 Plasminogen activator inhibitor 1 SERPINE1

HL7 Collagen alpha-1(I) chain COL1A1

HL7 Interferon gamma IFNG

HL7 Arachidonate 5-lipoxygenase ALOX5

HL7 "Phosphatidylinositol-3,4,5-trisphosphate 3-phosphatase and dual-specificity protein phosphatase PTEN" PTEN

HL7 Interleukin-1 alpha IL1A

HL7 Myeloperoxidase MPO

HL7 DNA topoisomerase 2-alpha TOP2A

HL7 Neutrophil cytosol factor 1 NCF1

HL7 ATP-binding cassette sub-family G member 2 ABCG2

HL7 Hyaluronan synthase 2 HAS2

HL7 Glutathione S-transferase P GSTP1

HL7 Nuclear factor erythroid 2-related factor 2 NFE2L2

HL7 NAD(P)H dehydrogenase [quinone] 1 NQO1

HL7 Poly [ADP-ribose] polymerase 1 TNKS

HL7 Aryl hydrocarbon receptor AHR

HL7 26S proteasome non-ATPase regulatory subunit 3 PSMD3

HL7 "Solute carrier family 2, facilitated glucose transporter member 4" SLC2A4

HL7 Collagen alpha-1(III) chain COL8A1

HL7 C-X-C motif chemokine 11 CXCL11

HL7 C-X-C motif chemokine 2 CXCL2

HL7 DDB1- and CUL4-associated factor 5 DCAF5

HL7 Nuclear receptor subfamily 1 group I member 3 NR1I3

HL7 Serine/threonine-protein kinase Chk2 CHEK2

HL7 Insulin receptor INSR

HL7 Claudin-4 CLDN4

HL7 Peroxisome proliferator-activated receptor alpha PPARA

HL7 Peroxisome proliferator-activated receptor delta PPARD

HL7 Heat shock factor protein 1 HSF1

HL7 C-reactive protein CRP

HL7 C-X-C motif chemokine 10 CXCL10

HL7 Inhibitor of nuclear factor kappa-B kinase subunit alpha CHUK

HL7 Osteopontin SPP1

HL7 Runt-related transcription factor 2 RUNX2

HL7 Ras association domain-containing protein 1 RASSF1

HL7 Transcription factor E2F1 E2F1

HL7 Transcription factor E2F2 E2F2

HL7 Prostatic acid phosphatase ACP3

HL7 Cathepsin D CTSD

HL7 Insulin-like growth factor-binding protein 3 IGFBP3

HL7 Insulin-like growth factor II IGF2

HL7 CD40 ligand CD40LG

HL7 Interferon regulatory factor 1 IRF1

HL7 Receptor tyrosine-protein kinase erbB-3 ERBB3

HL7 Serum paraoxonase/arylesterase 1 PON1

HL7 Type I iodothyronine deiodinase DIO1

HL7 Procollagen C-endopeptidase enhancer 1 PCOLCE

HL7 Puromycin-sensitive aminopeptidase NPEPPS

HL7 Hexokinase-2 HK2

HL7 Homeobox protein Nkx-3.1 NKX3-1

HL7 Ras GTPase-activating protein 1 RASA1

HL7 Glutathione S-transferase Mu 1 GSTM1

HL7 Glutathione S-transferase Mu 2 GSTM2

HL8 Prostaglandin G/H synthase 1 PTGS1

HL8 Muscarinic acetylcholine receptor M3 CHRM3

HL8 Potassium voltage-gated channel subfamily H member 2 KCNH2

HL8 Muscarinic acetylcholine receptor M1 CHRM1

HL8 Sodium channel protein type 5 subunit alpha SCN5A

HL8 Coagulation factor Xa F10

HL8 Muscarinic acetylcholine receptor M5 CHRM5

HL8 Prostaglandin G/H synthase 2 PTGS2

HL8 5-hydroxytryptamine receptor 3A HTR3A

HL8 Alpha-2C adrenergic receptor ADRA2C

HL8 Muscarinic acetylcholine receptor M4 CHRM4

HL8 Delta-type opioid receptor OPRD1

HL8 5-hydroxytryptamine 2A receptor HTR2A

HL8 5-hydroxytryptamine 2C receptor HTR2C

HL8 Alpha-1B adrenergic receptor ADRA1B

HL8 Sodium-dependent dopamine transporter SLC6A3

HL8 Beta-2 adrenergic receptor ADRB2

HL8 Alpha-1D adrenergic receptor ADRA1D

HL8 Sodium-dependent serotonin transporter SLC6A4

HL8 Mu-type opioid receptor OPRM1

HL8 Heat shock protein HSP 90 HSP90AB1

HL8 "cAMP and cAMP-inhibited cGMP 3',5'-cyclic phosphodiesterase 10A" PDE10A

HL8 Dopamine D1 receptor DRD1

HL8 D(1B) dopamine receptor DRD5

HL8 Retinoic acid receptor RXR-alphaRXRA

HL8 Sodium-dependent noradrenaline transporter SLC6A2

HL8 Alpha-1A adrenergic receptor ADRA1A

HL8 Muscarinic acetylcholine receptor M2 CHRM2

HL8 Calcium-activated potassium channel subunit alpha 1 KCNMA1

HL9 Gamma-aminobutyric-acid receptor alpha-2 subunit GABRA2

HL9 Gamma-aminobutyric acid receptor subunit alpha-1 GABRA1

HL9 Glutamate receptor 2 GRIA2

HL9 Gamma-aminobutyric-acid receptor subunit alpha-6 GABRA6

HL10 "Nitric oxide synthase, inducible" NOS2

HL10 Prostaglandin G/H synthase 1 PTGS1

HL10 Potassium voltage-gated channel subfamily H member 2 KCNH2

HL10 Estrogen receptor ESR1

HL10 Androgen receptor AR

HL10 Sodium channel protein type 5 subunit alpha SCN5A

HL10 Prostaglandin G/H synthase 2 PTGS2

HL10 "Nitric-oxide synthase, endothelial" NOS3

HL10 Retinoic acid receptor RXR-alphaRXRA

HL10 Beta-2 adrenergic receptor ADRB2

HL10 Estrogen receptor beta ESR2

HL10 Heat shock protein HSP 90 HSP90AB1

HL10 Trypsin-1 PRSS1

HL10 Proto-oncogene serine/threonine-protein kinase Pim-1 PIM1

HL10 Nuclear receptor coactivator 2 NCOA2

HL10 Cell division protein kinase 2 CDK2

HL10 Coagulation factor VII F7

HL11 Mineralocorticoid receptor NR3C2

HL11 Nuclear receptor coactivator 2 NCOA2

BX1 Progesterone receptor PGR

BX1 Mineralocorticoid receptor NR3C2

BX2 Prostaglandin G/H synthase 1 PTGS1

BX2 Muscarinic acetylcholine receptor M3 CHRM3

BX2 Potassium voltage-gated channel subfamily H member 2 KCNH2

BX2 Muscarinic acetylcholine receptor M1 CHRM1

BX2 Beta-1 adrenergic receptor ADRB1

BX2 Sodium channel protein type 5 subunit alpha SCN5A

BX2 Coagulation factor Xa F10

BX2 Muscarinic acetylcholine receptor M5 CHRM5

BX2 Prostaglandin G/H synthase 2 PTGS2

BX2 5-hydroxytryptamine receptor 3A HTR3A

BX2 Alpha-2C adrenergic receptor ADRA2C

BX2 Muscarinic acetylcholine receptor M4 CHRM4

BX2 Retinoic acid receptor RXR-alphaRXRA

BX2 Delta-type opioid receptor OPRD1

BX2 5-hydroxytryptamine 2A receptor HTR2A

BX2 5-hydroxytryptamine 2C receptor HTR2C

BX2 Alpha-1B adrenergic receptor ADRA1B

BX2 Beta-2 adrenergic receptor ADRB2

BX2 Alpha-1D adrenergic receptor ADRA1D

BX2 DNA topoisomerase 2-alpha TOP2A

BX2 Mu-type opioid receptor OPRM1

BX2 Heat shock protein HSP 90 HSP90AB1

BX2 Retinoic acid receptor RXR-beta RXRB

BX2 Dopamine D1 receptor DRD1

BX2 Sodium-dependent serotonin transporter SLC6A4

BX2 Coagulation factor VII F7

BX2 "cAMP and cAMP-inhibited cGMP 3',5'-cyclic phosphodiesterase 10A" PDE10A

BX3 Prostaglandin G/H synthase 1 PTGS1

BX3 Androgen receptor AR

BX3 Prostaglandin G/H synthase 2 PTGS2

BX3 Heat shock protein HSP 90 HSP90AB1

BX3 mRNA of PKA Catalytic Subunit C-alpha PRKACA

BX3 Dipeptidyl peptidase IV DPP4

BX3 "Phosphatidylinositol-4,5-bisphosphate 3-kinase catalytic subunit, gamma isoform" PIK3CG

BX3 "CGMP-inhibited 3',5'-cyclic phosphodiesterase A" PDE3A

BX3 Trypsin-1 PRSS1

BX3 Nuclear receptor coactivator 2 NCOA2

BX3 Nuclear receptor coactivator 1 NCOA1

BX3 Transcription factor p65 RELA

BX3 RAC-alpha serine/threonine-protein kinase AKT1

BX3 Vascular endothelial growth factor A VEGFA

BX3 Apoptosis regulator Bcl-2 BCL2

BX3 Proto-oncogene c-Fos FOS

BX3 Apoptosis regulator BAX BAX

BX3 Matrix metalloproteinase-9 MMP9

BX3 Caspase-3 CASP3

BX3 Cellular tumor antigen p53 TP53

BX3 Hypoxia-inducible factor 1-alphaHIF1A

BX3 Fos-related antigen 1 FOSL1

BX3 Fos-related antigen 2 FOSL2

BX3 Cell division control protein 2 homolog CDK1

BX3 G2/mitotic-specific cyclin-B1 CCNB1

BX3 Myeloperoxidase MPO

BX3 Aryl hydrocarbon receptor AHR

BX3 Insulin-like growth factor II IGF2

BX3 Cytochrome c CYCS

BX3 "Arachidonate 12-lipoxygenase, 12S-type" ALOX12

BX3 "Nuclear factor of activated T-cells, cytoplasmic 1" NFATC1

BX3 Tudor domain-containing protein 7 TDRD7

BX3 Egl nine homolog 1 EGLN1

BX3 NADPH oxidase 5 NOX5

BX3 "Fatty acid-binding protein, epidermal" FABP5

BX3 Apolipoprotein D APOD

BX4 Coagulation factor Xa F10

BX4 "mRNA of Protein-tyrosine phosphatase, non-receptor type 1" PTPN1

BX5 Progesterone receptor PGR

BX5 Nuclear receptor coactivator 2 NCOA2

BX5 Prostaglandin G/H synthase 1 PTGS1

BX5 Prostaglandin G/H synthase 2 PTGS2

BX5 Heat shock protein HSP 90 HSP90AB1

BX5 "Phosphatidylinositol-4,5-bisphosphate 3-kinase catalytic subunit, gamma isoform" PIK3CG

BX5 Potassium voltage-gated channel subfamily H member 2 KCNH2

BX5 mRNA of PKA Catalytic Subunit C-alpha PRKACA

BX5 Dopamine D1 receptor DRD1

BX5 Muscarinic acetylcholine receptor M3 CHRM3

BX5 Muscarinic acetylcholine receptor M1 CHRM1

BX5 Sodium channel protein type 5 subunit alpha SCN5A

BX5 Gamma-aminobutyric-acid receptor alpha-2 subunit GABRA2

BX5 Muscarinic acetylcholine receptor M4 CHRM4

BX5 "CGMP-inhibited 3',5'-cyclic phosphodiesterase A" PDE3A

BX5 5-hydroxytryptamine 2A receptor HTR2A

BX5 Gamma-aminobutyric-acid receptor alpha-5 subunit GABRA5

BX5 Alpha-1A adrenergic receptor ADRA1A

BX5 Gamma-aminobutyric-acid receptor alpha-3 subunit GABRB3

BX5 Muscarinic acetylcholine receptor M2 CHRM2

BX5 Alpha-1B adrenergic receptor ADRA1B

BX5 Beta-2 adrenergic receptor ADRB2

BX5 Neuronal acetylcholine receptor subunit alpha-2 CHRNA2

BX5 Sodium-dependent serotonin transporter SLC6A4

BX5 Mu-type opioid receptor OPRM1

BX5 Gamma-aminobutyric acid receptor subunit alpha-1 GABRA1

BX5 "Neuronal acetylcholine receptor protein, alpha-7 chain" CHRNA7

BX5 Cytochrome P450-cam camC

BX5 Apoptosis regulator Bcl-2 BCL2

BX5 Apoptosis regulator BAX BAX

BX5 Caspase-9 CASP9

BX5 Transcription factor AP-1 JUN

BX5 Caspase-3 CASP3

BX5 Caspase-8 CASP8

BX5 Protein kinase C alpha type PRKCA

BX5 Transforming growth factor beta-1 TGFB1

BX5 Serum paraoxonase/arylesterase 1PON1

BX5 Microtubule-associated protein 2MAP2

BX6 Progesterone receptor PGR

BX6 Mineralocorticoid receptor NR3C2

BX6 Nuclear receptor coactivator 2 NCOA2

BX6 Alcohol dehydrogenase 1C ADH1C

BX6 Retinoic acid receptor RXR-alphaRXRA

BX6 Nuclear receptor coactivator 1 NCOA1

BX6 Prostaglandin G/H synthase 1 PTGS1

BX6 Prostaglandin G/H synthase 2 PTGS2

BX6 Alpha-2A adrenergic receptor ADRA2A

BX6 Sodium-dependent noradrenaline transporter SLC6A2

BX6 Sodium-dependent dopamine transporter SLC6A3

BX6 Beta-2 adrenergic receptor ADRB2

BX6 Aldose reductase AKR1B1

BX6 Urokinase-type plasminogen activator PLAU

BX6 Leukotriene A-4 hydrolase LTA4H

BX6 Amine oxidase [flavin-containing] B MAOB

BX6 Amine oxidase [flavin-containing] A MAOA

BX6 mRNA of PKA Catalytic Subunit C-alpha PRKACA

BX6 Chymotrypsinogen B CTRB1

BX6 Muscarinic acetylcholine receptor M3 CHRM3

BX6 Muscarinic acetylcholine receptor M1 CHRM1

BX6 Beta-1 adrenergic receptor ADRB1

BX6 Sodium channel protein type 5 subunit alpha SCN5A

BX6 5-hydroxytryptamine 2A receptor HTR2A

BX6 Alpha-1A adrenergic receptor ADRA1A

BX6 Gamma-aminobutyric-acid receptor alpha-3 subunit GABRA3

BX6 Muscarinic acetylcholine receptor M2 CHRM2

BX6 Alpha-1B adrenergic receptor ADRA1B

BX6 Gamma-aminobutyric acid receptor subunit alpha-1 GABRA1

BX6 "Neuronal acetylcholine receptor protein, alpha-7 chain" CHRNA7

BX7 Prostaglandin G/H synthase 1 PTGS1

BX7 Nuclear receptor coactivator 2 NCOA2

BX8 Muscarinic acetylcholine receptor M3 CHRM3

BX8 Muscarinic acetylcholine receptor M1 CHRM1

BX8 Estrogen receptor ESR1

BX8 Androgen receptor AR

BX8 Sodium channel protein type 5 subunit alpha SCN5A

BX8 Peroxisome proliferator activated receptor gamma PPARG

BX8 Prostaglandin G/H synthase 2 PTGS2

BX8 Carbonic anhydrase II CA2

BX8 "CGMP-inhibited 3',5'-cyclic phosphodiesterase A" PDE3A

BX8 Alpha-1B adrenergic receptor ADRA1B

BX8 Beta-2 adrenergic receptor ADRB2

BX8 Alpha-1D adrenergic receptor ADRA1D

BX8 DNA topoisomerase 2-alpha TOP2A

BX8 Mu-type opioid receptor OPRM1

BX8 Cell division protein kinase 2 CDK2

BX8 "Neuronal acetylcholine receptor protein, alpha-7 chain" CHRNA7

BX8 Proto-oncogene serine/threonine-protein kinase Pim-1 PIM1

BX8 Cyclin-A2 CCNA2

BX8 Nuclear receptor coactivator 2 NCOA2

BX8 Nuclear receptor coactivator 1 NCOA1

BX9 Prostaglandin G/H synthase 1 PTGS1

BX9 Nuclear receptor coactivator 2 NCOA2

BX10 Androgen receptor AR

BX10 Prostaglandin G/H synthase 2 PTGS2

BX10 Beta-2 adrenergic receptor ADRB2

BX11 Mineralocorticoid receptor NR3C2

BX12 Purine nucleoside phosphorylase PNP

BX12 Prostaglandin G/H synthase 2 PTGS2

GL1 Glucocorticoid receptor NR3C1

GL2 "Nitric oxide synthase, inducible" NOS2

GL2 Prostaglandin G/H synthase 1 PTGS1

GL2 Prostaglandin G/H synthase 2 PTGS2

GL2 Dipeptidyl peptidase IV DPP4

GL2 Heat shock protein HSP 90   HSP90AB1

GL2 Trypsin-1 PRSS1

GL2 Nuclear receptor coactivator 2 NCOA2

GL2 Nuclear receptor coactivator 1 NCOA1

GL3 Coagulation factor Xa F10

GL4 "Nitric oxide synthase, inducible" NOS2

GL4 Prostaglandin G/H synthase 1 PTGS1

GL4 Prostaglandin G/H synthase 2 PTGS2

GL4 Dipeptidyl peptidase IV DPP4

GL4 Heat shock protein HSP 90   HSP90AB1

GL4 Trypsin-1 PRSS1

GL4 Nuclear receptor coactivator 2 NCOA2

GL4 "Phosphatidylinositol-4,5-bisphosphate 3-kinase catalytic subunit, gamma isoform" PIK3CG

GL5 Mineralocorticoid receptor NR3C2

GL6 Progesterone receptor PGR

GL6 Nuclear receptor coactivator 2 NCOA2

GL7 Prostaglandin G/H synthase 1 PTGS1

GL7 Prostaglandin G/H synthase 2 PTGS2

GL7 Nuclear receptor coactivator 2 NCOA2

GL8 Progesterone receptor PGR

GL8 Mineralocorticoid receptor NR3C2

GL8 Nuclear receptor coactivator 2 NCOA2

GL9 Progesterone receptor PGR

GL9 Mineralocorticoid receptor NR3C2

GL10 Prostaglandin G/H synthase 1 PTGS1

GL10 Prostaglandin G/H synthase 2 PTGS2
