# Supplementary material for: Anticancer Action of Xiaoxianxiong Tang in Non-Small Cell Lung Cancer by Pharmacological Analysis and Experimental Validation
Source: Evid Based Complement Alternat Med. 2021 Dec 13;2021:9930082. doi: 10.1155/2021/9930082 (PMC8687818; doi:10.1155/2021/9930082)
Supplement: Supplementary Materials — All primer sets in the RT-qPCR array are shown in the Table 1. Table 2 indicates the targets in XXXT. Table 3 indicates the targets related to NSCLC. Table 4 indicates common targets of NSCLC and XXXT. Table 5 indicates the result of the RT-qPCR array in H460 cells. Table 5 indicates the result of the RT-qPCR array in A549 cells. [file 9930082.f1.zip › 9930082.f1/Supplementary table 3-Disease targets related to NSCLC.pdf]

Therapeutic Target Database

Gene_Full_Name	Gene Symbol
Arachidonate 5-lipoxygenase	5-LOX
Multidrug resistance protein 1	ABCB1
ALK tyrosine kinase receptor	ALK
ALK tyrosine kinase receptor	ALK
ATM serine/threonine kinase	ATM
Tyrosine-protein kinase UFO	AXL
Apoptosis inhibitor survivin	BIRC5
Activation B7-1 antigen	CD80
Cyclin-dependent kinase	CDK
Cyclin-dependent kinase 1	CDK1
Cyclin-dependent kinase 2	CDK2
Cyclin-dependent kinase 7	CDK7
Checkpoint kinase-1	CHK1
Dopamine D2 receptor	D2R
DNA [cytosine-5]-methyltransferase 1	DNMT1
Dual specificity protein phosphatase 1	DUSP1
Epidermal growth factor-like protein 7	EGFL7
Epidermal growth factor receptor	EGFR
Ephrin type-B receptor 4	EPHB4
Erbb3 tyrosine kinase receptor	Erbb-3
Erbb4 tyrosine kinase receptor	Erbb-4
Fibroblast growth factor receptor 1	FGFR1

Fms-like tyrosine kinase 3   FLT-3

CAAX farnesyltransferase beta   FNTB

Farnesyl protein transferase Ftase

Guanine nucleotide exchange factor GNEF

Erbb2 tyrosine kinase receptor HER2

HGF/Met signaling pathway   HGF/Met pathway

Endoplasmin   HSP90B1

"Indoleamine 2,3-dioxygenase 1" IDO1

Insulin-like growth factor I receptor IGF1R

Interleukin 2 receptor   IL2R

Integrin alpha-5/beta-1 ITGA5/B1

Integrin beta-1   ITGB1

Vascular endothelial growth factor receptor 2 KDR

Tyrosine-protein kinase Kit KIT

Low-density lipoprotein receptorLDL-R

Melanoma-associated antigen 3   MAGEA3

Cancer/testis antigen MAGE-C1/CT7   MAGEC1

Melanoma-associated antigen C2 MAGEC2

Plasmodium Dihydroorotate dehydrogenase Malaria DHOdehase

MAPK/ERK kinase kinase   MAP3K

ERK activator kinase 1   MEK1

ERK activator kinase 2   MEK2

Proto-oncogene c-Met MET

Opioid receptor mu   MOP

M-phase inducer phosphatase 2   MPIP2

Serine/threonine-protein kinase mTOR   mTOR

Mucin-1MUC1

GTPase NRas   NRAS

Nuclear factor erythroid 2-related factor 2   Nrf2

Cancer/testis antigen 1 NY-ESO-1

Programmed cell death protein 1 PD-1

Platelet-derived growth factor receptorPDGFR

Platelet-derived growth factor receptor alpha PDGFRA

Platelet-derived growth factor receptor beta   PDGFRB

Rotamase Pin1 PIN1

Protein kinasePK

Prolyl endopeptidase PREP

PKC-alpha messenger RNA PRKCA mRNA

Tyrosine-protein kinase PTK

Proto-oncogene c-Ros ROS1

Proto-oncogene c-Ros ROS1

Sphingosine-1-phosphate receptor 1 S1PR1

Toll-like receptor   TLR

Oncotrophoblast glycoprotein 5T4TPBG

TRAIL receptor 1 TRAIL-R1

Tropomyosin-related kinase A TrkA

BDNF/NT-3 growth factors receptor   TrkB

NT-3 growth factor receptor TrkC

Tubulin beta	TUBB

Tumor suppressor candidate 2 TUSC2

Vascular endothelial growth factor A	VEGFA

Vascular endothelial growth factor receptor	VEGFR

DrugBank database

Gene_Full_Name Gene Symbol

Serum albumin ALB

Serum albumin ALB

Serum albumin ALB

Alpha-1-acid glycoprotein 1 ORM1

Serum albumin ALB

Prostaglandin G/H synthase 2 PTGS2

Myeloperoxidase	MPO

Xanthine dehydrogenase/oxidase	XDH

Arylamine N-acetyltransferase	NAT1

Cytochrome P450 2C9	CYP2C9

Cytochrome P450 2B6	CYP2B6

Cholinesterase BCHE

Cytochrome P450 4A11 CYP4A11

Glutathione S-transferase theta-1	GSTT1

Metallothionein-1A	MT1A

Metallothionein-2 MT2A

Superoxide dismutase [Cu-Zn] SOD1

Glutathione S-transferase P GSTP1

NAD(P)H dehydrogenase [quinone] 1 NQO1

Glutathione S-transferase Mu 1 GSTM1

Cytochrome P450 2D6 CYP2D6

Cytochrome P450 3A4 CYP3A4

Cytochrome P450 2C9 CYP2C9

UDP-glucuronosyltransferase 1-1 UGT1A1

Cytochrome P450 3A4 CYP3A4

Cytochrome P450 3A4 CYP3A4

Cytochrome P450 3A5 CYP3A5

Cytochrome P450 1A1 CYP1A1

Cytochrome P450 1A2 CYP1A2

Cytochrome P450 1B1 CYP1B1

Cytochrome P450 2D6 CYP2D6

Cytochrome P450 2C8 CYP2C8

UDP-glucuronosyltransferase 1-1 UGT1A1

Cytochrome P450 3A4 CYP3A4

Cytochrome P450 1A2 CYP1A2

Cytochrome P450 2E1 CYP2E1

Cytochrome P450 3A5 CYP3A5

UDP-glucuronosyltransferase 1-1 UGT1A1

Glutathione S-transferase theta-1 GSTT1

Glutathione S-transferase P GSTP1

Prostaglandin G/H synthase 2 PTGS2

Prostaglandin G/H synthase 1 PTGS1

Cytochrome P450 3A4 CYP3A4

Cytochrome P450 3A5 CYP3A5

UDP-glucuronosyltransferase 1-1 UGT1A1

UDP-glucuronosyltransferase 1-9 UGT1A9

Liver carboxylesterase 1 CES1

Cytochrome P450 3A7 CYP3A7

Cytochrome P450 2B6 CYP2B6

Cocaine esterase CES2

Cholinesterase BCHE

NADPH--cytochrome P450 reductase POR

Cytochrome P450 2C8 CYP2C8

Cytochrome P450 3A4 CYP3A4

Cytochrome P450 3A5 CYP3A5

Cytochrome P450 3A7 CYP3A7

Cytochrome P450 19A1 CYP19A1

Cytochrome P450 1B1 CYP1B1

Cytochrome P450 3A4 CYP3A4

Cytochrome P450 2D6 CYP2D6

DNA DNMT3A

DNA-3-methyladenine glycosylase MPG

Alpha-2-macroglobulin A2M

Serotransferrin TF

Copper transport protein ATOX1 ATOX1

Epidermal growth factor receptorEGFR

High affinity nerve growth factor receptor NTRK1

BDNF/NT-3 growth factors receptor NTRK2

NT-3 growth factor receptor NTRK3

Proto-oncogene tyrosine-protein kinase ROS ROS1

Tyrosine-protein kinase JAK2 JAK2

Activated CDC42 kinase 1 TNK2

Epidermal growth factor receptorEGFR

Nuclear receptor subfamily 1 group I member 2 NR1I2

DNA topoisomerase 2-alpha   TOP2A

DNA topoisomerase 2-beta TOP2B

"DNA topoisomerase I, mitochondrial"   TOP1MT

DNA topoisomerase 1 TOP1

DNA DNMT3A

Apoptosis regulator Bcl-2   BCL2

Tubulin beta-1 chain TUBB1

Nuclear receptor subfamily 1 group I member 2 NR1I2

Microtubule-associated protein 4MAP4

Microtubule-associated protein 2MAP2

Microtubule-associated protein tau MAPT

Tubulin beta chain   TUBB

Transcription factor AP-1   JUN

Tubulin alpha-1A chain   TUBA1A

Tubulin delta chain TUBD1

Tubulin epsilon chain TUBE1

Tubulin gamma-1 chain TUBG1

Canalicular multispecific organic anion transporter 2 ABCC3

Multidrug resistance-associated protein 5 ABCC5

Canalicular multispecific organic anion transporter 1 ABCC2

Solute carrier family 22 member 2 SLC22A2

High affinity copper uptake protein 1 SLC31A1

Probable low affinity copper uptake protein 2 SLC31A2

Multidrug resistance-associated protein 6 ABCC6

P-glycoprotein 1 ABCB1

Copper-transporting ATPase 2 ATP7B

Copper-transporting ATPase 1 ATP7A

ATP-binding cassette sub-family G member 2 ABCG2

P-glycoprotein 1 ABCB1

ATP-binding cassette sub-family G member 2 ABCG2

Solute carrier family 22 member 1 SLC22A1

P-glycoprotein 1 ABCB1

ATP-binding cassette sub-family G member 2 ABCG2

P-glycoprotein 1 ABCB1

Solute carrier organic anion transporter family member 2B1 SLCO2B1

Canalicular multispecific organic anion transporter 2 ABCC3

Multidrug resistance-associated protein 6 ABCC6

P-glycoprotein 1 ABCB1

Multidrug resistance-associated protein 1 ABCC1

Multidrug resistance-associated protein 7 ABCC10

Canalicular multispecific organic anion transporter 1 ABCC2

ATP-binding cassette sub-family G member 2 ABCG2

Solute carrier family 22 member 3 SLC22A3

Solute carrier organic anion transporter family member 1B1 SLCO1B1

Multidrug resistance-associated protein 1 ABCC1

ATP-binding cassette sub-family G member 2 ABCG2

P-glycoprotein 1 ABCB1

Canalicular multispecific organic anion transporter 1 ABCC2

Bile salt export pump ABCB11

P-glycoprotein 1 ABCB1

Multidrug resistance-associated protein 1 ABCC1

Multidrug resistance-associated protein 7 ABCC10

Solute carrier organic anion transporter family member 1B3 SLCO1B3

Canalicular multispecific organic anion transporter 1 ABCC2

P-glycoprotein 1 ABCB1

Multidrug resistance-associated protein 1 ABCC1

Canalicular multispecific organic anion transporter 1 ABCC2

Multidrug resistance-associated protein 6 ABCC6

Bile salt export pump ABCB11

Solute carrier organic anion transporter family member 1B1

SLCO1B1

GeneCards database

Gene_Full_Name Gene Symbol

Epidermal Growth Factor Receptor EGFR

Tumor Protein P53 TP53

"KRAS Proto-Oncogene, GTPase" KRAS

Cyclin Dependent Kinase Inhibitor 2A CDKN2A

Erb-B2 Receptor Tyrosine Kinase 2 ERBB2

"B-Raf Proto-Oncogene, Serine/Threonine Kinase" BRAF

ALK Receptor Tyrosine Kinase ALK

"Phosphatidylinositol-4,5-Bisphosphate 3-Kinase Catalytic Subunit Alpha" PIK3CA

Phosphatase And Tensin Homolog PTEN

BRCA2 DNA Repair Associated BRCA2

AKT Serine/Threonine Kinase 1 AKT1

Vascular Endothelial Growth Factor A VEGFA

Serine/Threonine Kinase 11 STK11

BRCA1 DNA Repair Associated BRCA1

Cadherin 1 CDH1

MutL Homolog 1 MLH1

"MET Proto-Oncogene, Receptor Tyrosine Kinase" MET

Cyclin D1 CCND1

Interleukin 6 IL6

APC Regulator Of WNT Signaling Pathway APC

Signal Transducer And Activator Of Transcription 3 STAT3

ATM Serine/Threonine Kinase ATM

"NRAS Proto-Oncogene, GTPase" NRAS

"HRas Proto-Oncogene, GTPase" HRAS

Transforming Growth Factor Beta 1 TGFB1

Telomerase Reverse Transcriptase TERT

Catenin Beta 1 CTNNB1

"ERCC Excision Repair 1, Endonuclease Non-Catalytic Subunit" ERCC1

RB Transcriptional Corepressor 1 RB1

Tumor Necrosis Factor TNF

Cyclin Dependent Kinase Inhibitor 1A CDKN1A

"MYC Proto-Oncogene, BHLH Transcription Factor" MYC

Notch Receptor 1 NOTCH1

Epidermal Growth Factor EGF

"ERCC Excision Repair 2, TFIIH Core Complex Helicase Subunit" ERCC2

"BCL2 Associated X, Apoptosis Regulator" BAX

Cyclin Dependent Kinase 4 CDK4

Mitogen-Activated Protein Kinase Kinase 1 MAP2K1

NK2 Homeobox 1 NKX2-1

Mechanistic Target Of Rapamycin Kinase MTOR

Checkpoint Kinase 2 CHEK2

Estrogen Receptor 1	ESR1

SMAD Family Member 4	SMAD4

Fragile Histidine Triad Diadenosine Triphosphatase	FHIT

Retinoic Acid Receptor Beta	RARB

MicroRNA 21	MIR21

"KIT Proto-Oncogene, Receptor Tyrosine Kinase"	KIT

Interleukin 10	IL10

MDM2 Proto-Oncogene	MDM2

Discoidin Domain Receptor Tyrosine Kinase 2	DDR2

Caspase 8	CASP8

Cyclin Dependent Kinase Inhibitor 1B	CDKN1B

Tumor Protein P63	TP63

Fibroblast Growth Factor Receptor 1	FGFR1

Fas Cell Surface Death Receptor	FAS

Thymidylate Synthetase	TYMS

C-X-C Motif Chemokine Receptor 4	CXCR4

"ROS Proto-Oncogene 1, Receptor Tyrosine Kinase"	ROS1

Ret Proto-Oncogene	RET

O-6-Methylguanine-DNA Methyltransferase	MGMT

"SRC Proto-Oncogene, Non-Receptor Tyrosine Kinase"	SRC

Transforming Growth Factor Beta Receptor 2	TGFBR2

"Nuclear Factor, Erythroid 2 Like 2"	NFE2L2

AKT Serine/Threonine Kinase 2	AKT2

Secreted Phosphoprotein 1	SPP1

Nuclear Factor Kappa B Subunit 1NFKB1

CD274 MoleculeCD274

Programmed Cell Death 1 PDCD1

Caveolin 1 CAV1

Fibroblast Growth Factor Receptor 2 FGFR2

Erb-B2 Receptor Tyrosine Kinase 3 ERBB3

Enhancer Of Zeste 2 Polycomb Repressive Complex 2 SubunitEZH2

Estrogen Receptor 2 ESR2

BRCA1 Associated Protein 1 BAP1

"Raf-1 Proto-Oncogene, Serine/Threonine Kinase" RAF1

Peroxisome Proliferator Activated Receptor Gamma PPARG

BCL2 Apoptosis Regulator BCL2

Fibroblast Growth Factor Receptor 3 FGFR3

MicroRNA 145 MIR145

Fas Ligand FASLG

"Plasminogen Activator, Urokinase" PLAU

Thymidine Phosphorylase TYMP

SRY-Box Transcription Factor 2 SOX2

ATP Binding Cassette Subfamily B Member 1 ABCB1

Platelet Derived Growth Factor Receptor Beta PDGFRB

Matrix Metallopeptidase 1   MMP1

Solute Carrier Family 2 Member 1SLC2A1

C-X-C Motif Chemokine Ligand 12 CXCL12

Prostaglandin-Endoperoxide Synthase 2 PTGS2

RAD51 Recombinase RAD51

Matrix Metallopeptidase 9 MMP9

Fms Related Receptor Tyrosine Kinase 1 FLT1

X-Linked Inhibitor Of Apoptosis XIAP

TNF Receptor Superfamily Member 10b TNFRSF10B

Snail Family Transcriptional Repressor 2 SNAI2

"SWI/SNF Related, Matrix Associated, Actin Dependent Regulator Of Chromatin, Subfamily A, Member 4" SMARCA4

DNA Topoisomerase II Alpha TOP2A

Toll Like Receptor 4 TLR4

Telomerase RNA Component TERC

MicroRNA 17 MIR17

Phosphoinositide-3-Kinase Regulatory Subunit 1 PIK3R1

X-Ray Repair Cross Complementing 1 XRCC1

Zinc Finger E-Box Binding Homeobox 1 ZEB1

DLEC1 Cilia And Flagella Associated Protein DLEC1

Epithelial Cell Adhesion Molecule EPCAM

E1A Binding Protein P300 EP300

MicroRNA 126 MIR126

Tumor Protein P73 TP73

MicroRNA 34a MIR34A

DNA Methyltransferase 1 DNMT1

H19 Imprinted Maternally Expressed Transcript H19

Insulin Like Growth Factor 2 IGF2

Erb-B2 Receptor Tyrosine Kinase 4 ERBB4

"RELA Proto-Oncogene, NF-KB Subunit" RELA

Platelet Derived Growth Factor Receptor Alpha PDGFRA

8-Oxoguanine DNA Glycosylase OGG1

Mitogen-Activated Protein Kinase 1 MAPK1

SMAD Family Member 3 SMAD3

Signal Transducer And Activator Of Transcription 1 STAT1

Prominin 1 PROM1

GLI Family Zinc Finger 1 GLI1

Forkhead Box P3 FOXP3

Cytochrome P450 Family 1 Subfamily B Member 1 CYP1B1

Heat Shock Protein Family B (Small) Member 1 HSPB1

MicroRNA 155 MIR155

MicroRNA 125a MIR125A

MicroRNA 200c MIR200C

Janus Kinase 2 JAK2

Baculoviral IAP Repeat Containing 5 BIRC5

Twist Family BHLH Transcription Factor 1 TWIST1

Aurora Kinase A AURKA

Cytotoxic T-Lymphocyte Associated Protein 4 CTLA4

MicroRNA 143 MIR143

MicroRNA 205 MIR205

Dihydropyrimidine Dehydrogenase DPYD

Axin 2 AXIN2

Maternally Expressed 3 MEG3

MicroRNA 146a MIR146A

X-Ray Repair Cross Complementing 3 XRCC3

Cyclin Dependent Kinase Inhibitor 2B CDKN2B

MutS Homolog 3 MSH3

Nitric Oxide Synthase 2 NOS2

Colony Stimulating Factor 3 CSF3

Matrix Metallopeptidase 2 MMP2

AKT Serine/Threonine Kinase 3 AKT3

C-C Motif Chemokine Receptor 6 CCR6

MicroRNA 31 MIR31

Caspase 3 CASP3

MicroRNA 150 MIR150

"A-Raf Proto-Oncogene, Serine/Threonine Kinase" ARAF

Glutathione S-Transferase Pi 1 GSTP1

MicroRNA 142 MIR142

CAMP Responsive Element Binding Protein 1 CREB1

Interferon Regulatory Factor 1 IRF1

MicroRNA 182 MIR182

MicroRNA 214 MIR214

Hypoxia Inducible Factor 1 Subunit Alpha HIF1A

MicroRNA 221 MIR221

Aryl Hydrocarbon Receptor AHR

MicroRNA 148a MIR148A

Ras Association Domain Family Member 1 RASSF1

Solute Carrier Family 22 Member 18 SLC22A18

Von Hippel-Lindau Tumor Suppressor VHL

Platelet Derived Growth Factor Subunit B PDGFB

MicroRNA 200a MIR200A

MicroRNA 200b MIR200B

MicroRNA 193a MIR193A

Baculoviral IAP Repeat Containing 3 BIRC3

MicroRNA Let-7a-1 MIRLET7A1

Retinoic Acid Receptor Alpha RARA

C-X-C Motif Chemokine Ligand 8 CXCL8

MicroRNA 29a MIR29A

Checkpoint Kinase 1 CHEK1

Bone Morphogenetic Protein 2 BMP2

MicroRNA 27a MIR27A

EGFR Antisense RNA 1 EGFR-AS1

CEA Cell Adhesion Molecule 5 CEACAM5

"XPA, DNA Damage Recognition And Repair Factor" XPA

MicroRNA 25 MIR25

MicroRNA 141 MIR141

MicroRNA 140 MIR140

MicroRNA 29c MIR29C

DNA Methyltransferase 3 Alpha DNMT3A

Neurotrophic Receptor Tyrosine Kinase 1 NTRK1

MicroRNA 183 MIR183

MicroRNA 195 MIR195

ATP Binding Cassette Subfamily C Member 2 ABCC2

Insulin Like Growth Factor 1 Receptor IGF1R

MicroRNA 15a MIR15A

MicroRNA 144 MIR144

"Mucin 1, Cell Surface Associated" MUC1

NFKB Inhibitor Alpha NFKBIA

Gap Junction Protein Alpha 1 GJA1

"SET Domain Containing 2, Histone Lysine Methyltransferase" SETD2

MicroRNA 203a MIR203A

Cholinergic Receptor Nicotinic Alpha 5 Subunit CHRNA5

Cholinergic Receptor Nicotinic Alpha 3 Subunit CHRNA3

MicroRNA 223 MIR223

"MYCN Proto-Oncogene, BHLH Transcription Factor" MYCN

Cytochrome P450 Family 2 Subfamily A Member 6 CYP2A6

"ERCC Excision Repair 5, Endonuclease" ERCC5

TNF Receptor Superfamily Member 1A TNFRSF1A

MicroRNA 222 MIR222

Zinc Finger E-Box Binding Homeobox 2 ZEB2

Mitogen-Activated Protein Kinase Kinase 2 MAP2K2

Zinc Finger MYND-Type Containing 10 ZMYND10

MicroRNA 181a-1 MIR181A1

Kruppel Like Factor 6 KLF6

Integrin Subunit Alpha 3 ITGA3

"ERCC Excision Repair 6, Chromatin Remodeling Factor" ERCC6

Matrix Remodeling Associated 5 MXRA5

15-Hydroxyprostaglandin Dehydrogenase HPGD

Glutathione S-Transferase Mu 1 GSTM1

Insulin Receptor Substrate 1 IRS1

Signal Transducer And Activator Of Transcription 5B STAT5B

MicroRNA 96 MIR96

Phosphoinositide-3-Kinase Regulatory Subunit 2 PIK3R2

MicroRNA 486-1 MIR486-1

Keratin 19 KRT19

Cytochrome P450 Family 2 Subfamily D Member 6 CYP2D6

MicroRNA 210 MIR210

MicroRNA 106b MIR106B

MicroRNA 16-1 MIR16-1

MicroRNA 99a MIR99A

High Mobility Group AT-Hook 2 HMGA2

MicroRNA 204 MIR204

MicroRNA 30e MIR30E

Ornithine Decarboxylase 1 ODC1

Collagen Type XVIII Alpha 1 Chain COL18A1

MicroRNA 20a MIR20A

UDP Glucuronosyltransferase Family 1 Member A1 UGT1A1

Methylthioadenosine Phosphorylase MTAP

MicroRNA Let-7b MIRLET7B

Parkin RBR E3 Ubiquitin Protein Ligase PRKN

MicroRNA 18a MIR18A

MicroRNA 93 MIR93

MicroRNA 26a-1 MIR26A1

Kinase Insert Domain Receptor KDR

MicroRNA Let-7d MIRLET7D

Enolase 2 ENO2

MicroRNA 34c MIR34C

Transcription Factor AP-2 Alpha TFAP2A

Beta-2-Microglobulin B2M

MicroRNA 107 MIR107

MicroRNA 22 MIR22

MicroRNA 335 MIR335

MicroRNA 23a MIR23A

MicroRNA 424 MIR424

Vascular Endothelial Growth Factor C VEGFC

CD40 Molecule CD40

MicroRNA 100 MIR100

Protein Phosphatase 2 Scaffold Subunit Abeta PPP2R1B

MicroRNA 451a MIR451A

"Major Histocompatibility Complex, Class I, G" HLA-G

Cytochrome P450 Family 1 Subfamily A Member 1 CYP1A1

BUB1 Mitotic Checkpoint Serine/Threonine Kinase BUB1

GATA Binding Protein 2 GATA2

"ERCC Excision Repair 4, Endonuclease Catalytic Subunit" ERCC4

MicroRNA 193b MIR193B

MicroRNA 32 MIR32

MicroRNA 373 MIR373

MicroRNA 137 MIR137

ATP Binding Cassette Subfamily C Member 1 ABCC1

MicroRNA 133b MIR133B

MicroRNA 224 MIR224

Janus Kinase 3 JAK3

MicroRNA 34b MIR34B

KCNQ1 Opposite Strand/Antisense Transcript 1 KCNQ1OT1

SOS Ras/Rac Guanine Nucleotide Exchange Factor 1 SOS1

MicroRNA 185 MIR185

EMAP Like 4 EML4

MicroRNA 19a MIR19A

MicroRNA 148b MIR148B

U2 Small Nuclear RNA Auxiliary Factor 1 U2AF1

Caspase 9 CASP9

MicroRNA 483 MIR483

"Jun Proto-Oncogene, AP-1 Transcription Factor Subunit" JUN

Thyroid Hormone Receptor Interactor 13 TRIP13

Damage Specific DNA Binding Protein 2 DDB2

MicroRNA 106a MIR106A

MicroRNA 296 MIR296

Interleukin 4 Receptor   IL4R

MicroRNA 30b MIR30B

MicroRNA Let-7e   MIRLET7E

DNA Topoisomerase I TOP1

Myocardial Infarction Associated Transcript   MIAT

DNA Polymerase Kappa POLK

CD44 Molecule (Indian Blood Group) CD44

MicroRNA 199b MIR199B

MicroRNA 98   MIR98

BCL2 Like 1   BCL2L1

E2F Transcription Factor 1 E2F1

MicroRNA 192 MIR192

Hepatitis A Virus Cellular Receptor 2 HAVCR2

Mitogen-Activated Protein Kinase 3 MAPK3

MicroRNA 378a MIR378A

MicroRNA 146b MIR146B

MicroRNA 342 MIR342

MicroRNA 499a MIR499A

MicroRNA 30d MIR30D

Transforming Growth Factor AlphaTGFA

Mitogen-Activated Protein Kinase 8 MAPK8

MicroRNA 130a MIR130A

Gastrin Releasing Peptide GRP

Fibroblast Growth Factor 2 FGF2

TNF Superfamily Member 10 TNFSF10

Hepatocyte Growth Factor HGF

MicroRNA Let-7g MIRLET7G

MicroRNA 15b MIR15B

Insulin Like Growth Factor 1 IGF1

MicroRNA Let-7c MIRLET7C

MicroRNA 191 MIR191

Achaete-Scute Family BHLH Transcription Factor 1 ASCL1

MicroRNA 29b-2 MIR29B2

Marker Of Proliferation Ki-67 MKI67

MicroRNA 331 MIR331

NAD(P)H Quinone Dehydrogenase 1 NQO1

MicroRNA 10a MIR10A

MicroRNA 324 MIR324

ATP Binding Cassette Subfamily G Member 2 (Junior Blood Group) ABCG2

Laminin Subunit Beta 3 LAMB3

MicroRNA 429 MIR429

MicroRNA 377 MIR377

MicroRNA 128-2 MIR128-2

MicroRNA 33a MIR33A

MicroRNA 197 MIR197

Mitotic Arrest Deficient 1 Like 1 MAD1L1

Proliferating Cell Nuclear Antigen PCNA

MicroRNA 372 MIR372

MYC Associated Factor X MAX

Poly(ADP-Ribose) Polymerase 1 PARP1

MicroRNA 198 MIR198

MicroRNA Let-7i MIRLET7I

"Actin Alpha 2, Smooth Muscle" ACTA2

Protein Tyrosine Kinase 2 PTK2

Ribonucleotide Reductase Catalytic Subunit M1 RRM1

"Transporter 1, ATP Binding Cassette Subfamily B Member" TAP1

Delta Like Non-Canonical Notch Ligand 1 DLK1

Interleukin 2 IL2

Neurofibromin 1 NF1

"MCL1 Apoptosis Regulator, BCL2 Family Member" MCL1

MutS Homolog 2 MSH2

MicroRNA Let-7a-3 MIRLET7A3

Integrin Subunit Alpha 2b ITGA2B

MicroRNA 24-2 MIR24-2

"Cytochrome C, Somatic" CYCS

TIMP Metallopeptidase Inhibitor 1 TIMP1

Metastasis Associated Lung Adenocarcinoma Transcript 1 MALAT1

Matrix Metallopeptidase 7 MMP7

Chromogranin A CHGA

Transforming Growth Factor Beta Receptor 1 TGFBR1

Fms Related Receptor Tyrosine Kinase 4 FLT4

Protein Kinase C Iota PRKCI

Albumin ALB

Insulin Like Growth Factor Binding Protein 3 IGFBP3

Major Vault Protein MVP

Tyrosine 3-Monooxygenase/Tryptophan 5-Monooxygenase Activation Protein Epsilon YWHAE

Colony Stimulating Factor 2 CSF2

Cyclin B1 CCNB1

BCL2 Antagonist/Killer 1 BAK1

Interleukin 4 IL4

TSC Complex Subunit 2 TSC2

Kelch Like ECH Associated Protein 1 KEAP1

Protein Kinase C Alpha PRKCA

"Mucin 16, Cell Surface Associated" MUC16

Mitogen-Activated Protein Kinase 14 MAPK14

Cyclin Dependent Kinase 2 CDK2

"Plasminogen Activator, Urokinase Receptor" PLAUR

RNA Component Of Mitochondrial RNA Processing Endoribonuclease RMRP

Death Associated Protein Kinase 1 DAPK1

Synaptophysin SYP

Cyclin A2 CCNA2

Semaphorin 3B SEMA3B

Snail Family Transcriptional Repressor 1 SNAI1

Progesterone Receptor PGR

Mitogen-Activated Protein Kinase Kinase Kinase 8 MAP3K8

Sp1 Transcription Factor SP1

Cell Adhesion Molecule 1 CADM1

Cyclin Dependent Kinase 6 CDK6

BCL2 Like 11 BCL2L11

Heat Shock Protein 90 Alpha Family Class A Member 1 HSP90AA1

Keratin 7 KRT7

Cyclin Dependent Kinase 1 CDK1

NME/NM23 Nucleoside Diphosphate Kinase 1 NME1

Transketolase TKT

Apurinic/Apyrimidinic Endodeoxyribonuclease 1 APEX1

Rac Family Small GTPase 1 RAC1

Amphiregulin AREG

MicroRNA 181c MIR181C

Fibroblast Growth Factor Receptor 4 FGFR4

TIMP Metallopeptidase Inhibitor 2 TIMP2

WT1 Transcription Factor WT1

Yes Associated Protein 1 YAP1

Integrin Subunit Beta 1 ITGB1

Superoxide Dismutase 2 SOD2

Forkhead Box M1 FOXM1

S-Phase Kinase Associated Protein 2 SKP2

Keratin 18 KRT18

Fibronectin 1 FN1

MicroRNA 30a MIR30A

HOX Transcript Antisense RNA HOTAIR

High Mobility Group Box 1 HMGB1

Heme Oxygenase 1 HMOX1

"Phosphatidylinositol-4,5-Bisphosphate 3-Kinase Catalytic Subunit Gamma" PIK3CG

POU Class 5 Homeobox 1 POU5F1

Surfactant Protein C SFTPC

BCL2 Associated Agonist Of Cell Death BAD

"Fos Proto-Oncogene, AP-1 Transcription Factor Subunit" FOS

Serpin Family A Member 1 SERPINA1

Neuregulin 1 NRG1

"Dicer 1, Ribonuclease III" DICER1

Stathmin 1 STMN1

Angiopoietin 2 ANGPT2

Histone Deacetylase 1 HDAC1

Polo Like Kinase 1 PLK1

CASP8 And FADD Like Apoptosis Regulator CFLAR

Interferon Alpha Inducible Protein 27 IFI27

Carbonic Anhydrase 9 CA9

Interleukin 1 Beta IL1B

S100 Calcium Binding Protein A4 S100A4

Aldehyde Dehydrogenase 1 Family Member A1 ALDH1A1

Annexin A5 ANXA5

Thrombospondin 1 THBS1

Matrix Metallopeptidase 14 MMP14

Ras Homolog Family Member A RHOA

TIMP Metallopeptidase Inhibitor 3 TIMP3

Protein Tyrosine Phosphatase Non-Receptor Type 11 PTPN11

Growth Arrest And DNA Damage Inducible Alpha GADD45A

Bone Morphogenetic Protein 4 BMP4

Cellular Communication Network Factor 2 CCN2

"BMI1 Proto-Oncogene, Polycomb Ring Finger" BMI1

Cadherin 13 CDH13

AXL Receptor Tyrosine Kinase AXL

Catalase CAT

Cancer/Testis Antigen 1B CTAG1B

Vascular Endothelial Growth Factor D VEGFD

Surfactant Protein D SFTPD

Surfactant Protein B SFTPB

Serpin Family B Member 5 SERPINB5

Heat Shock Protein Family A (Hsp70) Member 5 HSPA5

Retinoid X Receptor Gamma RXRG

Histone Deacetylase 9 HDAC9

TNF Receptor Superfamily Member 10a TNFRSF10A

RUNX Family Transcription Factor 3 RUNX3

Forkhead Box O3   FOXO3

Basigin (Ok Blood Group) BSG

Ribosomal Protein S6 Kinase B1 RPS6KB1

Cytochrome P450 Family 2 Subfamily E Member 1 CYP2E1

Tubulin Beta Class I TUBB

DNA Methyltransferase 3 Beta DNMT3B

Neural Cell Adhesion Molecule 1 NCAM1

Cadherin 2 CDH2

Matrix Metallopeptidase 3   MMP3

"Mucin 5AC, Oligomeric Mucus/Gel-Forming" MUC5AC

Galectin 3 LGALS3

Macrophage Migration Inhibitory Factor MIF

Enolase 1 ENO1

Periostin POSTN

Pvt1 Oncogene PVT1

Dihydrofolate Reductase DHFR

EPH Receptor A2   EPHA2

E2F Transcription Factor 3 E2F3

SMAD Family Member 2 SMAD2

Neuropilin 1 NRP1

Beclin 1 BECN1

Secreted Protein Acidic And Cysteine Rich SPARC

Cytochrome P450 Family 3 Subfamily A Member 4 CYP3A4

Interferon Gamma IFNG

Heat Shock Protein Family A (Hsp70) Member 4 HSPA4

Cathepsin B CTSB

Fascin Actin-Bundling Protein 1 FSCN1

GNAS Complex Locus GNAS

MicroRNA 375 MIR375

Growth Arrest Specific 5 GAS5

S100 Calcium Binding Protein A2 S100A2

Keratin 8 KRT8

Thioredoxin TXN

Catenin Delta 1 CTNND1

Interleukin 3 IL3

Toll Like Receptor 9 TLR9

Deoxycytidine Kinase DCK

Fatty Acid Synthase FASN

"Phosphatidylinositol-4,5-Bisphosphate 3-Kinase Catalytic Subunit Beta" PIK3CB

Serpin Family A Member 3 SERPINA3

Junction Plakoglobin JUP

Isocitrate Dehydrogenase (NADP(+)) 1 IDH1

Keratin 20 KRT20

MicroRNA 124-1 MIR124-1

BPI Fold Containing Family A Member 1 BPIFA1

"Mucin 4, Cell Surface Associated" MUC4

Parathyroid Hormone Like HormonePTHLH

Ubiquitin C-Terminal Hydrolase L1 UCHL1

C-C Motif Chemokine Receptor 7 CCR7

Diablo IAP-Binding Mitochondrial Protein DIABLO

Lysyl Oxidase LOX

Ezrin EZR

Growth Factor Receptor Bound Protein 2 GRB2

CD82 Molecule CD82

Retinoid X Receptor Alpha RXRA

MAGE Family Member A3 MAGEA3

MicroRNA 9-1 MIR9-1

Y-Box Binding Protein 1 YBX1

N-Acetyltransferase 2 NAT2

3-Phosphoinositide Dependent Protein Kinase 1 PDPK1

Paxillin PXN

Cyclin E1 CCNE1

Taurine Up-Regulated 1 TUG1

Napsin A Aspartic Peptidase NAPSA

C-X-C Motif Chemokine Ligand 1 CXCL1

Cell Division Cycle 42 CDC42

Galectin 1 LGALS1

MAGE Family Member A1 MAGEA1

CD4 Molecule CD4

Epoxide Hydrolase 1 EPHX1

Platelet And Endothelial Cell Adhesion Molecule 1 PECAM1

X Inactive Specific Transcript XIST

Matrix Metallopeptidase 13 MMP13

MicroRNA 138-1 MIR138-1

MutS Homolog 6 MSH6

Eukaryotic Translation Initiation Factor 4E EIF4E

Angiopoietin 1 ANGPT1

Nuclear Paraspeckle Assembly Transcript 1 NEAT1

C-X-C Motif Chemokine Receptor 2 CXCR2

Early Growth Response 1 EGR1

Aurora Kinase B AURKB

Matrix Metallopeptidase 12 MMP12

Reversion Inducing Cysteine Rich Protein With Kazal Motifs RECK

Gelsolin GSN

Apoptotic Peptidase Activating Factor 1 APAF1

Suppressor Of Cytokine Signaling 3 SOCS3

Cyclin D3 CCND3

Programmed Cell Death 4 PDCD4

Integrin Subunit Alpha V ITGAV

Dual Specificity Phosphatase 1 DUSP1

ATP Binding Cassette Subfamily C Member 3 ABCC3

MAGE Family Member A4 MAGEA4

Heparin Binding EGF Like Growth Factor HBEGF

MicroRNA 199a-1 MIR199A1

Urothelial Cancer Associated 1 UCA1

MicroRNA 139 MIR139

Caspase 7 CASP7

Epithelial Cell Transforming 2 ECT2

Phosphoinositide-3-Kinase Regulatory Subunit 3 PIK3R3

E2F Transcription Factor 2 E2F2

Baculoviral IAP Repeat Containing 7 BIRC7

MicroRNA 497 MIR497

AFAP1 Antisense RNA 1 AFAP1-AS1

Integrin Linked Kinase ILK

Claudin 7 CLDN7

Heparin Binding Growth Factor HDGF

MicroRNA 125b-1 MIR125B1

Colon Cancer Associated Transcript 1 CCAT1

Interleukin 24 IL24

Somatostatin Receptor 2 SSTR2

Ribonucleotide Reductase Regulatory Subunit M2 RRM2

RNA Binding Motif Protein 6 RBM6

Protein Kinase C Beta PRKCB

Macrophage Stimulating 1 Receptor MST1R

MicroRNA 196a-1 MIR196A1

Laminin Subunit Gamma 2 LAMC2

Nanog Homeobox NANOG

WNT Inhibitory Factor 1 WIF1

Baculoviral IAP Repeat Containing 2 BIRC2

Methylenetetrahydrofolate Reductase MTHFR

"MYCL Proto-Oncogene, BHLH Transcription Factor" MYCL

Protein Tyrosine Kinase 2 Beta PTK2B

Rho Associated Coiled-Coil Containing Protein Kinase 1 ROCK1

CDKN2B Antisense RNA 1 CDKN2B-AS1

Growth Differentiation Factor 15GDF15

Spermidine/Spermine N1-Acetyltransferase 1 SAT1

MicroRNA 338 MIR338

Phospholipase C Gamma 1 PLCG1

Erythrocyte Membrane Protein Band 4.1 Like 3 EPB41L3

Colon Cancer Associated Transcript 2 CCAT2

Small Nucleolar RNA Host Gene 1 SNHG1

BAG Cochaperone 1 BAG1

Placental Growth Factor PGF

Matrix Metallopeptidase 11 MMP11

X-Ray Repair Cross Complementing 6 XRCC6

MicroRNA 186 MIR186

"Alanyl Aminopeptidase, Membrane" ANPEP

Suppressor Of Cytokine Signaling 1 SOCS1

X-Ray Repair Cross Complementing 5 XRCC5

Gastrin Releasing Peptide Receptor GRPR

Cyclin D Binding Myb Like Transcription Factor 1 DMTF1

"Tumor Suppressor 2, Mitochondrial Calcium Regulator" TUSC2

Cell Division Cycle 25C CDC25C

Secretoglobin Family 1A Member 1 SCGB1A1

C-X-C Motif Chemokine Ligand 5 CXCL5

Cytidine Deaminase CDA

Cellular Communication Network Factor 1 CCN1

MAGE Family Member C2 MAGEC2

Thymidine Kinase 1 TK1

C-C Motif Chemokine Ligand 2 CCL2

CEA Cell Adhesion Molecule 1 CEACAM1

CD24 Molecule CD24

MicroRNA 124-3 MIR124-3

Inhibitor Of Nuclear Factor Kappa B Kinase Subunit Beta IKBKB

MicroRNA 30c-1 MIR30C1

Cathepsin L CTSL

Ras Homolog Family Member B RHOB

Nibrin NBN

MicroRNA 101-1 MIR101-1

Solute Carrier Family 29 Member 1 (Augustine Blood Group) SLC29A1

Cancer Susceptibility 2 CASC2

Ectonucleotide Pyrophosphatase/Phosphodiesterase 2 ENPP2

MicroRNA 361 MIR361

N-Acetyltransferase 1 NAT1

CD9 Molecule CD9

MicroRNA 149 MIR149

Notch Receptor 2 NOTCH2

Serpin Family B Member 3 SERPINB3

Serine/Threonine Kinase 4 STK4

Aldo-Keto Reductase Family 1 Member C1 AKR1C1

Arginyl-TRNA Synthetase 1 RARS1

Mesothelin MSLN

TNF Receptor Associated Factor 2 TRAF2

Cell Division Cycle 25A CDC25A

CEA Cell Adhesion Molecule 7 CEACAM7

Cyclin E2 CCNE2

"Aldolase, Fructose-Bisphosphate A" ALDOA

Epiregulin EREG

Growth Arrest And DNA Damage Inducible Gamma GADD45G

Solute Carrier Family 19 Member 1 SLC19A1

LDL Receptor Related Protein 1B LRP1B

Aldo-Keto Reductase Family 1 Member B10 AKR1B10

Integrin Subunit Alpha 6 ITGA6

MicroRNA 135a-1 MIR135A1

Cyclin G1 CCNG1

MicroRNA 24-1 MIR24-1

Neurotrophic Receptor Tyrosine Kinase 2 NTRK2

Prostaglandin E Receptor 4 PTGER4

MicroRNA 340 MIR340

Growth Arrest And DNA Damage Inducible Beta GADD45B

"Signal Peptide, CUB Domain And EGF Like Domain Containing 3" SCUBE3

"Long Intergenic Non-Protein Coding RNA, Regulator Of Reprogramming" LINC-ROR

Component Of Inhibitor Of Nuclear Factor Kappa B Kinase Complex CHUK

G Protein-Coupled Receptor Class C Group 5 Member A GPRC5A

Peroxiredoxin 1 PRDX1

Heterogeneous Nuclear Ribonucleoprotein A2/B1 HNRNPA2B1

MicroRNA 330 MIR330

MicroRNA 23b MIR23B

MicroRNA 133a-1 MIR133A1

Matrix Metallopeptidase 10 MMP10

Dickkopf WNT Signaling Pathway Inhibitor 3 DKK3

Prostaglandin E Synthase PTGES

HOXA Distal Transcript Antisense RNA HOTTIP

MicroRNA 206 MIR206

Tissue Factor Pathway Inhibitor 2 TFPI2

Sonic Hedgehog Signaling Molecule SHH

MicroRNA 574 MIR574

Receptor For Activated C Kinase 1 RACK1

CD55 Molecule (Cromer Blood Group) CD55

RNA Binding Motif Protein 5 RBM5

Breast Cancer Anti-Estrogen Resistance 4 BCAR4

Recoverin RCVRN

FOXF1 Adjacent Non-Coding Developmental Regulatory RNA FENDRR

Eukaryotic Translation Initiation Factor 2 Alpha Kinase 2 EIF2AK2

Cholecystokinin B Receptor CCKBR

Long Intergenic Non-Protein Coding RNA 673 LINC00673

Dihydrodiol Dehydrogenase DHDH

Ras Association Domain Family Member 5 RASSF5

Transcriptional And Immune Response Regulator TCIM

HOXA11 Antisense RNA HOXA11-AS

Adrenoceptor Beta 2 ADRB2

Deleted In Malignant Brain Tumors 1 DMBT1

Integrin Subunit Alpha 5 ITGA5

Myeloperoxidase MPO

MicroRNA 503 MIR503

TNF Receptor Associated Factor 6TRAF6

MicroRNA 218-2MIR218-2

Laminin Subunit Alpha 5 LAMA5

MicroRNA 212 MIR212

AT-Rich Interaction Domain 1A ARID1A

Apoptosis Inhibitor 5 API5

Prostate Cancer Associated Transcript 1 PCAT1

Gap Junction Protein Beta 5 GJB5

MicroRNA 542 MIR542

MicroRNA 27b MIR27B

CF Transmembrane Conductance Regulator CFTR

RalA Binding Protein 1 RALBP1

Bone Morphogenetic Protein 6 BMP6

H2A Clustered Histone 18 H2AC18

Tumor Suppressor Candidate 1 TUSC1

"NPR2 Like, GATOR1 Complex Subunit" NPRL2

C-Reactive Protein CRP

MicroRNA 125b-2 MIR125B2

HOXD Antisense Growth-Associated Long Non-Coding RNA HAGLR

Ribosomal Oxygenase 2 RIOX2

MicroRNA 339 MIR339

Tumor Protein P53 Pathway Corepressor 1 TP53COR1

TNF Receptor Associated Factor 1 TRAF1

Bladder Cancer Associated Transcript 1 BLACAT1

MicroRNA 154 MIR154

Glypican 3 GPC3

Differentiation Antagonizing Non-Protein Coding RNA DANCR

"PMS1 Homolog 2, Mismatch Repair System Component" PMS2

Peptidylprolyl Isomerase E Like Pseudogene PPIEL

ADAM Metallopeptidase Domain 12 ADAM12

HOP Homeobox HOPX

Serum Amyloid A1 SAA1

MIR4435-2 Host Gene MIR4435-2HG

HNF1A Antisense RNA 1 HNF1A-AS1

Small Nucleolar RNA Host Gene 16 SNHG16

Small Nucleolar RNA Host Gene 15 SNHG15

Neuromedin B NMB

Autocrine Motility Factor Receptor AMFR

MicroRNA 455 MIR455

Gastric Carcinoma Proliferation Enhancing Transcript 1 GHET1

MicroRNA 625 MIR625

Intercellular Adhesion Molecule 1 ICAM1

SPRY4 Intronic Transcript 1 SPRY4-IT1

Cholinergic Receptor Nicotinic Beta 4 Subunit CHRNB4

Hyaluronidase 2 HYAL2

TP73 Antisense RNA 1 TP73-AS1

Integrin Subunit Alpha 11 ITGA11

Small Nucleolar RNA Host Gene 20 SNHG20

Collagen Type IV Alpha 3 Chain COL4A3

ELAV Like RNA Binding Protein 4 ELAVL4

Promoter Of CDKN1A Antisense DNA Damage Activated RNA PANDAR

Roundabout Guidance Receptor 1 ROBO1

SOX2 Overlapping Transcript SOX2-OT

BRAF-Activated Non-Protein Coding RNA BANCR

MicroRNA 219a-1 MIR219A1

Neurotrophic Receptor Tyrosine Kinase 3 NTRK3

MicroRNA 103a-1 MIR103A1

Neuromedin B Receptor NMBR

Inducible T Cell Costimulator Ligand ICOSLG

MIR31 Host Gene MIR31HG

Tumor Protein P53 Binding Protein 2 TP53BP2

CEA Cell Adhesion Molecule 6 CEACAM6

Transcription Factor 7 TCF7

Bromodomain Testis Associated BRDT

F2R Like Thrombin Or Trypsin Receptor 3 F2RL3

MicroRNA 95 MIR95

TNF Receptor Associated Factor 4 TRAF4

Lysine Demethylase 4C KDM4C

Autophagy Related 7 ATG7

Long Intergenic Non-Protein Coding RNA 1133 LINC01133

Seizure Related 6 Homolog Like 2 SEZ6L2

MicroRNA 582 MIR582

Long Intergenic Non-Protein Coding RNA 511 LINC00511

Glutathione S-Transferase Theta 1 GSTT1

Timeless Circadian Regulator TIMELESS

Cytochrome P450 Family 2 Subfamily A Member 13 CYP2A13

ZNFX1 Antisense RNA 1 ZFAS1

TINCR Ubiquitin Domain Containing TINCR

Small Nucleolar RNA Host Gene 12 SNHG12

Laminin Subunit Alpha 4 LAMA4

NF-KappaB Interacting LncRNA NKILA

Ribosomal Protein SA RPSA

Tumor Suppressor Candidate 7 TUSC7

Vimentin VIM

Long Intergenic Non-Protein Coding RNA 261 LINC00261

MicroRNA 423 MIR423

Lung Cancer Associated Transcript 1 LUCAT1

Iron Responsive Element Binding Protein 2 IREB2

Sperm Associated Antigen 9 SPAG9

MicroRNA 708 MIR708

Myosin XVIIIB MYO18B

MicroRNA 216a MIR216A

CUB Domain Containing Protein 1 CDCP1

Interleukin 13 IL13

MicroRNA Let-7a-2 MIRLET7A2

FEZF1 Antisense RNA 1 FEZF1-AS1

Lymphocyte Antigen 6 Family Member K LY6K

Coactosin Like F-Actin Binding Protein 1 COTL1

Integrin Subunit Alpha 2 ITGA2

RGMB Antisense RNA 1 RGMB-AS1

Long Intergenic Non-Protein Coding RNA 312 LINC00312

Long Intergenic Non-Protein Coding RNA 968 LINC00968

Prostate Cancer Associated Transcript 6 PCAT6

MicroRNA 129-1 MIR129-1

Aminoacyl TRNA Synthetase Complex Interacting Multifunctional Protein 2 AIMP2

FOXD2 Adjacent Opposite Strand RNA 1 FOXD2-AS1

Gastric Cancer Associated Transcript 2 GACAT2

Leptin LEP

Serpin Family E Member 1 SERPINE1

Metadherin MTDH

Vitamin D Receptor VDR

Chromosome 20 Open Reading Frame 85 C20orf85

Homeobox B9 HOXB9

Dual Specificity Phosphatase 6 DUSP6

RAS P21 Protein Activator 1 RASA1

Long Intergenic Non-Protein Coding RNA 1852 LINC01852

Semaphorin 3F SEMA3F

Prostate Cancer Associated Transcript 7 PCAT7

Small Regulatory Polypeptide Of Amino Acid Response SPAAR

Titin TTN

NOVA Alternative Splicing Regulator 1 NOVA1

Cancer/Testis Antigen 83 CT83

RNA Binding Motif Protein 38 RBM38

MiR-17-92a-1 Cluster Host Gene MIR17HG

ASAP1 Intronic Transcript 1 ASAP1-IT1

Androgen Receptor AR

AGAP2 Antisense RNA 1 AGAP2-AS1

Laminin Subunit Gamma 1 LAMC1

Long Intergenic Non-Protein Coding RNA 858 LINC00858

TRPM2 Antisense RNA TRPM2-AS

Glyceraldehyde-3-Phosphate Dehydrogenase GAPDH

Calcium Voltage-Gated Channel Auxiliary Subunit Alpha2delta 2 CACNA2D2

Bombesin Receptor Subtype 3 BRS3

Angiotensin I Converting Enzyme ACE

PCBP2 Overlapping Transcript 1 PCBP2-OT1

SBF2 Antisense RNA 1 SBF2-AS1

CEA Cell Adhesion Molecule 3 CEACAM3

P53 Regulation Associated LncRNAPRAL

Cytochrome P450 Family 19 Subfamily A Member 1 CYP19A1

Lysine Methyltransferase 2D KMT2D

Schlafen Family Member 11 SLFN11

MicroRNA 151a MIR151A

Endoglin ENG

Interleukin 17A   IL17A

EPH Receptor A7   EPHA7

TNF Receptor Associated Factor 3TRAF3

Interleukin 1 Receptor Antagonist   IL1RN

BRCA1 Associated RING Domain 1 BARD1

IST1 Factor Associated With ESCRT-III IST1

Long Intergenic Non-Protein Coding RNA 1433   LINC01433

Tubulin Alpha 4b TUBA4B

TatD DNase Domain Containing 1 TATDN1

SRY-Box Transcription Factor 30 SOX30

MicroRNA 28   MIR28

"DNA Polymerase Epsilon, Catalytic Subunit"   POLE

KCNMB2 Antisense RNA 1   KCNMB2-AS1

DiGeorge Syndrome Critical Region Gene 5   DGCR5

Brain Cytoplasmic RNA 1 BCYRN1

Ubiquitin Like Modifier Activating Enzyme 7   UBA7

Long Intergenic Non-Protein Coding RNA 460 LINC00460

Collagen Type IV Alpha 2 Chain COL4A2

Keratin 5 KRT5

Lung Cancer Associated LncRNA 1 LCAL1

Cancer Susceptibility 8 CASC8

TP53 Target 1 TP53TG1

SLC16A1 Antisense RNA 1 SLC16A1-AS1

MicroRNA 532 MIR532

MicroRNA 130b MIR130B

Ribosomal Oxygenase 1 RIOX1

Dihydrouridine Synthase 2 DUS2

Long Noncoding RNA Activated By TGF-Beta LNCRNA-ATB

Cytokine Inducible SH2 Containing Protein CISH

Podoplanin PDPN

MicroRNA Let-7f-1 MIRLET7F1

MicroRNA 345 MIR345

CD40 Ligand CD40LG

TLC Domain Containing 3A TLCD3A

Small Nucleolar RNA Host Gene 7 SNHG7

MicroRNA 425 MIR425

"Elastase, Neutrophil Expressed" ELANE

Interferon Alpha 1 IFNA1

Cancer Susceptibility 9 CASC9

IGF2 Antisense RNA IGF2-AS

Notch Receptor 3 NOTCH3

"Zinc Ribbon Domain Containing 1 Antisense, Pseudogene" ZNRD1ASP

MIR22 Host Gene MIR22HG

Aryl-Hydrocarbon Receptor Repressor AHRR

"RNA, Ro60-Associated Y1" RNY1

"RNA, Ro60-Associated Y3" RNY3

InsulinINS

RAD51 Paralog C RAD51C

Lysine Demethylase 1A KDM1A

MicroRNA 218-1 MIR218-1

Superoxide Dismutase 1 SOD1

GAS6 Antisense RNA 1 GAS6-AS1

Caudal Type Homeobox 2 CDX2

Uncharacterized LOC730101 LOC730101

CD8a Molecule CD8A

EPB41L4A Divergent Transcript EPB41L4A-DT

MAFA Antisense RNA 1 MAFA-AS1

Discoidin Domain Receptor Tyrosine Kinase 1 DDR1

Patched 1 PTCH1

Forkhead Box O1 FOXO1

IGF1R Antisense Imprinted Non-Protein Coding RNA IRAIN

DLG Associated Protein 2 DLGAP2

FAM83H Antisense RNA 1 (Head To Head) FAM83H-AS1

Holliday Junction Recognition Protein HJURP

F-Box And WD Repeat Domain Containing 7 FBXW7

Long Intergenic Non-Protein Coding RNA 1186 LINC01186

Nitric Oxide Synthase 3 NOS3

Long Intergenic Non-Protein Coding RNA 1627 LINC01627

Colony Stimulating Factor 1 Receptor CSF1R

Wnt Family Member 5A WNT5A

ZEB2 Antisense RNA 1 ZEB2-AS1

Long Intergenic Non-Protein Coding RNA 1116	LINC01116

Caspase 10 CASP10

Glial Fibrillary Acidic Protein GFAP

Cyclin Dependent Kinase Inhibitor 3 CDKN3

SMAD Family Member 7 SMAD7

"Adiponectin, C1Q And Collagen Domain Containing" ADIPOQ

RAD50 Double Strand Break Repair Protein	RAD50

ATR Serine/Threonine Kinase ATR

Tubulin Beta 3 Class III TUBB3

Endothelin 1 EDN1

HIF1A Antisense RNA 1 HIF1A-AS1

CPS1 Intronic Transcript 1	CPS1-IT1

ADAMTS9 Antisense RNA 2 ADAMTS9-AS2

Geranylgeranyl Diphosphate Synthase 1	GGPS1

Long Intergenic Non-Protein Coding RNA 313 LINC00313

WW Domain Containing Oxidoreductase WWOX

MicroRNA 511 MIR511

MicroRNA 376a-1	MIR376A1

Long Intergenic Non-Protein Coding RNA 857 LINC00857

Zinc Finger Protein 793 ZNF793

Chromodomain Helicase DNA Binding Protein 7	CHD7

Long Intergenic Non-Protein Coding RNA 473 LINC00473

NEXN Antisense RNA 1 NEXN-AS1

"Mucin 5B, Oligomeric Mucus/Gel-Forming"	MUC5B

KIT Ligand KITLG

Surfactant Protein A1 SFTPA1

Zinc Finger Protein 461 ZNF461

Catechol-O-Methyltransferase COMT

Mitogen-Activated Protein Kinase Kinase 4 MAP2K4

TSC Complex Subunit 1 TSC1

Mitosis Associated Long Intergenic Non-Coding RNA 1 MALINC1

Guanylate Cyclase 1 Soluble Subunit Beta 2 (Pseudogene) GUCY1B2

Long Intergenic Non-Protein Coding RNA 210 LINC00210

Long Intergenic Non-Protein Coding RNA 1589 LINC01589

GAS5 Antisense RNA 1 GAS5-AS1

"Major Histocompatibility Complex, Class I, A" HLA-A

G Protein Signaling Modulator 2 GPSM2

Chromosome 14 Open Reading Frame 132 C14orf132

Long Intergenic Non-Protein Coding RNA 880 LINC00880

SGO1 Antisense RNA 1 SGO1-AS1

"XPC Complex Subunit, DNA Damage Recognition And Repair Factor" XPC

"ABL Proto-Oncogene 1, Non-Receptor Tyrosine Kinase" ABL1

GNAS Antisense RNA 1 GNAS-AS1

DisGeNET database

Gene_Full_Name Gene Symbol

epidermal growth factor receptorEGFR

tumor protein p53 TP53

"ERCC excision repair 1, endonuclease non-catalytic subunit" ERCC1

colony stimulating factor 2 CSF2

"KRAS proto-oncogene, GTPase" KRAS

S100 calcium binding protein A1 S100A1

vascular endothelial growth factor A VEGFA

vitamin D receptor VDR

erb-b2 receptor tyrosine kinase 2 ERBB2

metastasis associated lung adenocarcinoma transcript 1 MALAT1

S100 calcium binding protein B S100B

voltage dependent anion channel 1 VDAC1

ribonucleotide reductase catalytic subunit M1 RRM1

prostaglandin D2 receptor PTGDR

C-X-C motif chemokine ligand 5 CXCL5

paired like homeodomain 2 PITX2

S100 calcium binding protein A2 S100A2

MHC class I polypeptide-related sequence A MICA

cytohesin 1 interacting protein CYTIP

surfactant protein A2 SFTPA2

microRNA 574 MIR574

surfactant protein A1 SFTPA1

microRNA 499a MIR499A

microRNA 374a MIR374A

microRNA 34b MIR34B

microRNA 221 MIR221

microRNA 200c MIR200C

microRNA 20a MIR20A

microRNA 141 MIR141

adherens junctions associated protein 1 AJAP1

integrator complex subunit 6 INTS6

cartilage associated protein CRTAP

receptor for activated C kinase 1 RACK1

tripartite motif containing 28 TRIM28

proliferating cell nuclear antigen PCNA

pyruvate carboxylase PC

choline kinase alpha CHKA

cysteine dioxygenase type 1 CDO1

cyclin dependent kinase inhibitor 2B CDKN2B

cyclin dependent kinase inhibitor 2A CDKN2A

cyclin dependent kinase 2 CDK2

CD33 molecule CD33

CD14 molecule CD14

cyclin D1 CCND1

Fas ligand FASLG

Fas cell surface death receptor FAS

baculoviral IAP repeat containing 5 BIRC5

ALK receptor tyrosine kinase ALK

aldehyde dehydrogenase 1 family member A1 ALDH1A1

cholinergic receptor nicotinic alpha 3 subunit CHRNA3

ATP binding cassette subfamily C member 2 ABCC2

"MYC proto-oncogene, bHLH transcription factor" MYC

MDM2 proto-oncogene MDM2

keratin 81 KRT81

integrin subunit alpha M ITGAM

eukaryotic translation initiation factor 3 subunit E EIF3E

homeobox A9 HOXA9

general transcription factor IIBGTF2B

fucosyltransferase 4 FUT4

fibroblast growth factor receptor 1 FGFR1

EPH receptor B6 EPHB6

cut like homeobox 1 CUX1

mitogen-activated protein kinase kinase kinase 8 MAP3K8

collagen type IV alpha 3 chain COL4A3

AKT serine/threonine kinase 1 AKT1
