# Supplementary material for: Anticancer Action of Xiaoxianxiong Tang in Non-Small Cell Lung Cancer by Pharmacological Analysis and Experimental Validation
Source: Evid Based Complement Alternat Med. 2021 Dec 13;2021:9930082. doi: 10.1155/2021/9930082 (PMC8687818; doi:10.1155/2021/9930082)
Supplement: Supplementary Materials — All primer sets in the RT-qPCR array are shown in the Table 1. Table 2 indicates the targets in XXXT. Table 3 indicates the targets related to NSCLC. Table 4 indicates common targets of NSCLC and XXXT. Table 5 indicates the result of the RT-qPCR array in H460 cells. Table 5 indicates the result of the RT-qPCR array in A549 cells. [file 9930082.f1.zip › 9930082.f1/Supplementary table 4-Common targets of NSCLC and XXXT.pdf]

| XXXT | NSCLC | Common targets |
|---|---|---|
| NOS2 | EGFR | ABCG2 |
| PTGS1 | TP53 | ACACA |
| KCNH2 | ERCC1 | ACHE |
| ESR1 | CSF2 | ADH1C |
| AR | KRAS | ADRB1 |
| SCN5A | S100A1 | ADRB2 |
| PTGS2 | VEGFA | AHR |
| NOS3 | VDR | AKT1 |
| PRSS1 | ERBB2 | ALOX12 |
| RXRA | MALAT1 | ALOX5 |
| NCOA2 | S100B | AR |
| CHRM3 | VDAC1 | BAX |
| F10 | RRM1 | BCL2 |
| F7 | PTGDR | BCL2L1 |
| ADRA1B | CXCL5 | BIRC5 |
| ADRB2 | PITX2 | CA2 |
| ADRA1D | S100A2 | CASP3 |
| HSP90AB1 | MICA | CASP8 |
| KCNMA1 | CYTIP | CASP9 |
| PDE10A | SFTPA2 | CAV1 |
| CHEK1 | MIR574 | CCL2 |
| PIM1 | SFTPA1 | CCNA2 |
| PPARG | MIR499A | CCNB1 |

| | | |
|---|---|---|
| PIK3CG | MIR374A | CCND1 |
| DPP4 | MIR34B | CD40LG |
| TOP2 | MIR221 | CDK1 |
| MMP3 | MIR200C | CDK2 |
| ACHE | MIR20A | CDKN1A |
| GABRA1 | MIR141 | CHEK1 |
| MAOB | AJAP1 | CHEK2 |
| RELA | INTS6 | CHRM2 |
| EGFR | CRTAP | CHRM3 |
| AKT1 | RACK1 | CHRNA7 |
| VEGFA | TRIM28 | CHUK |
| CCND1 | PCNA | CLDN4 |
| BCL2 | PC | COL1A1 |
| BCL2L1 | CHKA | COL8A1 |
| FOS | CDO1 | CRP |
| CDKN1A | CDKN2B | CTSD |
| EIF6 | CDKN2A | CXCL10 |
| BAX | CDK2 | CXCL8 |
| CASP9 | CD33 | CYCS |
| PLAU | CD14 | CYP1A1 |
| MMP2 | CCND1 | CYP1A2 |
| MMP9 | FASLG | CYP1B1 |
| MAPK1 | FAS | CYP3A4 |
| IL10 | BIRC5 | DPP4 |

| | | |
|---|---|---|
| EGF | ALK | E2F1 |
| RB1 | ALDH1A1 | E2F2 |
| TNF | CHRNA3 | EGF |
| JUN | ABCC2 | EGFR |
| IL6 | MYC | EGLN1 |
| AHSA1 | MDM2 | EIF6 |
| CASP3 | KRT81 | ELK1 |
| TP53 | ITGAM | ERBB2 |
| ELK1 | EIF3E | ERBB3 |
| NFKBIA | HOXA9 | ESR1 |
| POR | GTF2B | ESR2 |
| ODC1 | FUT4 | F10 |
| XDH | FGFR1 | F3 |
| CASP8 | EPHB6 | FABP5 |
| TOP1 | CUX1 | FOS |
| RAF1 | MAP3K8 | FOSL1 |
| SOD1 | COL4A3 | FOSL2 |
| PRKCA | AKT1 | GABRA3 |
| MMP1 | BRAF | GJA1 |
| HIF1A | PIK3CA | GSTM1 |
| STAT1 | PTEN | GSTM2 |
| RUNX1T1 | BRCA2 | GSTP1 |
| CDK1 | STK11 | HIF1A |
| HSPA5 | BRCA1 | HK2 |

| | | |
|---|---|---|
| ERBB2 | CDH1 | HMOX1 |
| ACACA | MLH1 | HSF1 |
| HMOX1 | MET | HSP90AB1 |
| CYP3A4 | IL6 | HSPA5 |
| CYP1A2 | APC | HSPB1 |
| CAV1 | STAT3 | HTR3A |
| MYC | ATM | ICAM1 |
| F3 | NRAS | IFNG |
| GJA1 | HRAS | IGF2 |
| CYP1A1 | TGFB1 | IGFBP3 |
| ICAM1 | TERT | IL10 |
| IL1B | CTNNB1 | IL1A |
| CCL2 | RB1 | IL1B |
| SELE | TNF | IL2 |
| VCAM1 | CDKN1A | IL6 |
| PTGER3 | NOTCH1 | INSR |
| CXCL8 | EGF | IRF1 |
| PRKCB | ERCC2 | JUN |
| BIRC5 | BAX | KCNMA1 |
| DUOX2 | CDK4 | LTA4H |
| HSPB1 | MAP2K1 | MAOA |
| TGFB1 | NKX2-1 | MAP2 |
| SULT1E1 | MTOR | MAPK1 |
| MGAM | CHEK2 | MMP1 |

| | | |
|---|---|---|
| IL2 | ESR1 | MMP2 |
| NR1I2 | SMAD4 | MMP3 |
| CYP1B1 | FHIT | MMP9 |
| CCNB1 | RARB | MPO |
| PLAT | MIR21 | MYC |
| THBD | KIT | NCOA2 |
| SERPINE1 | IL10 | NFATC1 |
| COL1A1 | DDR2 | NFE2L2 |
| IFNG | CASP8 | NFKBIA |
| ALOX5 | CDKN1B | NOS2 |
| PTEN | TP63 | NOS3 |
| IL1A | TYMS | NQO1 |
| MPO | CXCR4 | NR1I2 |
| TOP2A | ROS1 | NR3C1 |
| NCF1 | RET | NR3C2 |
| ABCG2 | MGMT | ODC1 |
| HAS2 | SRC | OPRM1 |
| GSTP1 | TGFBR2 | PDE3A |
| NFE2L2 | NFE2L2 | PGR |
| NQO1 | AKT2 | PIK3CG |
| TNKS | SPP1 | PIM1 |
| AHR | NFKB1 | PLAT |
| PSMD3 | CD274 | PLAU |
| SLC2A4 | PDCD1 | PON1 |

| | | |
|---|---|---|
| COL8A1 | CAV1 | POR |
| CXCL11 | FGFR2 | PPARA |
| CXCL2 | ERBB3 | PPARD |
| DCAF5 | EZH2 | PPARG |
| NR1I3 | ESR2 | PRKCA |
| CHEK2 | BAP1 | PRKCB |
| INSR | RAF1 | PRSS1 |
| CLDN4 | PPARG | PSMD3 |
| PPARA | BCL2 | PTEN |
| PPARD | FGFR3 | PTGER3 |
| HSF1 | MIR145 | PTGS1 |
| CRP | PLAU | PTGS2 |
| CXCL10 | TYMP | PTPN1 |
| CHUK | SOX2 | RAF1 |
| SPP1 | ABCB1 | RASA1 |
| RUNX2 | PDGFRB | RASSF1 |
| RASSF1 | MMP1 | RB1 |
| E2F1 | SLC2A1 | RELA |
| E2F2 | CXCL12 | RUNX1T1 |
| ACP3 | PTGS2 | RUNX2 |
| CTSD | RAD51 | RXRA |
| IGFBP3 | MMP9 | RXRB |
| IGF2 | FLT1 | SELE |
| CD40LG | XIAP | SERPINE1 |

| | | |
|---|---|---|
| IRF1 | TNFRSF10B | SLC6A3 |
| ERBB3 | SNAI2 | SOD1 |
| PON1 | SMARCA4 | SPP1 |
| DIO1 | TOP2A | STAT1 |
| PCOLCE | TLR4 | TGFB1 |
| NPEPPS | TERC | THBD |
| HK2 | MIR17 | TNF |
| NKX3-1 | PIK3R1 | TNKS |
| RASA1 | XRCC1 | TOP1 |
| GSTM1 | ZEB1 | TOP2A |
| GSTM2 | DLEC1 | TP53 |
| CHRM1 | EPCAM | VEGFA |
| CHRM5 | EP300 | XDH |
| HTR3A | MIR126 | |
| ADRA2C | TP73 | |
| CHRM4 | MIR34A | |
| OPRD1 | DNMT1 | |
| HTR2A | H19 | |
| HTR2C | IGF2 | |
| SLC6A3 | ERBB4 | |
| SLC6A4 | RELA | |
| OPRM1 | PDGFRA | |
| DRD1 | OGG1 | |
| DRD5 | MAPK1 | |

SLC6A2 SMAD3

ADRA1A STAT1

CHRM2 PROM1

GABRA2 GLI1

GRIA2 FOXP3

GABRA6 CYP1B1

ESR2 HSPB1

CDK2 MIR155

NR3C2 MIR125A

PGR JAK2

ADRB1 TWIST1

RXRB AURKA

PRKACA CTLA4

PDE3A MIR143

NCOA1 MIR205

FOSL1 DPYD

FOSL2 AXIN2

CYCS MEG3

ALOX12 MIR146A

NFATC1 XRCC3

TDRD7 MSH3

EGLN1 NOS2

NOX5 CSF3

FABP5 MMP2

| | |
|---|---|
| APOD | AKT3 |
| PTPN1 | CCR6 |
| GABRA5 | MIR31 |
| GABRB3 | CASP3 |
| CHRNA2 | MIR150 |
| CHRNA7 | ARAF |
| camC | GSTP1 |
| MAP2 | MIR142 |
| ADH1C | CREB1 |
| ADRA2A | IRF1 |
| AKR1B1 | MIR182 |
| LTA4H | MIR214 |
| MAOA | HIF1A |
| CTRB1 | AHR |
| GABRA3 | MIR148A |
| CA2 | RASSF1 |
| CCNA2 | SLC22A18 |
| PNP | VHL |
| NR3C1 | PDGFB |
| | MIR200A |
| | MIR200B |
| | MIR193A |
| | BIRC3 |
| | MIRLET7A1 |

RARA

CXCL8

MIR29A

CHEK1

BMP2

MIR27A

EGFR-AS1

CEACAM5

XPA

MIR25

MIR140

MIR29C

DNMT3A

NTRK1

MIR183

MIR195

IGF1R

MIR15A

MIR144

MUC1

NFKBIA

GJA1

SETD2

MIR203A

CHRNA5

MIR223

MYCN

CYP2A6

ERCC5

TNFRSF1A

MIR222

ZEB2

MAP2K2

ZMYND10

MIR181A1

KLF6

ITGA3

ERCC6

MXRA5

HPGD

GSTM1

IRS1

STAT5B

MIR96

PIK3R2

MIR486-1

KRT19

CYP2D6

MIR210

MIR106B

MIR16-1

MIR99A

HMGA2

MIR204

MIR30E

ODC1

COL18A1

UGT1A1

MTAP

MIRLET7B

PRKN

MIR18A

MIR93

MIR26A1

KDR

MIRLET7D

ENO2

MIR34C

TFAP2A

B2M

MIR107

MIR22

MIR335

MIR23A

MIR424

VEGFC

CD40

MIR100

PPP2R1B

MIR451A

HLA-G

CYP1A1

BUB1

GATA2

ERCC4

MIR193B

MIR32

MIR373

MIR137

ABCC1

MIR133B

MIR224

JAK3

KCNQ1OT1

SOS1

MIR185

EML4

MIR19A

MIR148B

U2AF1

CASP9

MIR483

JUN

TRIP13

DDB2

MIR106A

MIR296

IL4R

MIR30B

MIRLET7E

TOP1

MIAT

POLK

CD44

MIR199B

MIR98

BCL2L1

E2F1

MIR192

HAVCR2

MAPK3

MIR378A

MIR146B

MIR342

MIR30D

TGFA

MAPK8

MIR130A

GRP

FGF2

TNFSF10

HGF

MIRLET7G

MIR15B

IGF1

MIRLET7C

MIR191

ASCL1

MIR29B2

MKI67

MIR331

NQO1

MIR10A

MIR324

ABCG2

LAMB3

MIR429

MIR377

MIR128-2

MIR33A

MIR197

MAD1L1

MIR372

MAX

PARP1

MIR198

MIRLET7I

ACTA2

PTK2

TAP1

DLK1

IL2

NF1

MCL1

MSH2

MIRLET7A3

ITGA2B

MIR24-2

CYCS

TIMP1

MMP7

CHGA

TGFBR1

FLT4

PRKCI

ALB

IGFBP3

MVP

YWHAE

CCNB1

BAK1

IL4

TSC2

KEAP1

PRKCA

MUC16

MAPK14

PLAUR

RMRP

DAPK1

SYP

CCNA2

SEMA3B

SNAI1

PGR

SP1

CADM1

CDK6

BCL2L11

HSP90AA1

KRT7

CDK1

NME1

TKT

APEX1

RAC1

AREG

MIR181C

FGFR4

TIMP2

WT1

YAP1

ITGB1

SOD2

FOXM1

SKP2

KRT18

FN1

MIR30A

HOTAIR

HMGB1

HMOX1

PIK3CG

POU5F1

SFTPC

BAD

FOS

SERPINA1

NRG1

DICER1

STMN1

ANGPT2

HDAC1

PLK1

CFLAR

IFI27

CA9

IL1B

S100A4

ANXA5

THBS1

MMP14

RHOA

TIMP3

PTPN11

GADD45A

BMP4

CCN2

BMI1

CDH13

AXL

CAT

CTAG1B

VEGFD

SFTPD

SFTPB

SERPINB5

HSPA5

RXRG

HDAC9

TNFRSF10A

RUNX3

FOXO3

BSG

RPS6KB1

CYP2E1

TUBB

DNMT3B

NCAM1

CDH2

MMP3

MUC5AC

LGALS3

MIF

ENO1

POSTN

PVT1

DHFR

EPHA2

E2F3

SMAD2

NRP1

BECN1

SPARC

CYP3A4

IFNG

HSPA4

CTSB

FSCN1

GNAS

MIR375

GAS5

KRT8

TXN

CTNND1

IL3

TLR9

DCK

FASN

PIK3CB

SERPINA3

JUP

IDH1

KRT20

MIR124-1

BPIFA1

MUC4

PTHLH

UCHL1

CCR7

DIABLO

LOX

EZR

GRB2

CD82

RXRA

MAGEA3

MIR9-1

YBX1

NAT2

PDPK1

PXN

CCNE1

TUG1

NAPSA

CXCL1

CDC42

LGALS1

MAGEA1

CD4

EPHX1

PECAM1

XIST

MMP13

MIR138-1

MSH6

EIF4E

ANGPT1

NEAT1

CXCR2

EGR1

AURKB

MMP12

RECK

GSN

APAF1

SOCS3

CCND3

PDCD4

ITGAV

DUSP1

ABCC3

MAGEA4

HBEGF

MIR199A1

UCA1

MIR139

CASP7

ECT2

PIK3R3

E2F2

BIRC7

MIR497

AFAP1-AS1

ILK

CLDN7

HDGF

MIR125B1

CCAT1

IL24

SSTR2

RRM2

RBM6

PRKCB

MST1R

MIR196A1

LAMC2

NANOG

WIF1

BIRC2

MTHFR

MYCL

PTK2B

ROCK1

CDKN2B-AS1

GDF15

SAT1

MIR338

PLCG1

EPB41L3

CCAT2

SNHG1

BAG1

PGF

MMP11

XRCC6

MIR186

ANPEP

SOCS1

XRCC5

GRPR

DMTF1

TUSC2

CDC25C

SCGB1A1

CDA

CCN1

MAGEC2

TK1

CCL2

CEACAM1

CD24

MIR124-3

IKBKB

MIR30C1

CTSL

RHOB

NBN

MIR101-1

SLC29A1

CASC2

ENPP2

MIR361

NAT1

CD9

MIR149

NOTCH2

SERPINB3

STK4

AKR1C1

RARS1

MSLN

TRAF2

CDC25A

CEACAM7

CCNE2

ALDOA

EREG

GADD45G

SLC19A1

LRP1B

AKR1B10

ITGA6

MIR135A1

CCNG1

MIR24-1

NTRK2

PTGER4

MIR340

GADD45B

SCUBE3

LINC-ROR

CHUK

GPRC5A

PRDX1

HNRNPA2B1

MIR330

MIR23B

MIR133A1

MMP10

DKK3

PTGES

HOTTIP

MIR206

TFPI2

SHH

CD55

RBM5

BCAR4

RCVRN

FENDRR

EIF2AK2

CCKBR

LINC00673

DHDH

RASSF5

TCIM

HOXA11-AS

ADRB2

DMBT1

ITGA5

MPO

MIR503

TRAF6

MIR218-2

LAMA5

MIR212

ARID1A

API5

PCAT1

GJB5

MIR542

MIR27B

CFTR

RALBP1

BMP6

H2AC18

TUSC1

NPRL2

CRP

MIR125B2

HAGLR

RIOX2

MIR339

TP53COR1

TRAF1

BLACAT1

MIR154

GPC3

DANCR

PMS2

PPIEL

ADAM12

HOPX

SAA1

MIR4435-2HG

HNF1A-AS1

SNHG16

SNHG15

NMB

AMFR

MIR455

GHET1

MIR625

ICAM1

SPRY4-IT1

CHRNB4

HYAL2

TP73-AS1

ITGA11

SNHG20

ELAVL4

PANDAR

ROBO1

SOX2-OT

BANCR

MIR219A1

NTRK3

MIR103A1

NMBR

ICOSLG

MIR31HG

TP53BP2

CEACAM6

TCF7

BRDT

F2RL3

MIR95

TRAF4

KDM4C

ATG7

LINC01133

SEZ6L2

MIR582

LINC00511

GSTT1

TIMELESS

CYP2A13

ZFAS1

TINCR

SNHG12

LAMA4

NKILA

RPSA

TUSC7

VIM

LINC00261

MIR423

LUCAT1

IREB2

SPAG9

MIR708

MYO18B

MIR216A

CDCP1

IL13

MIRLET7A2

FEZF1-AS1

LY6K

COTL1

ITGA2

RGMB-AS1

LINC00312

LINC00968

PCAT6

MIR129-1

AIMP2

FOXD2-AS1

GACAT2

LEP

SERPINE1

MTDH

C20orf85

HOXB9

DUSP6

RASA1

LINC01852

SEMA3F

PCAT7

SPAAR

TTN

NOVA1

CT83

RBM38

MIR17HG

ASAP1-IT1

AR

AGAP2-AS1

LAMC1

LINC00858

TRPM2-AS

GAPDH

CACNA2D2

BRS3

ACE

PCBP2-OT1

SBF2-AS1

CEACAM3

PRAL

CYP19A1

KMT2D

SLFN11

MIR151A

ENG

IL17A

EPHA7

TRAF3

IL1RN

BARD1

IST1

LINC01433

TUBA4B

TATDN1

SOX30

MIR28

POLE

KCNMB2-AS1

DGCR5

BCYRN1

UBA7

LINC00460

COL4A2

KRT5

LCAL1

CASC8

TP53TG1

SLC16A1-AS1

MIR532

MIR130B

RIOX1

DUS2

LNCRNA-ATB

CISH

PDPN

MIRLET7F1

MIR345

CD40LG

TLCD3A

SNHG7

MIR425

ELANE

IFNA1

CASC9

IGF2-AS

NOTCH3

ZNRD1ASP

MIR22HG

AHRR

RNY1

RNY3

INS

RAD51C

KDM1A

MIR218-1

SOD1

GAS6-AS1

CDX2

LOC730101

CD8A

EPB41L4A-DT

MAFA-AS1

DDR1

PTCH1

FOXO1

IRAIN

DLGAP2

FAM83H-AS1

HJURP

FBXW7

LINC01186

NOS3

LINC01627

CSF1R

WNT5A

ZEB2-AS1

LINC01116

CASP10

GFAP

CDKN3

SMAD7

ADIPOQ

RAD50

ATR

TUBB3

EDN1

HIF1A-AS1

CPS1-IT1

ADAMTS9-AS2

GGPS1

LINC00313

WWOX

MIR511

MIR376A1

LINC00857

ZNF793

CHD7

LINC00473

NEXN-AS1

MUC5B

KITLG

ZNF461

COMT

MAP2K4

TSC1

MALINC1

GUCY1B2

LINC00210

LINC01589

GAS5-AS1

HLA-A

GPSM2

C14orf132

LINC00880

SGO1-AS1

XPC

ABL1

GNAS-AS1

ENSG00000266919

MUC20-OT1

MYEF2

TCF7L2

ENSG00000249738

ENSG00000257337

CTNNA1

CACNA1G-AS1

IL2RA

LINC02412

ENSG00000255224

ENSG00000249592

ENSG00000285095

ENSG00000223859

LINC00342

TNFRSF1B

CTTN

LINC01502

LINC02042

ENSG00000279080

NSD1

GSK3B

WNT1

VWF

TGFB2

ANXA1

ELN

MUTYH

FGF9

MME

CALB2

HLA-DRB1

CCL3

MAPK9

SOX9

SMARCA2

TLR2

MAP3K7

MTA1

CD74

SIRT1

BDNF

CHFR

MIR432

VEGFB

IL1A

SOX4

USP7

IL7

CLPTM1L

F2

PLG

SST

PRKDC

PEBP1

F3

BMPR1A

KDM6A

HLA-B

EPHB4

ETS1

IDO1

LRRC56

ALOX5

EDNRA

H2AX

CYP17A1

ITGB3

MRE11

EPHA3

MIR135B

PKM

KLF4

BMPR2

EPOR

EPO

EIF2AK3

CLU

THBD

IL18

MEN1

CREBBP

MIR10B

NF2

CSF1

CTSK

IGF2BP3

DKK1

MAP2K7

CXCR1

CCND2

CD28

CLDN3

CCL5

PCSK9

IFNA2

MYLK

EPAS1

KLK3

CYP3A5

LNCR3

ANXA2

CALR

COL1A1

KIF5B

JAG1

CTSD

APOE

NCOR2

PTPN13

NCOR1

TBL1XR1

XAGE1A

LTA

IGF2R

PDCD1LG2

ATP2C1

DES

LIG4

HP

IGFBP2

MIR152

RUNX2

MIR485

MBL2

NAA80

NCOA3

CHI3L1

ID1

CBL

TLR5

GATA4

PTGS1

CALCA

WWTR1

MIR29B1

IL6R

MIR187

IL7R

PSG2

NR1H2

ELAVL1

FGF7

STAT6

EDNRB

PRDM5

PRKCD

CXCR3

NPM1

XRCC2

S100A7

CYP1A2

HDAC2

GREM1

HLA-DQB1

TF

LOXL2

CYTOR

GAST

HPSE

MSR1

AGTR1

NR3C1

PPM1D

TRIM24

XAGE1B

WRAP53

RXRB

PAX6

PML

MIR454

POMC

JAK1

GLI3

AFP

DLC1

ERG

AQP3

SMARCB1

ING1

HMGA1

HFE

MIR320A

TEK

CTAG2

SQSTM1

BID

CXCL10

CDKN2C

CD46

TLR3

EIF4EBP1

BUB1B

TGM2

SFTA1P

CA8

SELP

UBE2I

FGF1

THPO

SLC7A5

INSR

MIR409

AGER

F2R

MYD88

SERPINC1

PHB

GHRL

NGF

ADA

GRN

PTPRC

FOXA1

WNT3

TNFSF11

MIR181B1

PDGFRL

NES

PDGFA

CASP2

FANCD2

PLA2G2A

IL12B

CCL18

MIR122

PTGER3

ETS2

CD36

AXIN1

PRSS1

TPI1

SFRP1

DSP

IL5

ETV4

TNC

PAK1

COL11A1

MCM2

EPHB2

WNT2

TTR

VCP

BRMS1

TFRC

SELE

ACVRL1

IDH2

MIR134

MIR196A2

MIR132

IL1R1

VIP

ETV6

MIR671

KRT14

PIP

CDC6

SCGB2A2

CSNK2A1

NPTN-IT1

NFKB2

MIR328

DKC1

CEBPA

VTCN1

CLDN1

HOXA5

MIR127

HK2

TUSC3

MYH9

MLH3

PIN1

BCAR1

TNFRSF11B

CCDC6

AMACR

MUC3A

PLA2G4A

PRKCE

IGFBP7

CHRM3

AIFM1

F5

HABP2

MIR184

S100A6

PRKAA1

HLA-C

IL12A

CIP2A

PIM1

LPL

FOXF1

GUSB

STAT5A

GLI2

GPX1

CUL1

GZMB

TGFBI

LYVE1

PIEZO2

COX5A

IL15

LEPR

GATA3

CD80

CD34

MARS1

FOXC1

TPD52

FADD

RHOC

DNAH8

CCR2

NTS

CLDN5

CD276

LIMK1

LATS2

PON1

TFF1

S100A9

PEBP4

EBAG9

PPP1R13L

SLC34A2

CLDN4

ABRAXAS1

LLGL1

SERPINB2

HSD17B1

MIR9-3

ADAM17

SMPD1

PLCB1

MCC

FLNA

HDAC6

PTPRG

HPRT1

SHC1

PAX5

MACC1

PYGM

RAP1A

MIR873

SIRT6

FANCA

HSPD1

PRL

FOLR1

CDC25B

KISS1

HDAC4

MIR449A

NEU1

SYK

LCN2

MECP2

FUS

IL33

CR1

ACTC1

ACTN4

GATA6

SMAD6

NUS1

IKBKG

IL11

PCLAF

PRDX4

CD86

MAPT

CHRNA7

HIC1

TERF1

DDIT3

NOB1

ITGB4

MCM7

FYN

APOA1

SSX2

FANCG

SOS2

ERCC3

SGK1

WFDC2

TCF3

LIPC

NAMPT

LINC00472

CDKN1C

P2RX7

TDGF1

NDRG1

PTTG1

DCN

IRS2

ALDH2

CFL1

HSPA8

CDH5

REST

IGFBP1

FOXP1

S100A8

IL12RB1

ZFHX3

ABCA1

DVL1

AGT

DRD2

CDK12

ADAM9

DRAIC

MIR494

XDH

ATF1

HSP90B1

LMNA

ATF2

HMMR

STIM1

FOSL1

SLPI

PMAIP1

ADH1C

CDH3

WEE1

PRDM10

MIR495

TG

AGR2

HSPA1A

ACTB

MIR92A1

GPX2

BCL6

ARG1

CPS1

ELF3

FGA

CCL21

BMP7

FOXC2

WNT7A

FLT3

BBC3

CYP24A1

SOD3

CP

BGLAP

DSG3

POT1

PCAT29

MT-CO1

PSCA

PMS1

MIR505

GGT1

FLI1

RECQL

MIR20B

LOC111589215

KLF5

PAEP

MIR128-1

CRKL

CRNDE

SFN

GPER1

IL2RB

GSTM3

LDHA

CXCL9

MUC2

CST3

LGALS3BP

IL16

PSAT1

CCR1

NEDD9

NOX4

ECE1

F13A1

PTPN1

MCAM

MCM4

MAPK10

MTR

RBM10

SPINT2

TGFB3

PAX8

ATF3

IL22

MIR661

PIK3CD

PTPN3

ACP5

KLF2

TAC1

MIR26B

PRF1

IBSP

NCL

BRD4

PSMD4

HSP90AB1

MB

ITGA9

SNCA

ADH1B

ADAMTSL1

ATP7A

NTN1

DROSHA

TBX5

TNFRSF10C

GNRH1

SSTR5

CAMKK2

BCR

EFEMP1

SOX10

MDK

KRT13

IKBKE

FEN1

TGM1

TNFRSF12A

XRCC4

CASP1

AKR1C3

F2RL1

ESM1

LAPTM4B

PIAS3

CEBPB

LIG1

IL17F

GJB2

SLC31A1

SERPINF1

RAD52

TNFSF12

LOC110806263

XBP1

SIRT2

CYP2C19

NPPA

SRF

ALOX15

CYP27B1

FPGS

APP

BNIP3

SRSF1

RETN

CCR4

PTN

SLC6A3

PARK7

DPP4

CORO1C

MIR196B

TERF2

UBE2C

CYB5R3

STUB1

GSR

VTN

TLR7

TNK2

NCOA2

TCF4

KCNQ1

CXADR

CTCF

PELP1

IL10RA

MIR346

MIR502

MIR944

MIR326

COL1A2

TNFRSF11A

GCLC

RPS3A

UHRF1

LYN

SPRY2

LRP6

SIRT3

GHR

PTGER2

PPARA

ATP7B

FOXE1

CBLB

RIPK1

EEF1A1

DLL4

NOTCH4

RPS6

TJP1

IL18R1

TMPRSS4

MIR202

UNG

BCL10

HNRNPA1

PEA15

WNT3A

S100P

CDK5

CIITA

FBLN5

PPARD

IGFBP5

SDC1

CSK

KDM5B

LINC00941

MYB

SPHK1

OPRM1

MIR381

DLST

HELLS

NOP2

PER2

PRDX5

PORCN

CFI

RBX1

CTSG

ATF4

HES1

NUDT1

ALCAM

MIR885

MAP1LC3A

PER1

ESRRA

SPOP

DPP9

TNFRSF10D

AVP

MDM4

KLK6

PRMT1

PSMA4

HNRNPK

TMEFF2

RRM2B

USP9X

HSPG2

IL2RG

ALDH1A2

COPS5

CX3CR1

ACTG1

DVL3

SELENBP1

KRT17

MIR124-2

REV3L

PTP4A3

SLC2A3

FZD4

TSG11

IFNB1

ARRB1

CHRNA1

SPHK2

CASR

BAG6

HTR3A

GPR87

RBMS3

INHA

THBS2

ICOS

SOCS6

MIR181A2

EPHX2

ACE2

VTI1A

TACC3

NIPBL

PAK4

CXCR5

NLRP3

MST1

HYAL1

CD63

MIR663A

ROCK2

TLR8

GDNF

TAB1

ACTA2-AS1

RPS27A

MARCKS

ULK1

SCGB3A1

P4HB

SLC3A2

EIF3A

PF4

MIR33B

CDH10

PTPN6

PRKG1

LEPQTL1

MYOG

OSM

FANCF

CALD1

TNFSF13

GLS

HULC

MIR452

CCL4

HNF4A

SIX1

INVS

H4-16

BCL2L2

LIG3

FBP1

IL6ST

CCNA1

NOS1

TPO

HLA-DRA

MIR92A2

PUF60

MIR498

DLX6-AS1

HOXA1

EXO1

ZBTB7A

ADM

LAMA2

RAB25

PLCG2

TGFBR3

PPP2CA

LINC00707

LCK

MAP1LC3B

UBC

ADAMTS13

SATB1

GGH

H3-2

CRK

SFRP2

RYR1

MIR370

SMIM31

RALA

KCNK3

LASP1

MERTK

GALNT3

CELF1

CDK8

SMARCE1

MBD2

KAT5

ABCC4

UBE3A

HOXB5

MIR7-1

ERRFI1

SEMA3A

GAB1

NR3C2

EEF1A2

MIR16-2

MUC6

CD59

TARDBP

USP22

MAPK7

L1CAM

NT5E

TRPV6

MIR675

LAMA1

TBK1

RBL2

LRIG1

SOX18

SLC27A5

IQGAP1

ARSH

BTRC

IRAK1

LUADT1

PLAT

FOLH1

ADAM8

ATAD2

TTF1

EFNA1

VIPR1

IL32

CCL22

CD81

DAB2

CCL20

EFS

ALDH1A3

PIK3C2A

LINC00319

DERL1

MIR501

MMP26

RELB

TDP1

LINC00115

CD2AP

PPBP

STX1A

NOG

BAG3

SLIT2

LATS1

PTPA

RCN1

KLK10

RSF1

CD69

MSI1

PTPRO

HDAC3

BHLHE40

IL12RB2

CD27

ACHE

EHD1

CCN4

FCGR1A

PTK6

LIF

INPPL1

CRABP2

LINC01512

TUBA1B

PGK1

ROR2

UMPS

GIMAP6

MLANA

CUL4A

CUL3

FAM83B

IRF4

PDK1

AQP5

SELPLG

CSF2RA

ACTR3B

TGIF1

CDC45

LAMTOR5

FABP4

MIR376C

TPX2

FER

RPLP0P2

NODAL

HYAL3

CCK

EHMT2

HYKK

NID1

HBB

TRIM21

SETDB1

FOXA2

ATRX

CRYAB

MS4A1

MIR9-2

PPIA

DNAJB4

EGLN2

KPNA2

COL5A1

TRIM33

TNS4

IFNAR2

EGLN3

MIR590

F10

STC1

C5AR1

FLOT1

RHEB

PRDM14

LINC01511

TP53BP1

MUC5B-AS1

BCL3

CA2

TREM1

RPS6KA3

PTMA

TRPC6

TACSTD2

CKS1B

HSPB2

PTGIS

CHIAP2

TFAP2C

RAB27A

NTSR1

MIR367

PRKCZ-AS1

CD68

BCRP3

NRP2

MTA2

RAD18

ANKS1A

PRKD1

EGFL7

DDX3X

PTBP1

MPG

SMO

GAS6

PKD2

NEDD4

SKI

AKT1S1

PBX1

MIR30C2

IL23A

MIR1290

ALKBH3

RBP1

HDAC5

YWHAG

ZNF295-AS1

RUNX1

STAG3L2

LYPD3

CCNB2

DSG2

SKP1

CD99

CXCR6

BPTF

ACKR3

TUBG1

POLB

PTH1R

CXCL13

LOXL1

RAB5A

PGAM1

JUND

CD151

THY1

CRYGC

KDM5A

SPRY4

NFIB

NSD3

RBBP4

IGF2BP2-AS1

ENSG00000233834

SULF2

UNQ6494

APCDD1L-DT

E2F4

TUBA1A

SPA17

GAL

LOC105371114

DRD4

AQP4

ENSG00000232581

MCM6

NR5A2

SQLE

ENSG00000269652

ENSG00000256694

SNCG

LINC01969

SATB2

EI24

SLC9A3R1

MIR608

KLF9

ITGB2

EMSY

DDIT4

ARPC5

CFH

CLOCK

LINC00635

BMS1P20

PLAC9P1

ENSG00000256343

ELOVL6

VAV3

SLC1A5

FOXP4

NOX1

ANO1

CAVIN1

C4BPA

ALDH7A1

LINC01194

FOXF2

PIGR

RTN4

MIR509-1

MTSS1

TNFAIP8

TPBG

CTHRC1

GPNMB

BIN1

C4B

MIR510

FAM215A

TRPV3

HGS

PKD1

TYK2

PTPN22

CSTA

CUL4B

SIN3A

SGO1

SERBP1

FAP

NORAD

SLC7A11

KLRK1

FABP3

GRAMD4

ST8SIA2

MBD4

GSTA1

MAD2L1

ASAH1

TBX2

SUZ12

SET

CBX3

LIN28B

SLC16A1

TMEM97

TBXAS1

GALC

KMT5A

TIMP4

HSF1

EPHB3

DCLK1

CRABP1

RHO

MIR1-1

IL1RAPL2

YWHAZ

EIF5A

FBN2

POR

MTHFD1

PLCE1

IL37

NR4A3

PHLPP2

IFNGR2

MED12

HSPA9

TRIM58

CYGB

CXCL6

PTGER1

PRAME

SRCIN1

SOX7

PSMC4

CD164

ALOX15B

APOBEC3B

BCL2A1

TBXT

TIE1

HOXA11

LAMP1

BRD7

SNW1

MIR194-1

ING4

PPP2R1A

MED19

MIR1-2

CAV2

IGFBP6

EYA2

IGFBP4

LBR

ROMO1

ID2

MIR136

GH1

FOXP2

FGF4

ADAM28

MAP3K14

GOLPH3

FZD9

EEF2

LILRB2

USP14

MTRR

EGLN1

C8orf34

CLDN18

MIR379

UIMC1

DSC2

HMGCR

FUT3

TMSB4X

H3C1

GART

DSC3

UCP2

GHRH

SLCO1B1

CBS

ID3

BLZF1

MIEN1

POLI

NEDD4L

BMP1

HLTF

ARID2

TFE3

ZEB1-AS1

FLOT2

SELL

CARD10

AQP1

NR1I2

LAG3

COL10A1

PDGFC

CD200

PROX1

SCGB3A2

EGR2

IGF2BP1

KIR3DL1

NAGLU

DDX5

SHOX2

LGALS9

CSTB

ALKBH1

DHRS9

MIR615

SMAD1

BTG2

GLO1

TP53AIP1

MIR133A2

HHIP

USP8

SLC16A4

ITK

CDC20

SALL4

CAMK2D

LRG1

RPS6KB2

IFNAR1

TBXA2R

PRDM2

SEMA4B

EIF3H

BRD3OS

ALOX12

LGR5

NKX2-8

KLF17

SOX17

FRK

PRKAA2

LTF

ARTN

CTNND2

WNT2B

DVL2

CA1

KDM6B

TNNT2

SUMO1

TMSB10

ELP1

NALCN

ARHGEF2

SLC22A2

B3GAT1

FOXO4

ADORA2A

ADAMTS1

CKB

MIR382

NET1

PER3

TERF2IP

HTATIP2

SNHG14

MIR520A

SOX1

AOC3

PTPRU

MICB

MUC7

TEP1

THRA

PAX2

ADAR

FUZ

KHSRP

TRIM25

DYNC2H1

MAGEA10

RND3

PRKCZ

ACP1

EPRS1

THRB

PTX3

MIR588

EIF4G1

IL27

CCNH

LILRB1

NFATC1

F11R

SSX4

CGB5

LAMA3

SIRT7

KAT2A

STYK1

PABPN1

GCLM

ELK1

MIR129-2

ABL2

DUSP3

PRNCR1

LMTK3

TKTL1

DLEU1

CCN6

NNMT

MIR101-2

CYP2C9

MIR211

DEK

RAD17

XXYLT1

LINC01600

XAF1

C5

ABCC5

PYCR1

CDKN2A-DT

HSD3B1

ADIPOR1

MIR365A

IL15RA

ALDH1L1

TFF3

TP53I3

DDB1

AGO2

FGF3

FXYD5

SENP1

ADRB1

TOP2B

NDUFA13

DEFB4A

PTPN12

TNKS

SCN9A

TFAP2B

SMARCC2

IGKC

NBPF3

BTG3

ERN1

IVL

ATG3

TIAM1

LRP1

CDK7

LDHB

PTENP1

TIGAR

SPINT1

TACR1

GSTM2

CTAG1A

PPP1CA

NEK2

HRH4

RIMS2

MAP3K3

PIAS1

NR2C2

YES1

ROR1

PIWIL1

MTA3

YWHAQ

EIF6

TNFAIP3

HOXB2

KPNB1

MIR411

GLDC

ASS1

ST7

SAMD9

ABCC11

IFITM1

KLK8

PBRM1

ADAM10

DSCAM

S100A11

ASPH

ZAP70

ANG

PSMD10

PPARGC1A

PHLPP1

TXNIP

PINK1

PRODH

SMYD2

SNX1

MIR153-2

PYCARD

FSTL1

CD247

OAT

CTDSPL

MAP4K3

TUBB1

STK39

UGT1A9

HMGN1

PINX1

PTP4A1

LORICRIN

MIR410

PITX1

PCDH10

CXCL16

RGS5

HK1

RPL22

PAWR

LRRC3B

MIR584

CD83

XPO5

GSK3A

PAK6

ACLY

TFEB

CFB

NSD2

ZBTB33

FALEC

BRINP1

NLK

GJB1

HOXB7

MAPRE1

F9

CCL27

SRGAP1

PBK

MIR1246

KCNA5

MIR363

MCRS1

BCL11A

ITPR1

CHL1

TXNRD1

IRF2

MIR19B1

CDT1

FDPS

EIF4E2

NR1H3

BOK

MIR138-2

RNF146

GPC5

PARD6A

CUL2

USP28

CCT7

MCM3

MIR506

GAPLINC

MIR153-1

PAFAH1B1

GHSR

GLUL

DLX4

PSPH

ARVCF

HLA-E

CMKLR1

MIR99B

CYP2C8

MRTFA

PKP4

CNR2

CHAT

MT2A

ENAH

BMX

LZTS1

BRF2

ABCC10

AKR1C2

IRAK2

LIN28A

DCTN1

HSD11B2

FZD8

MSH5

CASP4

GSTM4

CHD5

PRSS3

NAPG

SLC25A1

RAN

GRK5

RYR2

FTH1

DCT

SIK2

NR4A1

OLFM4

CPA4

TNFAIP8L2

HTRA1

MIR299

URGCP

GSTO1

CDH8

ELF1

HDAC7

MIER2

RBBP8

FZD1

FOSL2

KAT8

SIX3

CACNA1G

GOLM1

CGB3

TES

CHRNA4

TFPI

ZFX

TRIM29

PSMA1

LOC106721785

NMU

CLDN2

FAM107A

CCL26

CACNA1B

KHDRBS1

FUT6

TRIM16

CRMP1

PTPRH

SLCO2A1

PRSS3P2

TFDP1

KL

TCF21

PADI4

CDR1-AS

S1PR1

ATG5

PLEK

NRXN3

PRDX6

LAMP2

CST6

ACKR1

VCL

SFRP5

FAAH

LINC00339

RIOK2

MIR217

VSIR

TGIF2

EPHA1

SLC5A2

CA4

HNRNPU

APCDD1

GSS

MIR760

PLCL1

SAXO1

RICTOR

SREBF1

PAG1

APOM

RPS3

CD47

ORAI1

CCR9

FUT8

FGF18

RAD23B

HMGB3

MED1

DDC

HSPA1B

ATP5F1A

RAC2

CTBP1

CHN1

MIR613

FST

FURIN

ANXA3

ITGBL1

YEATS4

POU2F1

KIR2DL4

LBP

FOXD3

AKAP4

APLN

PPP2R2A

ST6GAL1

PIWIL2

UGT1A7

USF2

USP25

RBM3

RPTOR

RIN1

SSRP1

SUV39H1

RPA1

ERAP1

MIR1179

SOAT1

KLK11

SPARCL1

C19orf48

UBE2D1

PAF1

MIR337

POLR2A

PROC

MARCKSL1

DEFB1

LBX1

IL1RL1

MGAT5

MIR433

SLC35D2

MAGEA9

CABLES1

PCDH7

RING1

CTBP2

PDIA3

DNM2

PTGES3

EIF2S1

IGF2BP2

LARP1

NR2E3

CYP11A1

SMARCC1

MIR135A2

CARD16

TAGLN

IL20

ESRP1

SYCP3

BATF2

ANLN

DDX17

KDM4A

TOPORS

METAP2

MINDY3

THSD7B

HADHA

POLL

DLL1

RORC

ARRB2

ADCYAP1

SCT

CALM1

TRIM66

GATA5

CDH11

RAP1GDS1

SH2B1

MYBL2

CASS4

HBP1

LINC00290

UGT8

GPI

FAF1

MEF2D

CD38

CDH9

PLAG1

PLAC1

VGF

MSN

MIR208A

KISS1R

FABP5

SIX2

ING2

PPP1R13B

USP13

CDK14

RGMB

HSPA14

PDSS2

FGF19

TAPBP

PRKCG

DDIAS

TMSB15A

MIR500A

RGS17

CHRM2

LEF1-AS1

PTPRK

RALB

DNM1L

CD3EAP

STMN3

FRS2

CCL8

MAP2K5

DAXX

FPR1

TFAM

CLDN6

IRF8

MIR641

RARRES2

LTK

MIR758

DUXAP9

TTK

MRPL58

MIR92B

PDLIM5

CTNNBIP1

FBXW11

FOLR2

HIF3A

AIMP1

TMPO

TNFSF9

TRIM37

MIR329-1

CD163

HSPA2

IGHG1

ERGIC3

ACTN1

MIR629

NNT-AS1

SSTR1

MIR660

ICAM3

USP17L9P

DLG5

GIPC1

NPHP4

CSAG2

SKIV2L

MIR383

GSTA2

PTPRA

ARNT

CRH

IL5RA

LPCAT1

SPRY1

PSMA6

SLC16A7

NHEJ1

HTRA3

CX3CL1

JPT2

PTAFR

MIR384

CPNE1

SMAD9

CAP1

CSAG3

KLF8

ADAM19

ANGPTL2

PTPRT

GPR55

ELAVL2

MMS19

NUDC

MATR3

HAND2-AS1

XAB2

TNKS2

MMP19

SELENOP

SIK3

FUT7

MBD1

RPN2

CAPNS1

SERPINB9

RDH10

MIR1258

CMTM7

CBR1

SEC62

CCL25

EFNB3

RPL3

MIR577

CNPY2

AZIN1

HIF1AN

JMJD6

ARF6

RPA2

NTHL1

FUBP1

SPOCK1

BUD23

BIK

SPAG6

CARD8

PRR11

PSG1

EFNB2

NME2

RPS6KA1

VAV2

ATP2A2

BAMBI

MSI2

CNOT3

PHF20

SLC40A1

LAT

SLC7A2

RPRD1B

TOLLIP

AICDA

H3-4

RABL3

CTCFL

NUMB

SLC39A4

AMD1

ARG2

GML

PLAAT3

GNA15

MIR488

NNAT

CHRNB1

MFN1

DYRK2

KIF11

GSTT2

PDCD6IP

TUBA4A

SLC29A3

SELENOF

NR1H4

PTGR1

GRIN2B

CCNG2

MIR325

GORASP1

IL25

ILF3

LOC111674463

CHKB

CAPN2

SLC28A1

FRAT1

H4C6

U2AF2

ANP32A

PRPF19

MIR493

FES

TMEM14A

DRG2

SRPK1

USF1

LECT2

MIR504

IL10RB

AHNAK

HEY2

VCAN

MYO6

POGLUT1

LSM1

G3BP1

TUBB2A

RFC1

AQP2

VASH1

SNIP1

EIF4A2

CCT6A

AMPH

ZFR

CBLL1

PRMT5

AGK

ACACA

CAVIN2

CYB561D2

MMD

FOXD1

UTP14A

RCC2

HUWE1

RASSF8

PBX2

RPS6KA6

TRADD

HEPACAM

HNRNPF

LMO4

CIAPIN1

NR4A2

E2F8

CCT5

PRDX2

PPIB

SEMA5A

CREG1

CAPG

PTOV1

DPPA4

SIPA1

KLK13

ANKRD1

STOM

JAM3

CNTF

MMP15

JAG2

INPP5D

MAP3K2

MIR652

JUNB

KIR2DL1

LINC-PINT

PLD2

MIR302A

EPB41

RAPGEF1

KIF23

SSX3

GAP43

NFATC4

TAGLN2

ADGRF1

SKAP2

TARBP2

CCL19

METTL3

TRIM59

EPB41L1

CXCL14

RSL1D1

TRIB1

DLGAP5

FBXL19-AS1

MIR484

HBG2

BTBD7

MT1G

RAB11A

EIF4A3

EIF3B

ILF2

HES5

KIFC1

LETM1

RAD9A

WASF3

SLC25A5

ADAM23

DCD

INSM1

FAIM2

LTA4H

EEF1G

APIP

KCNH5

EIF3D

DDX58

KIR2DS4

DDX1

GFPT2

MAL

SEMA4D

PHC3

RHOD

CREBL2

CTSZ

CPEB4

NREP

CLTC

SCUBE2

DUT

GCNT3

RAB27B

NFAT5

ATG16L1

MZB1

BVES

BIRC6

GIT1

TADA3

PYGB

MAP2K3

HPX

HK3

SH3GL2

CHCHD2

E2F7

H2AZ1

TIMD4

WDR26

CABYR

SLC35F2

MT3

MEOX1

E2F6

UBE3C

HLA-S

MAP3K5

PHF8

LRIG2

TRIM47

MORC2

MIR520B

PDE3A

PSMD9

RFPL3

TDGF1P3

NOLC1

THOC1

MAPK12

SLC38A3

TRIM44

PAM

HIP1

LMO1

NOL11

ARL6IP5

RGS4

KCNMA1

SIX4

HHAT

PTPRF

PFN2

PDP1

MAGED4B

ARNT2

DIXDC1

AZU1

BMPR1B

MIB1

MAOA

ARHGAP1

IRF7

SIRT5

PHRF1

PIK3C2B

HCK

DUXAP10

CTSH

MIR515-1

TRAP1

POLQ

RIDA

TNFRSF14

NEU3

ASAP3

RPL35A

TENT5A

CDK9

RAP1B

HMGN5

MCPH1

NDC1

BDKRB2

BLK

SLC9A1

RASGRF2

CST1

AIF1

SLC22A1

CRTC1

RAP2B

VAV1

ITGB5

RAPGEF3

UBQLN1

ST8SIA4

WFS1

VMP1

RAB14

CAVIN3

SAFB

POLA2

CCNY

TIMM50

EP400

KIF15

MAGEB2

PDIA6

MYNN

FOXK2

MIR448

TRIM31

BRK1

UGDH

UACA

BTC

SOSTDC1

SCAP

EMP3

SPAG5

KLK5

PKNOX1

SKA1

SPN

ARHGEF5

ARMC8

USP17L2

PRKAB1

TMBIM6

ATG10

TK2

TMOD3

SEL1L

CDK16

MKNK2

SIX6

PGAM5

DYRK1A

DDT

MED23

EGR3

YEATS2

MIR5100

DHX9

MIR874

PDCD10

MARVELD1

TXNRD2

VPS9D1-AS1

POLDIP3

S100A13

OIP5-AS1

DUSP23

HEYL

NRF1

DUXAP8

GABRA3

QKI

MBD3

GDF10

TSPO

ITGB6

PRPF40A

ALX1

HRH2

TUBG2

EIF2S2

DBI

BCAP31

CD3G

BCORL1

RPL34

PTP4A2

RBL1

HOXC9

APBB1

NEUROD1

SUB1

LINC01088

GINS3

KCNJ3

TXNDC5

ORAI3

SHQ1

CMPK1

IARS2

CST7

CSN1S1

STARD13

SPON2

AMH

MIR575

RGCC

LHX6

MYL9

GPBAR1

MRM2

STEAP3

ALYREF

MLXIPL

BRD2

MIR1204

DCBLD1

MIR302B

MIR365B

MDH1

NKD1

CHAF1B

RCC2-AS1

SRI

TNXB

PRSS50

IL31

WSPAR

MIR300

ISG20

MARK2

ME1

MFN2

CA12

TCN1

MIR3662

DMAP1

USP15

DPPA2

H3-3A

SEPSECS

CCN3

LINC00346

MT1F

PCBP1

SRGN

CCNDBP1

LDOC1

FERMT1

IATPR

MIR635

RASSF3

PLOD2

SEMA4C

MIR124-2HG

DYRK1B

EPN3

NT5C2

GSPT1

GAB2

RPL26L1

KIR2DL3

MIR1271

NBR1

LRP12

TET1

INO80

CENPU

ITGB8

MIR544A

KLC2

TINAGL1

PTGIR

MIR592

CRTC2

CCDC8

MIR487B

MIR194-2

LOC106728418

DAG1

TNFRSF25

SCD

NR2F6

ARHGEF39

AFAP1L1

CSRP3

FBXO7

DLL3

THOP1

IFRD1

SEPHS2

TSPAN32

MYO10

MIR3666

RBFOX1

KIR2DL2

FOXR2

USP49

TNFAIP1

MOS

DENND2D

ADAMTS5

UGCG

L1TD1

EMX2

TRIM14

NEFL

CHMP3

KIAA1522

AIM2

LINC00668

LSINCT5

NPBWR1

ADORA2B

SERPIND1

MIR4293

HORMAD2

BLID

FSCN2

FOSB

ATOX1

NTF3

HNRNPC

REPS2

MIR664A

PRR13

POU2F2

TRHR

ECSCR

NCKAP1

IKZF3

NFE2L1

GLRX

MIR1285-1

C10orf90

ALDH1B1

RUVBL2

ASPM

XCR1

MIR323A

DOK4

DBH

VSNL1

RTCB

RAB11FIP2

ATIC

FERMT2

MAGED4

TM4SF1

IFRD2

MIR1297

ZNF326

CCDC34

MINCR

VAMP2

SPC25

NAIF1

NUAK1

A1BG

SIX5

MIR490

SMOX

FBXW5

EVA1A

LOC108281177

COMETT

SIVA1

MIR302D

CCDC85B

OLIG1

CHN2

NIPSNAP2

DUSP19

DDX51

NOCT

SNAP47

CLSPN

FSIP1

TNFSF15

KLK14

CDH12

NCR3LG1

UAP1

MIR1275

NME4

MLF1

GRIA3

GCAT

RCHY1

UBE2L3

MIR802

DEPDC1B

WDR45

YKT6

GABRB2

CDX1

RASSF2

SOX8

ARMCX3

ATXN3

TRRAP

CLPTM1

TMPRSS11D

ABCG4

PSMA3

EIF3J

MAP4K2

RASGRF1

EYA4

HDAC8

PRAG1

ELMO3

FMNL1

ATP8A1

CEMIP

PCDH20

CYRIB

TRA2B

MSANTD3

MIR329-2

GSDMD

PYY

GPX5

ABCG1

POLDIP2

TIAM2

CSNK1A1

PRKG2

ZNF677

CYP4Z1

RDM1

COX17

HOXD8

MIR3127

ZBTB1

TFIP11

HOXC11

COL23A1

FBXL3

GIPR

PJA2

VWCE

SINHCAF

PVR

NEBL

MIR616

LOC107882129

TNFRSF18

PTGFR

DDX28

MIR449B

CCDC106

TOPBP1

MED10

KRT34

CD160

COMMD9

GSTA4

TEAD2

TPGS2

AKAP12

SRMS

LIMD2

PROZ

GATA2-AS1

RANBP2

CSNK2A2

PDZRN3

TPPP3

TEX10

USP40

FKBP1A

CDK5R1

ANGPTL4

NUPR1

FRS3

TNFAIP8L3

TAB3

NUDT6

RNF135

MIR589

PLCH1

MPHOSPH8

SRSF2

GAS1

IL1F10

NCOA5

PIK3C3

KIF4A

DLX6

STAT2

RPA3

TRIM13

GMEB1

MS4A2

DPYSL2

MIR520F

LYPLA1

PSMA2

SUSD6

ARHGAP27P1

INHBC

PCDHGB6

DOCK3

CCNJ

NRSN2

KIF3A

HEXIM2

MIR576

KLK1

MIR520E

PRM1

BARX2

CCN5

ECE2

MIR939

OTUB2

CLK1

H2AW

ZNF746

B3GALNT1

CPB2

MIR935

CNTN1

RAPGEF5

KIR2DS2

LINC00887

COL6A1

CMTM1

CS

OTUD7B

RHOV

CDC37

MIR770

CNKSR3

LINC00970

ANXA4

PSMD1

DOCK1

MIR1244-1

NR2C2AP

SLC11A2

SMG1

MT1B

PLCB4

NAT8L

PTPRD

MIR598

PEG10

KLF7

TSPAN14

IAPP

FOXQ1

PARD3

NDRG3

ANKRD22

EPS8

MIR526B

CFAP45

PKP2

PKP1

ITGAE

FGF13

LINC00963

SHMT1

MIR7-3

ATG2B

SH3KBP1

SMAD5

MIR3619

PHACTR3

PSMD3

TMED8

SLC5A10

MIR1269A

TRAT1

KLK7

UPK3A

ADGRA2

PGRMC1

MIR4735

SLC47A1

TRPV5

RTL10

MIR4319

SNORD138

PLB1

LRRC34

MIR5195

NMRAL2P

TSPYL2

SYNM

MIR585

ARHGEF19

CSNK2A3

EIF4B

ABCB4

TRIM16L

MORF4L1

TJP2

MIR4295

MIR3120

HOXB8

PKIB

SIAH2

ROBO4

TMEM106B

PPP1R15B

GHRHR

COL6A3

MKRN2

FGR

MIR4443

LHX2

LOC106014249

IL22RA1

HNRNPA3P1

SLC2A13

BCRP1

PCNT

BABAM2-AS1

TAF11

FKBP3

APOC3

MIR1260A

SLC46A1

OGFRP1

MIR515-2

OR2J3

SKIL

KCTD20

TTTY15

ITPRIP

MIR194-2HG

GASAL1

MIR541

MIR4317

RNU7-31P

GAS7

BCHE

PROK2

ACY1

TCP1

LIMD1

PTK7

MIR4782

SERPINB4

STAG2

DNAJA1

SERPINA10

RANBP9

LZTS3

ZNF211

ANK1

DPYSL3

MAML2

TNK2-AS1

SEZ6L

MIR4299

PDCD6

CD207

DISP1

RUVBL1

ERP29

MIR103A2

TBILA

MIR550A3

RAB35

ZBED9

PLAGL1

MIR3163

MIR1253

TUBB6

LOC108281116

ZNF367

HUS1

MIR889

MAGEA5

LINC00221

CYP3A51P

CD109

POLG

TRPC1

MIR181B2

MIR1197

MAGED1

MIR548L

ITIH5

CPQ

MIR1183

DACH1

MIR1238

DACT1

USP10

MIR7-2

LOC100506431

PPP1CB

KIF14

FLII

RBBP7

CD3E

ATG13

PRUNE1

MIR6754

ADIPOR2

CEBPD

CARM1

UHRF2

CYB5A

SLC25A11

LMO7

MIR3940

ENSG00000233340

AMZ2

CKAP4

GINS4

TRIB3

CALM3

RAC3

FLACC1

CRHR1

MAFG

SLCO1B3

KRT3

THEM4

RBM39

HOXA3

GKN2

NUDT21

ULK4

CIZ1

PPIL3

AKIP1

G0S2

MFSD2A

SERINC2

RNU2-1

ENSG00000282218

SMYD3

KIF2C

KDM2A

CACNA2D1

KDM3A

MIR1976

DOK1

ZBED3

SNORD71

EMP1

SCTR

DMP1

ARHGEF7

FAM126B

OCLN

HM13

ATG16L2

KMT2C

BTBD11

GLUD1

MIR362

SEC62-AS1

EIF4A1

POLD4

HMGB2

OGFR

RPL22P1

NR2F1

GALNT14

CCT2

BAIAP2

GDF5

ADAMTS8

S100A10

CCT3

SCRIB

PTGDS

PPAT

MIR301A

PNPLA2

CASP5

RUNX1T1

PPP1R15A

ZYG11A

ULK2

CDK3

SORT1

RRAD

EDIL3

DUSP13

TGM5

LZTFL1

MASP1

UBAP2L

TENM3-AS1

USP1

ENSG00000226181

CHRNA9

ORM1

MIR638

PCK2

DAPK3

MEST

MSRA

PIAS2

SH2D3A

UVRAG

TUBB4B

MGST1

FMOD

FXR1

PFKFB4

RDX

HTRA2

CAPN1

MANF

SFRP4

BCL9

BHMT

MEIS1

SSPN

KIR3DL2

LINC02500

ENSG00000225356

ALDH3A1

YWHAH

ELK3

LZTS2

PRMT6

TMPO-AS1

TRIM65

SYMPK

MIR744

HNRNPR

PAX9

FCHSD2

TRDMT1

LOC441750

ENSG00000279729

ACYP1

STK33

CPSF4

INTS14

CKAP5

ST3GAL4

ABI3BP

IFIT3

CDON

CDCA5

UHRF1BP1

MAPKAP1

LIMCH1

NECTIN4

VSIG1

NKIRAS1

PKP3

ELOB

CIC

TRIB2

ANAPC1

PSIP1

TRIP10

RACGAP1

COPS3

FGFR1OP

PLPP3

WSCD1

SMURF2

MIR489

ITPR3

KRT4

DCHS1

PEAK1

SLC5A1

SCYL2

GUCY2F

MADD

FRMD3

FOXP4-AS1

AQP9

SART1

TUBB2B

NUCKS1

CPM

CCT8

NFYC

KIF16B

TOB1

TAX1BP1

MIR422A

RPL23

NFIA

ATP1A1

ABT1

COPB2

DSPP

NDUFS1

CCKAR

SRXN1

HORMAD1

DHODH

POLD3

UBE2L6

RGS3

PCBP4

KDM8

DAZL

MAP4

PFDN1

ATP6AP1

TRARG1

MIR421

LINC00641

GABBR1

MNS16A

CHD1L

CCT4

POU3F3

ADAM15

ATG14

PPP1CC

USP2

BTG1

POLR2L

KIF18A

EGFL6

TSPAN4

SYNE3

KLKB1

PYGO2

CACUL1

TMEM88

SERPINA5

DKK2

INS-IGF2

PATZ1

TNS3

RAMP2

TDP2

NCK1

WASL

PTPRN

FUT1

MAPK8IP2

NUF2

LHX3

MIR662

MIR940

HAS3

TWF1

FOXJ2

TBC1D7

MLN

GRB10

PROX2

TNFAIP2

NID2

LINC00525

NIN

MIR26A2

MAP2K6

GABARAP

FEZF1

MZF1

OSR1

HNRNPM

FLVCR1

SPON1

RAB37

SLC12A2

MAGI2

CALM2

CCP110

SEPTIN4

MYEOV

LOC111255642

CYTH1

SOX6

SLCO6A1

MAPK6

TRIT1

WTAP

SLC17A8

SSBP3

AGPS

LOC108254682

DNM1

TMEM205

ING5

BAZ1A

KRT75

MAP4K4

NUCB2

INMT

LOC111255645

PTPRB

ESPL1

MAN2A1

GABPB1-IT1

SETD7

CUL5

HS3ST2

PPP6R3

PPP2CB

RASSF10

ATP2A3

DCUN1D1

CUL7

PPARGC1B

WDCP

CAND1

GAGE1

SIRT4

ELOA

MGA

ADAMTS7

CTNNAL1

ATXN7

LINC02602

GATD3A

GAS2L3

CALCOCO2

SBNO1

KHDC4

SERPINB13

RBM4

INTS3

RTN1

TMEM115

MASTL

EIF4G2

HES3

VSIG4

C1orf87

OLIG2

ZBTB7C

GABBR2

ESYT1

DBNL

SH2D3C

SLC6A6

CLTB

FOXN3

COPS6

MAGEC1

PARP4

MRPL28

NUP205

TMEM26

HNRNPH1

TSPAN8

CHMP4C

SIGMAR1

VLDLR

PARD6B

POLR2H

GLRX2

SGTA

RPS6KL1

MTHFS

NPY4R

RUSC2

DNAJA3

HERC5

BNC1

KIN

CEP152

PAPSS1

GIT2

DCSTAMP

SHCBP1

GPS1

OIP5

SNX9

BMP5

DDX39A

SORD

DIO3

NFASC

STK3

PAK5

ZC2HC1C

CENPH

CFAP77

ULBP1

ARHGAP5

PSMC1

HHLA2

NIT1

SLC38A4

ZAR1

TMEM98

MMP24

SNRPC

OSCAR

TESC

DKK4

FOLR3

PCDH15

TDG

TSN

SPNS2

HIPK3

XRN2

PPP1R3A

PITPNM3

AOC1

ATG4A

CFHR1

MEOX2

CEACAM8

RPL19

CMSS1

IL17C

NDC80

ULBP2

NDUFS2

CALHM6

BCL2L15

CPD

CALB1

DDX23

COPS8

CD37

TSPAN1

PPP1R9B

MORF4L2

KPNA4

PHACTR2

EML2

REPIN1

SEMG1

ITSN2

DFFA

CBLC

RPL4

PRDM15

NUMBL

COPS2

PHF14

C7

FAM83F

NCK2

PCNX1

MIR655

ILRUN

MAP3K9

PIDD1

ANGPT4

ATP5PD

SUMO2

TSPAN15

MOAP1

UCK2

PIAS4

H1-1

SIGLEC9

RRBP1

STK24

CAMK1

MCTS1

LINC01354

RGN

TTLL7

LNX1

TRIM22

MIR573

MIR769

STRADB

LIPH

RPL14

APBB2

TSPYL5

VWDE

NCAPG2

RNF7

PCNP

CRIM1

SPIB

CIR1

CABCOCO1

ZC3HC1

HS3ST3B1

MPZL2

DEFA1B

RNF111

IMP3

CSTF2

SNORA80E

UBE2S

EML1

SNHG3

OR10G2

PSRC1

FUT5

TMEM158

MYLK2

MIR548B

MAP4K1

BAGE

COPS7B

GULP1

SCRN1

PLK2

NDUFS8

COPS7A

DLX5

GRAP2

RTKN

DCAF1

MAP1LC3C

FAM83A

DLG1

PLEKHO1

ZWINT

FAM43A

SHC4

COPS4

RBM14

CDKN2D

MPZL3

IRF9

FIGN

MIR3137

CLTCL1

VPS4B

FBXO46

CADM3

SHISA3

THEG

NANOS3

DHX33

CADM4

CD84

ASB7

SYF2

STRAP

ANKRD7

ZNF254

PTRH2

PRMT3

VDAC2

CAMLG

STAP2

INO80D

SUPT6H

SYT1

ADGRF3

ZDHHC5

SLC5A4

CHODL

SLC35F1

RASSF4

SLA

PLAC8

ENSG00000285269

LYNX1

TMEM196

MIR875

ATP1A2

ASCL2

SH3GL3

STMN2

ME2

FEN1P1

UFL1

PDE2A

PDK2

UBE2M

LCP2

PPP2R2D

TRIM49

MIR1287

GUK1

EEA1

RHPN1-AS1

PTPN18

LINC01503

RCCD1

ZMYND11

LAPTM5

FBXW4

PARP3

ATF5

PADI3

PLAAT1

RUBCN

ENOSF1

GPRC5B

VDAC3

KLRB1

UCN3

OTUB1

IPO8

PAGE1

MIR449C

EML3

MIR1182

RNF25

USP33

CCNF

MIR761

DIP2A

NCAN

SLC25A10

ASAP2

CPNE3

SESN3

MIR570

OTUD6B

EFHD2

WNK2

LINC02470

MIR1236

PDIA2

BCAN

LINC00328

CFAP58

MIR1908

SMARCD1

COL8A1

MACIR

SUPT16H

SENP2

LOC108663987

MAGEA10-MAGEA5

SERPINB7

TADA2A

NFS1

GPR65

SLA2

KSR1

GPR171

CENPJ

ZSCAN31

ATP1A3

NHLH1

SLCO1A2

FYCO1

NEK4

TMEM17

FBXO25

LINC02600

REPS1

CEP72

BCL2L10

IPO5

PHF5A

KIAA1217

ZSCAN4

SBSN

PTCRA

RABEP2

RAD1

KPNA3

IWS1

GMCL1

TAF9B

LINC00630

RALGPS2

EPS15

AMMECR1

UBE2F

TAF10

LINC01628

CARD8-AS1

NHLH2

GLRA1

VCX

CD300LG

PPP1R3C

TRIM71

PPP1R3D

WDHD1

RAB11FIP3

GAGE12I

KIR2DS5

KCNA4

MPP3

ATP5MG

RGS20

DYNLL2

PACC1

BAGE3

FBXO17

BEND4

BAGE4

BAGE5

MRPL19

VTRNA1-3

VTRNA1-2

ST7L

MIR4478

GPR135

CDHR5

GTF2F1

VTRNA1-1

ECHDC2

TRIM36

HPCAL1

VTRNA3-1P

LOC109433677

TMPRSS11B

SEPTIN10

RNF138

MIR1298

CA14

CDK5R2

THORLNC

LANCL1

PLEKHB1

ART3

GAGE2C

GAGE5

USP27X

GAGE2A

GAGE6

KIR2DS1

GAGE4

KIR3DL3

SREBF2-AS1

PALD1

PGAP1

KIR2DL5A

KIR2DS3

LINC02598

TALAM1

GAGE2B

KIR3DP1

COG8

TMED6

HPCAL4

ALKAL2

KIR3DS1

KIR2DP1

SLC22A14

SLC22A13

ALKAL1

OTX2-AS1

A2M

TOP1MT

MAP2

TUBD1

TUBE1

5-LOX

CDK

CHK1

D2R

Erbb-3

Erbb-4

FLT-3

FNTB

Ftase

GNEF

HER2

HGF/Met pathway

IL2R

ITGA5/B1

LDL-R

Malaria DHOdehase

MAP3K

MEK1

MEK2

MOP

MPIP2

Nrf2

NY-ESO-1

PD-1

PDGFR

PK

PREP

PRKCA mRNA

PTK

TLR

TRAIL-R1

TrkA

TrkB

TrkC

VEGFR
