# Supplementary material for: Anticancer Action of Xiaoxianxiong Tang in Non-Small Cell Lung Cancer by Pharmacological Analysis and Experimental Validation
Source: Evid Based Complement Alternat Med. 2021 Dec 13;2021:9930082. doi: 10.1155/2021/9930082 (PMC8687818; doi:10.1155/2021/9930082)
Supplement: Supplementary Materials — All primer sets in the RT-qPCR array are shown in the Table 1. Table 2 indicates the targets in XXXT. Table 3 indicates the targets related to NSCLC. Table 4 indicates common targets of NSCLC and XXXT. Table 5 indicates the result of the RT-qPCR array in H460 cells. Table 5 indicates the result of the RT-qPCR array in A549 cells. [file 9930082.f1.zip › 9930082.f1/Supplementary table 5-The result of RT-qPCR Array in H460 cells.pdf]

Raw data

Sample Name	Target Name	C_T	Internal Reference	Fold
CONTROL1	BCL2	32.64707184	19.99020386	0.000154847
CONTROL2	BCL2	34.92647552	20.1919899	3.66841E-05
CONTROL3	BCL2	34.66990662	20.51607132	5.48619E-05
XXXT1	BCL2	Undetermined	22.00503922	#VALUE!
XXXT2	BCL2	36.92548752	20.864048	1.46226E-05
XXXT3	BCL2	35.06748581	20.60765839	4.4377E-05
CONTROL1	BIRC5	30.93034554	19.99020386	0.000508966
CONTROL2	BIRC5	31.92046738	20.1919899	0.000294698
CONTROL3	BIRC5	33.9508667	20.51607132	9.03075E-05
XXXT1	BIRC5	Undetermined	22.00503922	#VALUE!
XXXT2	BIRC5	35.27159882	20.864048	4.60145E-05
XXXT3	BIRC5	35.94173813	20.60765839	2.42093E-05
CONTROL1	CCNA2	30.73538017	19.99020386	0.000582612
CONTROL2	CCNA2	31.92052841	20.1919899	0.000294685
CONTROL3	CCNA2	31.97240829	20.51607132	0.000355876
XXXT1	CCNA2	35.93414307	22.00503922	6.41094E-05

XXXT2 CCNA2 34.33424759 20.864048 8.81183E-05

XXXT3 CCNA2 34.65552139 20.60765839 5.90435E-05

CONTROL1 CD40LG Undetermined 19.99020386 #VALUE!

CONTROL2 CD40LG Undetermined 20.1919899 #VALUE!

CONTROL3 CD40LG Undetermined 20.51607132 #VALUE!

XXXT1 CD40LG Undetermined 22.00503922 #VALUE!

XXXT2 CD40LG Undetermined 20.864048 #VALUE!

XXXT3 CD40LG Undetermined 20.60765839 #VALUE!

CONTROL1 CHEK1 31.43787384 19.99020386 0.000358021

CONTROL2 CHEK1 31.68288803 20.1919899 0.000347452

CONTROL3 CHEK1 31.91698265 20.51607132 0.000369814

XXXT1 CHEK1 34.90649414 22.00503922 0.0001307

XXXT2 CHEK1 32.28730392 20.864048 0.000364131

XXXT3 CHEK1 32.34126663 20.60765839 0.000293651

CONTROL1 CYCS 25.50612068 19.99020386 0.021854636

CONTROL2 CYCS 25.57483292 20.1919899 0.023966399

CONTROL3 CYCS 25.90448761 20.51607132 0.023873993

XXXT1 CYCS 26.89996719 22.00503922 0.033610878

XXXT2 CYCS 25.88450432 20.864048 0.030810025

XXXT3 CYCS 25.4900856 20.60765839 0.033903377

CONTROL1	EGLN1	30.87244034	19.99020386	0.00052981
CONTROL2	EGLN1	29.92252541	20.1919899	0.00117711
CONTROL3	EGLN1	30.75870895	20.51607132	0.000825389
XXXT1	EGLN1	32.70209503	22.00503922	0.000602373
XXXT2	EGLN1	31.91850662	20.864048	0.000470193
XXXT3	EGLN1	31.63968849	20.60765839	0.00047756
CONTROL1	FOSL1	28.92082596	19.99020386	0.002049344
CONTROL2	FOSL1	29.34636879	20.1919899	0.00175492
CONTROL3	FOSL1	29.92723083	20.51607132	0.001468787
XXXT1	FOSL1	25.92425346	22.00503922	0.066099618
XXXT2	FOSL1	24.75031853	20.864048	0.067626358
XXXT3	FOSL1	23.97697449	20.60765839	0.096768674
CONTROL1	FOSL2	27.90191078	19.99020386	0.00415278
CONTROL2	FOSL2	27.93655014	20.1919899	0.004662889
CONTROL3	FOSL2	28.30841637	20.51607132	0.004510975
XXXT1	FOSL2	30.93302536	22.00503922	0.002053092
XXXT2	FOSL2	30.37039566	20.864048	0.001375005
XXXT3	FOSL2	29.34939575	20.60765839	0.002336011
CONTROL1	GAPDH	19.99020386	19.99020386	1
CONTROL2	GAPDH	20.1919899	20.1919899	1

CONTROL3	GAPDH	20.51607132	20.51607132	1
XXXT1	GAPDH	22.00503922	22.00503922	1
XXXT2	GAPDH	20.864048	20.864048	1
XXXT3	GAPDH	20.60765839	20.60765839	1
CONTROL1	HIF1A	28.92974472	19.99020386	0.002036714
CONTROL2	HIF1A	29.59176445	20.1919899	0.001480423
CONTROL3	HIF1A	28.92136765	20.51607132	0.002949536
XXXT1	HIF1A	27.87467003	22.00503922	0.017102715
XXXT2	HIF1A	27.79003334	20.864048	0.008223764
XXXT3	HIF1A	27.32315254	20.60765839	0.00951557
CONTROL1	HK2	31.92755318	19.99020386	0.000254976
CONTROL2	HK2	31.60514259	20.1919899	0.00036669
CONTROL3	HK2	31.92447281	20.51607132	0.000367899
XXXT1	HK2	32.62017822	22.00503922	0.000637565
XXXT2	HK2	32.67989349	20.864048	0.000277381
XXXT3	HK2	31.41630745	20.60765839	0.000557535
CONTROL1	IL2	Undetermined	19.99020386	#VALUE!
CONTROL2	IL2	37.10388184	20.1919899	8.10986E-06
CONTROL3	IL2	Undetermined	20.51607132	#VALUE!
XXXT1	IL2	Undetermined	22.00503922	#VALUE!
XXXT2	IL2	Undetermined	20.864048	#VALUE!
XXXT3	IL2	Undetermined	20.60765839	#VALUE!

CONTROL1	MMP3	36.99795914	19.99020386	7.58849E-06
CONTROL2	MMP3	Undetermined	20.1919899	#VALUE!
CONTROL3	MMP3	Undetermined	20.51607132	#VALUE!
XXXT1	MMP3	32.67039108	22.00503922	0.000615757
XXXT2	MMP3	31.93861961	20.864048	0.000463684
XXXT3	MMP3	31.12330246	20.60765839	0.000683087
CONTROL1	NFATC1	32.9472084	19.99020386	0.000125763
CONTROL2	NFATC1	33.93345642	20.1919899	7.3014E-05
CONTROL3	NFATC1	33.52214432	20.51607132	0.000121558
XXXT1	NFATC1	33.9341011	22.00503922	0.000256445
XXXT2	NFATC1	32.74285126	20.864048	0.000265536
XXXT3	NFATC1	31.97142601	20.60765839	0.000379459
CONTROL1	PIK3CG	Undetermined	19.99020386	#VALUE!
CONTROL2	PIK3CG	35.9360199	20.1919899	1.82211E-05
CONTROL3	PIK3CG	Undetermined	20.51607132	#VALUE!
XXXT1	PIK3CG	Undetermined	22.00503922	#VALUE!
XXXT2	PIK3CG	Undetermined	20.864048	#VALUE!
XXXT3	PIK3CG	Undetermined	20.60765839	#VALUE!
CONTROL1	PRKCB	33.75295639	19.99020386	7.19447E-05
CONTROL2	PRKCB	34.918396	20.1919899	3.68901E-05
CONTROL3	PRKCB	34.95489502	20.51607132	4.50278E-05

XXXT1	PRKCB	33.79996109	22.00503922	0.000281433
XXXT2	PRKCB	33.90086746	20.864048	0.000118994
XXXT3	PRKCB	32.62563705	20.60765839	0.000241117
CONTROL1	PTGS2	34.64784622	19.99020386	3.8691E-05
CONTROL2	PTGS2	37.02415848	20.1919899	8.57062E-06
CONTROL3	PTGS2	32.83306885	20.51607132	0.000195981
XXXT1	PTGS2	Undetermined	22.00503922	#VALUE!
XXXT2	PTGS2	Undetermined	20.864048	#VALUE!
XXXT3	PTGS2	35.70672226	20.60765839	2.84924E-05
CONTROL1	RELA	29.52218056	19.99020386	0.001350794
CONTROL2	RELA	29.87374878	20.1919899	0.001217587
CONTROL3	RELA	29.93252754	20.51607132	0.001463404
XXXT1	RELA	30.93292999	22.00503922	0.002053228
XXXT2	RELA	29.48298454	20.864048	0.002543558
XXXT3	RELA	29.25189018	20.60765839	0.002499349
CONTROL1	SERPINE1	28.92078018	19.99020386	0.002049409
CONTROL2	SERPINE1	27.92611122	20.1919899	0.004696751
CONTROL3	SERPINE1	28.36801338	20.51607132	0.004328425

XXXT1 SERPINE1 27.61775208 22.00503922 0.020436432

XXXT2 SERPINE1 26.70927811 20.864048 0.017394438

XXXT3 SERPINE1 25.87135887 20.60765839 0.026029646

CONTROL1 SPP1 25.92579651 19.99020386 0.016338364

CONTROL2 SPP1 25.95459366 20.1919899 0.018419737

CONTROL3 SPP1 24.93078232 20.51607132 0.04688561

XXXT1 SPP1 26.83480835 22.00503922 0.035163704

XXXT2 SPP1 25.80044174 20.864048 0.032658589

XXXT3 SPP1 25.84152222 20.60765839 0.026573576

Relative expression fold

Target Name CONTROL-1 CONTROL-2 CONTROL-3 XXXT-1 XXXT-2 XXXT-3

BCL2 0.000154847 3.66841E-05 5.48619E-05 1.46226E-05 4.4377E-05

BIRC5 0.000508966 0.000294698 9.03075E-05 4.60145E-05 2.42093E-05

CCNA2 0.000582612 0.000294685 0.000355876 6.41094E-05 8.81183E-05 5.90435E-05

CD40LG

CHEK1 0.000358021 0.000347452 0.000369814 0.0001307 0.000364131 0.000293651

CYCS 0.021854636 0.023966399 0.023873993 0.033610878 0.030810025 0.033903377

EGLN1 0.00052981 0.00117711 0.000825389 0.000602373 0.000470193 0.00047756

FOSL1 0.002049344 0.00175492 0.001468787 0.066099618 0.067626358 0.096768674

FOSL2 0.00415278 0.004662889 0.004510975 0.002053092 0.001375005 0.002336011

HIF1A 0.002036714 0.001480423 0.002949536 0.017102715 0.008223764 0.00951557

HK2 0.000254976 0.00036669 0.000367899 0.000637565 0.000277381 0.000557535

IL2 8.10986E-06

MMP3 7.58849E-06 0.000615757 0.000463684 0.000683087

NFATC1 0.000125763 7.3014E-05 0.000121558 0.000256445 0.000265536 0.000379459

PIK3CG 1.82211E-05

PRKCB 7.19447E-05 3.68901E-05 4.50278E-05 0.000281433 0.000118994 0.000241117

PTGS2 3.8691E-05 8.57062E-06 0.000195981 2.84924E-05

RELA 0.001350794 0.001217587 0.001463404 0.002053228 0.002543558 0.002499349

SERPINE1 0.002049409 0.004696751 0.004328425 0.020436432 0.017394438 0.026029646

SPP1 0.016338364 0.018419737 0.04688561 0.035163704 0.032658589 0.026573576

fold change

Target Name	XXXT/CONTROL
BIRC5	0.117828969
CCNA2	0.171323137
PTGS2	0.351406819
BCL2	0.359179903
FOSL2	0.432525066
EGLN1	0.612139317
CHEK1	0.733275761
SPP1	1.156192782
CYCS	1.410778955
HK2	1.488008362
RELA	1.760047653
NFATC1	2.814059628
PRKCB	4.169591067
HIF1A	5.387940434
SERPINE1	5.766402792

FOSL1 43.71182107

MMP3 77.42103819

CD40LG #DIV/0!

IL2 #DIV/0!

PIK3CG #DIV/0!

P value

Target Name	XXXT/CONTROL	Differences Annotation
BCL2	0.358981231	ns
BIRC5	0.191078915	ns
CCNA2	0.018004192	*
CD40LG	#DIV/0!	#DIV/0!
CHEK1	0.240548274	ns
CYCS	0.001366612	**
EGLN1	0.16326778	ns
FOSL1	0.001671713	**
FOSL2	0.001450043	**
HIF1A	0.027913477	*
HK2	0.235635323	ns
IL2	#DIV/0!	#DIV/0!
MMP3	#DIV/0!	#DIV/0!
NFATC1	0.010821767	*
PIK3CG	#DIV/0!	#DIV/0!

PRKCB	0.03128117	*
PTGS2	#DIV/0!	#DIV/0!
RELA	0.00402784	**
SERPINE1	0.002711281	**
SPP1	0.697671924	ns
