# Supplementary material for: Anticancer Action of Xiaoxianxiong Tang in Non-Small Cell Lung Cancer by Pharmacological Analysis and Experimental Validation
Source: Evid Based Complement Alternat Med. 2021 Dec 13;2021:9930082. doi: 10.1155/2021/9930082 (PMC8687818; doi:10.1155/2021/9930082)
Supplement: Supplementary Materials — All primer sets in the RT-qPCR array are shown in the Table 1. Table 2 indicates the targets in XXXT. Table 3 indicates the targets related to NSCLC. Table 4 indicates common targets of NSCLC and XXXT. Table 5 indicates the result of the RT-qPCR array in H460 cells. Table 5 indicates the result of the RT-qPCR array in A549 cells. [file 9930082.f1.zip › 9930082.f1/Supplementary table 6-The result of RT-qPCR Array in A549 cells.pdf]

Raw data

Sample Name	Target Name	C_T	Internal Reference	Fold
CONTROL-1	BCL2	Undetermined	18.94035149	#VALUE!
CONTROL-2	BCL2	32.90028381	18.92997932	6.23045E-05
CONTROL-3	BCL2	33.67346191	18.45721054	2.62695E-05
XXXT-1	BCL2	36.96279526	18.58896065	2.94391E-06
XXXT-2	BCL2	36.53224182	18.36195374	3.38999E-06
XXXT-3	BCL2	34.69328308	17.94250107	9.06801E-06
CONTROL-1	BIRC5	28.7615757	18.94035149	0.001105394
CONTROL-2	BIRC5	27.92752266	18.92997932	0.001956454
CONTROL-3	BIRC5	27.79945183	18.45721054	0.001540654
XXXT-1	BIRC5	31.21418381	18.58896065	0.000158281
XXXT-2	BIRC5	29.93763351	18.36195374	0.000327622
XXXT-3	BIRC5	30.86330795	17.94250107	0.000128958
CONTROL-1	CCNA2	25.92438507	18.94035149	0.007899442
CONTROL-2	CCNA2	25.96118546	18.92997932	0.007645327
CONTROL-3	CCNA2	25.91740036	18.45721054	0.005678833

XXXT-1	CCNA2	28.59173012	18.58896065	0.00097469
XXXT-2	CCNA2	27.96027565	18.36195374	0.001290082
XXXT-3	CCNA2	27.46230507	17.94250107	0.001362239
CONTROL-1	CD40LG	Undetermined	18.94035149	#VALUE!
CONTROL-2	CD40LG	Undetermined	18.92997932	#VALUE!
CONTROL-3	CD40LG	Undetermined	18.45721054	#VALUE!
XXXT-1	CD40LG	Undetermined	18.58896065	#VALUE!
XXXT-2	CD40LG	Undetermined	18.36195374	#VALUE!
XXXT-3	CD40LG	Undetermined	17.94250107	#VALUE!
CONTROL-1	CHEK1	36.94052505	18.94035149	3.81424E-06
CONTROL-2	CHEK1	27.82336617	18.92997932	0.002102925
CONTROL-3	CHEK1	28.91218567	18.45721054	0.000712425
XXXT-1	CHEK1	29.27266884	18.58896065	0.000607972
XXXT-2	CHEK1	29.81833267	18.36195374	0.000355866
XXXT-3	CHEK1	28.50265694	17.94250107	0.000662333
CONTROL-1	CYCS	21.97618484	18.94035149	0.121933516
CONTROL-2	CYCS	21.29973602	18.92997932	0.19347825
CONTROL-3	CYCS	20.90453339	18.45721054	0.183350633

XXXT-1 CYCS 23.35526848 18.58896065 0.03674501

XXXT-2 CYCS 22.48207664 18.36195374 0.057506829

XXXT-3 CYCS 22.93551064 17.94250107 0.031401786

CONTROL-1 EGLN1 26.94239616 18.94035149 0.003900718

CONTROL-2 EGLN1 27.91569328 18.92997932 0.001972562

CONTROL-3 EGLN1 27.78423119 18.45721054 0.001556994

XXXT-1 EGLN1 28.61514282 18.58896065 0.000959

XXXT-2 EGLN1 28.42019653 18.36195374 0.000937923

XXXT-3 EGLN1 27.76280975 17.94250107 0.001106095

CONTROL-1 FOSL1 24.9290905 18.94035149 0.015747438

CONTROL-2 FOSL1 25.93550301 18.92997932 0.007782645

CONTROL-3 FOSL1 25.4110527 18.45721054 0.008066496

XXXT-1 FOSL1 25.959795 18.58896065 0.006041681

XXXT-2 FOSL1 25.94717979 18.36195374 0.005207382

XXXT-3 FOSL1 25.53736687 17.94250107 0.005172703

CONTROL-1 FOSL2 26.94328499 18.94035149 0.003898315

CONTROL-2 FOSL2 26.92956352 18.92997932 0.003907376

CONTROL-3 FOSL2 26.86538124 18.45721054 0.002943665

XXXT-1	FOSL2	27.34586525	18.58896065	0.002311581
XXXT-2	FOSL2	27.35324478	18.36195374	0.001964951
XXXT-3	FOSL2	26.83116341	17.94250107	0.002109823
CONTROL-1	GAPDH	18.94035149	18.94035149	1
CONTROL-2	GAPDH	18.92997932	18.92997932	1
CONTROL-3	GAPDH	18.45721054	18.45721054	1
XXXT-1	GAPDH	18.58896065	18.58896065	1
XXXT-2	GAPDH	18.36195374	18.36195374	1
XXXT-3	GAPDH	17.94250107	17.94250107	1
CONTROL-1	HIF1A	27.56769371	18.94035149	0.002528781
CONTROL-2	HIF1A	26.70961952	18.92997932	0.004550875
CONTROL-3	HIF1A	26.92477798	18.45721054	0.002824933
XXXT-1	HIF1A	26.92595863	18.58896065	0.003092527
XXXT-2	HIF1A	26.2719326	18.36195374	0.004157757
XXXT-3	HIF1A	25.31743622	17.94250107	0.006024532
CONTROL-1	HK2	34.67639542	18.94035149	1.83222E-05
CONTROL-2	HK2	32.91872025	18.92997932	6.15134E-05
CONTROL-3	HK2	33.06332779	18.45721054	4.00978E-05
XXXT-1	HK2	35.21918869	18.58896065	9.85831E-06
XXXT-2	HK2	37.0092392	18.36195374	2.43561E-06

XXXT-3	HK2	33.3614006	17.94250107	2.2827E-05
CONTROL-1	IL2	Undetermined	18.94035149	#VALUE!
CONTROL-2	IL2	Undetermined	18.92997932	#VALUE!
CONTROL-3	IL2	36.95107269	18.45721054	2.7089E-06
XXXT-1	IL2	Undetermined	18.58896065	#VALUE!
XXXT-2	IL2	Undetermined	18.36195374	#VALUE!
XXXT-3	IL2	Undetermined	17.94250107	#VALUE!
CONTROL-1	MMP3	37.10719299	18.94035149	3.3981E-06
CONTROL-2	MMP3	35.91991806	18.92997932	7.68279E-06
CONTROL-3	MMP3	36.62386703	18.45721054	3.39853E-06
XXXT-1	MMP3	30.22333145	18.58896065	0.000314561
XXXT-2	MMP3	29.54805374	18.36195374	0.000429189
XXXT-3	MMP3	30.7139473	17.94250107	0.000143025
CONTROL-1	NFATC1	29.7864418	18.94035149	0.000543252
CONTROL-2	NFATC1	29.93122482	18.92997932	0.00048786
CONTROL-3	NFATC1	29.7252636	18.45721054	0.000405488
XXXT-1	NFATC1	30.94007683	18.58896065	0.000191401
XXXT-2	NFATC1	30.73774719	18.36195374	0.000188155
XXXT-3	NFATC1	30.51773834	17.94250107	0.000163861
CONTROL-1	PIK3CG	Undetermined	18.94035149	#VALUE!
CONTROL-2	PIK3CG	34.92657852	18.92997932	1.52948E-05

CONTROL-3	PIK3CG	Undetermined	18.45721054	#VALUE!
XXXT-1	PIK3CG	32.03825378	18.58896065	8.94045E-05
XXXT-2	PIK3CG	32.60496902	18.36195374	5.15733E-05
XXXT-3	PIK3CG	37.02394867	17.94250107	1.80265E-06
CONTROL-1	PRKCB	37.09270096	18.94035149	3.4324E-06
CONTROL-2	PRKCB	Undetermined	18.92997932	#VALUE!
CONTROL-3	PRKCB	35.90699768	18.45721054	5.58587E-06
XXXT-1	PRKCB	Undetermined	18.58896065	#VALUE!
XXXT-2	PRKCB	32.73422623	18.36195374	4.71536E-05
XXXT-3	PRKCB	Undetermined	17.94250107	#VALUE!
CONTROL-1	PTGS2	36.92338562	18.94035149	3.85982E-06
CONTROL-2	PTGS2	32.95550537	18.92997932	5.99647E-05
CONTROL-3	PTGS2	36.61608124	18.45721054	3.41692E-06
XXXT-1	PTGS2	36.9136734	18.58896065	3.04587E-06
XXXT-2	PTGS2	36.59457397	18.36195374	3.24664E-06
XXXT-3	PTGS2	35.55836105	17.94250107	4.97849E-06
CONTROL-1	RELA	26.62308884	18.94035149	0.004867048
CONTROL-2	RELA	26.92783928	18.92997932	0.003912049

CONTROL-3	RELA	26.42966843	18.45721054	0.00398154
XXXT-1	RELA	24.8014183	18.58896065	0.013485402
XXXT-2	RELA	24.93778801	18.36195374	0.010482784
XXXT-3	RELA	24.552948	17.94250107	0.010234278
CONTROL-1	SERPINE1	26.30967522	18.94035149	0.00604801
CONTROL-2	SERPINE1	26.91644096	18.92997932	0.003943079
CONTROL-3	SERPINE1	26.89323235	18.45721054	0.002887383
XXXT-1	SERPINE1	24.96476173	18.58896065	0.012041834
XXXT-2	SERPINE1	24.92829895	18.36195374	0.01055196
XXXT-3	SERPINE1	24.42243767	17.94250107	0.011203268
CONTROL-1	SPP1	24.95533562	18.94035149	0.015463555
CONTROL-2	SPP1	23.82666969	18.92997932	0.033569844
CONTROL-3	SPP1	23.92808723	18.45721054	0.022547688
XXXT-1	SPP1	25.66836929	18.58896065	0.007394106
XXXT-2	SPP1	24.94492531	18.36195374	0.010431052
XXXT-3	SPP1	24.94713211	17.94250107	0.007787462

Relative expression fold

Target Name	CONTROL-1	CONTROL-2	CONTROL-3	XXXT-1	XXXT-2	XXXT-3
BCL2	6.23045E-05	2.62695E-05	2.94E-06	3.38999E-06	9.06801E-06	
BIRC5	0.001105394	0.001956454	0.001540654	0.000158281	0.000327622	0.000128958
CCNA2	0.007899442	0.007645327	0.005678833	0.00097469	0.001290082	0.001362239
CD40LG						
CHEK1	3.81424E-06	0.002102925	0.000712425	0.000607972	0.000355866	0.000662333
CYCS	0.121933516	0.19347825	0.183350633	0.03674501	0.057506829	0.031401786
EGLN1	0.003900718	0.001972562	0.001556994	0.000959	0.000937923	0.001106095
FOSL1	0.015747438	0.007782645	0.008066496	0.006041681	0.005207382	0.005172703
FOSL2	0.003898315	0.003907376	0.002943665	0.002311581	0.001964951	0.002109823
HIF1A	0.002528781	0.004550875	0.002824933	0.003092527	0.004157757	0.006024532
HK2	1.83222E-05	6.15134E-05	4.00978E-05	9.85831E-06		

2.43561E-06   2.2827E-05

IL2   2.7089E-06

MMP3   3.3981E-06 7.68279E-06   3.39853E-06   0.000314561

0.000429189   0.000143025

NFATC1 0.000543252   0.00048786 0.000405488   0.000191401

0.000188155   0.000163861

PIK3CG   1.52948E-05   8.94045E-05   5.15733E-05

1.80265E-06

PRKCB   3.4324E-06   5.58587E-06   4.71536E-05

PTGS2   3.85982E-06   5.99647E-05   3.41692E-06   3.04587E-06

3.24664E-06   4.97849E-06

RELA   0.004867048   0.003912049   0.00398154 0.013485402

0.010482784   0.010234278

SERPINE1   0.00604801 0.003943079   0.002887383   0.012041834

0.01055196 0.011203268

SPP1   0.015463555   0.033569844   0.022547688   0.007394106

0.010431052   0.007787462

Fold change

Target Name   XXXT/CONTROL

BCL2   0.115925026

BIRC5   0.133592919

CCNA2   0.170895158

CD40LG #DIV/0!

CHEK1 0.576827215

CYCS 0.25193083

EGLN1 0.4041598

FOSL1 0.519732394

FOSL2 0.59411505

HIF1A 1.340269148

HK2 0.292836818

IL2 #DIV/0!

MMP3 61.24383522

NFATC1 0.378265776

PIK3CG 3.111743232

PRKCB 10.45734849

PTGS2 0.167619829

RELA 2.680310247

SERPINE1 2.624306731

SPP1 0.357812663

P value

Target Name XXXT/CONTROL Differences Annotation

BCL2 0.064340144 ns

BIRC5 0.006314909 **

CCNA2 0.001182154 **

CD40LG #DIV/0!#DIV/0!

CHEK1	0.55841741	ns
CYCS	0.006333948	**
EGLN1	0.111136773	ns
FOSL1	0.126184415	ns
FOSL2	0.012260878	*
HIF1A	0.35052968	ns
HK2	0.110215443	ns
IL2	#DIV/0!	#DIV/0!
MMP3	0.02497947	*
NFATC1	0.001901983	**
PIK3CG	#DIV/0!	#DIV/0!
PRKCB	#DIV/0!	#DIV/0!
PTGS2	0.376861154	ns
RELA	0.002788494	**
SERPINE1	0.002432392	**
SPP1	0.045827013	*
